# Bimetal Metaphosphate/Molybdenum Oxide Heterostructure Nanowires for Boosting Overall Freshwater/Seawater Splitting at High Current Densities

**DOI:** 10.1002/advs.202407892

**Published:** 2024-09-30

**Authors:** Pan Wang, Pai Wang, Tongwei Wu, Xuping Sun, Yanning Zhang

**Affiliations:** ^1^ Institute of Fundamental and Frontier Sciences University of Electronic Science and Technology of China Chengdu 610054 China; ^2^ School of Materials and Energy Guangdong University of Technology Guangzhou 510006 China; ^3^ College of Chemistry Chemical Engineering and Materials Science Shandong Normal University Jinan 250014 China

**Keywords:** bifunctional heterostructures, bimetal metaphosphates, freshwater/seawater splitting, high current densities, molybdenum oxides

## Abstract

Exploring excellent non‐noble bifunctional electrocatalysts for freshwater/seawater splitting at high current densities has attracted extensive interest owing to strong anodic oxidation and severe chloride corrosion challenges. Herein, hierarchical bimetal Ni‐Co metaphosphate/molybdenum oxide heterostructure nanowires (NiCoMoPO) are rationally designed and fabricated to efficiently boost oxygen evolution reaction (OER) and hydrogen evolution reaction (HER) in alkaline freshwater/seawater, where the favorable electronic structure from heterostructures, signified by X‐ray absorption spectra, endows NiCoMoPO with the enhanced intrinsic activity, while its hierarchical nanowire structure and heterostructures provide abundant active sites. Additionally, the PO_3_^−^ improves the chloride‐corrosion resistance and efficiently facilitates the OER kinetics verified by theoretical and experimental studies. Therefore, NiCoMoPO drives 1000 mA cm^−2^ at low overpotentials of 467 and 442 mV for OER and HER in alkaline freshwater respectively, as well as a small cell voltage of 2.135 V for overall freshwater splitting with robust durability of 300 h. Impressively, due to the strong corrosion resistance, at 500 mA cm^−2^ of overall seawater splitting, NiCoMoPO maintains almost 2.096 V for 1200 h, indicating promising practical applications. This work sheds light on the rational design and fabrication of outstanding electrocatalysts at high current densities of seawater/freshwater splitting.

## Graphical abstract

Due to the favorable electronic structure from the heterostructures and optimized Ni/Co molar ratio, 3D heterostructure nanowires endowed with abundant exposing active sites, and chloride‐repelling characteristics guaranteed by the electrostatic shielding of the PO_3_^−^ from the Cl^−^, 3D bimetal Ni‐Co metaphosphate/molybdenum oxide heterostructure nanowires exhibit excellent activities and stabilities for overall freshwater/seawater splitting at high current densities.

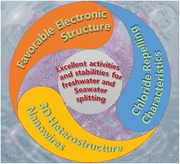

## 1 Introduction

With the rapid increment of energy consumption and environmental problems, hydrogen (H_2_), which serves as renewable‐green energy, has been considered an ideal substitute for fossil fuel. Fuel.^[^ ^1^ ^]^ Recently, H_2_ from electrochemical water splitting has been attracting great attention.^[^ ^2^ ^]^ Even so, key factors of the electrochemical‐water‐splitting technology in industrial applications are energy consumption and durability, i.e., cell voltages and stabilities, which are still under improvement.^[^ ^3^ ^]^ The cell voltages of water splitting mainly depend on the overpotentials of cathodic hydrogen evolution reaction (HER) and anodic oxygen evolution reaction (OER). Therefore, their low overpotentials can achieve small cell voltages, resulting in low energy consumption. On the other hand, the stabilities of electrocatalysts are an important criterion for commercial applications. Therefore, developing high‐activity and robust‐durability electrocatalysts for water splitting can efficiently solve the problem.

Up to now, noble metal materials, i.e., Pt and Ru/Ir, are still the state‐of‐the‐art electrocatalysts for water splitting.^[^ ^4^ ^]^ Nevertheless, their high cost and scarcity limit strictly their commercial applications. So, transition‐metal‐based electrocatalysts are good alternatives to those noble metal materials. Unfortunately, it is extraordinarily difficult to fabricate them with high activities and excellent durability, especially at high current densities (≥500 mA cm^−2^), due to the strong electrochemical oxidation and reduction corrosion at high anodic and cathodic potentials, respectively.^[^ ^5^ ^]^ As is known to all, the volume of seawater is ≈96.5% of the earth's water resources.^[^ ^6^ ^]^ Due to the lack of precious freshwater resources, seawater is an ideal alternative electrolyte for water splitting. However, in the seawater splitting process, apart from the anodic oxidation, chlorine evolution reaction (CER) can compete with OER at high anodic potentials to generate hypochlorite (ClO^−^), which not only decreases the efficiency of seawater splitting but also leads to bad durability of electrocatalysts owing to the chemical corrosion of ClO^−^.^[^ ^7^ ^]^ For alkaline seawater splitting, the theoretical potential of CER is ≈480 mV higher than that of OER.^[^ ^8^ ^]^ Hence, outstanding seawater‐splitting electrocatalysts are required to facilitate OER and suppress CER to acquire 100% efficiency of OER. Moreover, chloride ions (Cl^−^) can also corrode electrocatalysts to shorten their stabilities.^[^ ^7^ ^]^ For the above reasons, in the past few decades, intensive research efforts have gone into preparing all kinds of transition‐metal‐based electrocatalysts for accelerating the water‐splitting process in seawater electrolytes, including phosphides (e.g., Mn‐doped Ni_2_P/Fe_2_P),^[^ ^9^ ^]^ oxides (e.g., MnFe_2_O_4_),^[^ ^10^ ^]^ sulfides (e.g., NiFe/NiS_x_),^[^ ^11^ ^]^ hydroxides (e.g., CrO_4_^2−^‐NiFe LDH/Cr_2_O_3_),^[^ ^12^ ^]^ nitrides (e.g., MoN/Co_2_N),^[^ ^13^ ^]^ and carbon oxyanions (e.g., (NiFe)C_2_O_4_).^[^ ^14^ ^]^ However, these fabricated electrocatalysts cannot meet the requirements of industrial applications. Therefore, there remain widespread concerns for developing highly effective transition‐metal‐based electrocatalysts for water splitting at high current densities in seawater electrolytes.

To address these above challenges, electrocatalysts should be designed rationally to improve their intrinsic activities, structure stabilities, and chlorine‐corrosion resistance. First, the electronic structure optimization can promote HER and OER processes via interface engineering in heterostructures.^[^ ^15^ ^]^ Jiang et al. constructed interfacial Co/CoMoN heterostructures, exhibiting high HER and OER activities because of the interfacial heterostructures.^[^ ^16^ ^]^ In addition, Wang et al. synthesized a hetero‐interface electrocatalyst of oxygen vacancy enriched Co_3_O_4_ and crystalline‐amorphous NiFe LDH supported on nickel foam (NF).^[^ ^17^ ^]^ Owing to the interaction between Co and Fe species and the facilitated electron transfer from the hetero‐interface, the electrocatalyst only needs a low overpotential of 257 mV to achieve 500 mA cm^−2^, indicating outstanding OER activity. Moreover, hierarchical 3D electrocatalysts have large surface areas and excellent structure stability, resulting in abundant exposed active sites and robust durability.^[^ ^18^ ^]^ In our previous work, MnO_x_‐decorated 3D nickel‐iron phosphide nanosheets grown on NF not only own plentiful active sites, but also demonstrate superior structure stability in a long‐term chronopotentiometry measurement of 1000 mA cm^−2^ for overall water splitting.^[^ ^19^ ^]^ In addition, Dai et al. presented an excellent electrocatalyst of the Ni sulfide layer@Ni‐Fe hydroxide layer, proving that in the OER process, polyatomic sulfate and carbonate‐rich passivating layers can be in situ generated, which can repel Cl^−^, then promote the chlorine corrosion resistance.^[^ ^11^ ^]^ Therefore, polyatomic anions, such as phosphate, sulfate, and carbonate, may effectively resist chlorine corrosion to sustain the physical structure of electrocatalysts for improving their seawater‐splitting durability.

Based on the above considerations, herein, 3D hierarchical bimetal metaphosphate/molybdenum oxide (Ni_x_Co_1−x_(PO_3_)_2_/MoO_x_, NiCoMoPO) heterostructure nanowires grown on NF were rationally designed and fabricated by simple hydrothermal and subsequent phosphatization treatments. The 3D nanowire structure and heterostructures of NiCoMoPO provide enormous quantities of active sites, while the heterostructures optimize the electronic structure to improve the inherent activities of HER and OER. Moreover, the PO_3_^−^ can hinder Cl^−^ from corroding the electrocatalyst via the electrostatic shield to enhance its durability for seawater splitting. Thereby, this electrocatalyst exhibits outstanding bifunctional activity and durability at high current densities in alkaline freshwater/seawater.

## 2 Results and Discussion

### 2.1 Synthesis and Structural Characterization

**Figure 1:**
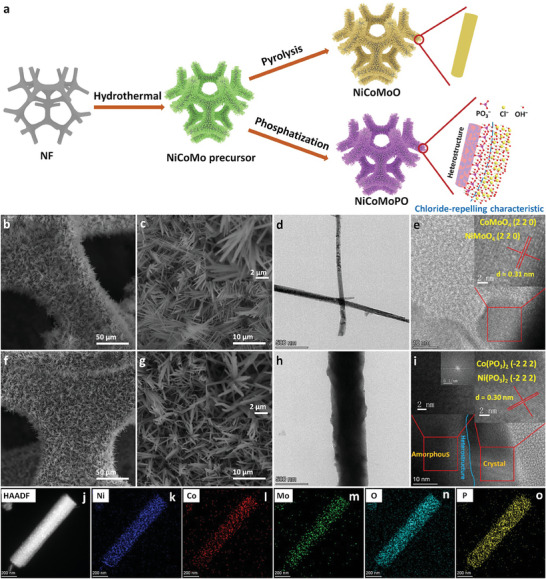
a) Schematic illustration of the synthesized process of electrocatalysts. SEM and TEM images of b–e) NiCoMoO and f–i) NiCoMoPO. j–o) HAADF‐STEM and the corresponding elemental mapping images of NiCoMoPO.

As shown in **Figure** **1**a, the synthesized procedures of NiCoMoPO and bimetal molybdate (Ni_x_Co_1−x_MoO_4_, identified as “NiCoMoO”) are presented. First, 3D hierarchical NiCoMo precursor nanowires were uniformly grown on NF through a simple hydrothermal strategy. Figure S1 (Supporting Information) displays dense and interlaced nanowires of NiCoMo precursor with a 3D hierarchical structure. Then, NiCoMoO can be obtained from the precursor via facile pyrolysis in an Ar flow. Figure 1b,c exhibits that 3D hierarchical crisscross nanowires of NiCoMoO are densely grown on the surface of NF, demonstrating that the pyrolysis has almost no effects on the 3D hierarchical nanowire morphology. Meanwhile, by a facile phosphatization method, the NiCoMo precursor was transformed into NiCoMoPO, which also maintains almost the original 3D hierarchical structure (Figure 1f,g), which has a big advantage of exposing substantial active sites to improve the water‐splitting activity. The elemental composition of NiCoMoPO was analyzed by energy‐dispersive X‐ray spectroscopy (EDS) in scanning electron microscope (SEM) equipment, and the corresponding elemental mapping images (Figure S2, Supporting Information) verifies uniform dispersal of Ni, Co, Mo, P, and O elements, implying the success of the phosphatization strategy for NiCoMo precursor.

The morphology and composition of NiCoMoO and NiCoMoPO were further analyzed by transmission electron microscopy (TEM). Figure 1d,h further testifies to the nanowire structures of NiCoMoO and NiCoMoPO, respectively. Then, the high‐resolution TEM (HRTEM) image (Figure 1e) of NiCoMoO exhibits distinct lattice planes, revealing its crystalline characteristic. Figure S3 (Supporting Information) demonstrates clear diffraction rings of NiCoMoO via the selected area electron diffraction (SAED), further proving its crystalline feature. Meanwhile, the inset in the upper right corner of Figure 1e shows an interplanar spacing of ≈0.31 nm, which is consistent with the (2 2 0) lattice planes of NiMoO_4_ (ICDD PDF#33‐0948) and CoMoO_4_ (ICDD PDF#25‐1434). Therefore, NiCoMoO consists of crystalline NiMoO_4_ and CoMoO_4_. The HRTEM image (Figure 1i) of NiCoMoPO presents no obvious lattice fringes in the left area, indicating its amorphous structure. Moreover, the SAED pattern in the upper left inset obtained in the left marked area of Figure 1i via the fast Fourier transform (FFT) exhibits no distinct diffraction spots or rings, further implying the amorphous composition. Nevertheless, in the right area of Figure 1i, clear lattice planes of ≈0.30 nm, ascribed to the (−2 2 2) interplanar planes of Ni(PO_3_)_2_ (ICDD PDF#28‐0708) and Co(PO_3_)_2_ (ICDD PDF#27‐1120), can be observed. The corresponding SAED pattern (Figure S4, Supporting Information) demonstrates two symmetric diffraction spots, which proves the crystal nature in the right area of the blue line in Figure 1i. Moreover, diffraction spots in the SAED pattern (Figure S5, Supporting Information) of NiCoMoPO also verify the crystalline structure of Ni(PO_3_)_2_ and Co(PO_3_)_2_. As a result, an obvious interface marked by the blue line in Figure 1i exists between the amorphous and crystalline phases, manifesting the formation of heterostructures in the NiCoMoPO hybrid. Meanwhile, the high‐angle annular dark‐field scanning TEM (HAADF‐STEM) elemental mapping analysis result (Figure 1j–o) from TEM equipment clarifies that Ni, Co, Mo, O, and P elements exist homogeneously in the nanowire of NiCoMoPO. Moreover, as shown in Figure S6 (Supporting Information), line‐scan electron energy loss spectroscopy (EELS) is also conducted to analyze its element spatial distribution. The elemental profiles of NiCoMoPO further exhibit the homogeneous spatial distribution of Ni, Co, Mo, O, and P elements along the nanowire. Besides, because MoO_4_^2−^ is difficult to transform into molybdenum phosphides under low phosphorization temperatures,^[^ ^20^ ^]^ the amorphous phase in the left area of the blue line may be MoO_x_. Based on the above result, NiCoMoPO may be composed of crystalline Ni_x_Co_1−x_(PO_3_)_2_ and amorphous MoO_x_, where heterostructures exist in the interfaces, which has a great advantage of optimizing the electronic structure and exposing more active sites of catalysts.^[^ ^21^ ^]^

**Figure 2:**
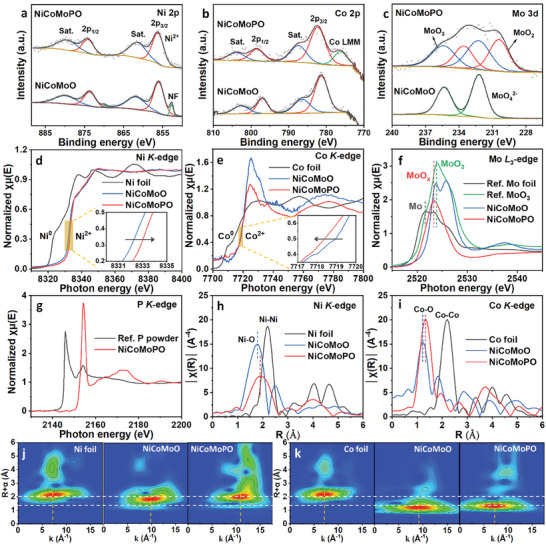
XPS spectra of a) Ni 2p, b) Co 2p, and c) Mo 3d for NiCoMoO and NiCoMoPO. d) Ni *K*‐edge XANES spectra of reference Ni foil, NiCoMoO, and NiCoMoPO. e) Co *K*‐edge XANES spectra of reference Co foil, NiCoMoO, and NiCoMoPO. f) Mo *L*_3_‐edge XANES spectra of NiCoMoO, NiCoMoPO, reference Mo foil, and reference MoO_3_. g) P *K*‐edge XANES spectra of NiCoMoPO and reference P powder. h) Ni *K*‐edge k^3^‐weighted FT‐EXAFS spectra of reference Ni foil, NiCoMoO, and NiCoMoPO. i) Co *K*‐edge k^3^‐weighted FT‐EXAFS spectra of reference Co foil, NiCoMoO, and NiCoMoPO. j) WT images of Ni *K*‐edge for reference Ni foil, NiCoMoO, and NiCoMoPO. k) WT images of Co *K*‐edge for reference Co foil, NiCoMoO, and NiCoMoPO.

Subsequently, X‐ray diffraction (XRD) and X‐ray photoelectron spectroscopy (XPS) were applied to study the crystal‐phase structures and chemical composition of as‐prepared samples. As shown in Figure S7 (Supporting Information), three typical peaks marked by asterisks come from the metallic nickel of NF. For NiCoMo precursor, no obvious related phase is detected, whereas it was transformed into NiMoO_4_ (ICDD PDF#33‐0948) and CoMoO_4_ (ICDD PDF#25‐1434) after the pyrolysis treatment in an air flow, manifesting NiCoMoO is made up of NiMoO_4_ and CoMoO_4_, which is identical with the result of TEM. Besides, the XRD pattern of NiCoMoPO shows diffraction peaks of Ni(PO_3_)_2_ (ICDD PDF#28‐0708) and Co(PO_3_)_2_ (ICDD PDF#27‐1120), but no obvious diffraction peaks of MoO_x_, which accords with its TEM result of the amorphous phase MoO_x_ and crystalline components (Figure 1i). Moreover, XPS survey spectra (Figure S8, Supporting Information) of NiCoMoPO and NiCoMoO further testify that Ni, Co, Mo, O, and P elements exist, and no other elemental peaks can be observed, signifying their high purities. In the XPS Ni 2p spectra (**Figure** **2**a) of NiCoMoPO and NiCoMoO, a peak located at 852.5 eV comes from metallic nickel of NF, while the spin‐orbit doublet of Ni 2p_3/2_/2p_1/2_ (856.3/874.0 eV) can be attributed to the Ni^2+^, together with the corresponding shake‐up satellites at 861.9 and 879.9 eV, referred to as “Sat.”.^[^ ^22^ ^]^ Concerning the XPS Co 2p spectrum of NiCoMoPO (Figure 2b), the peak at 776.2 eV is from the Co L_3_M_23_M_45_ Auger peak, which overlaps part of the Co 2p region.^[^ ^23^ ^]^ In addition, the doublet peaks at 782.3/787.4 eV are assigned to Co 2p_3/2_/2p_1/2_ of Co^2+^ in Co(PO_3_)_2_, followed by its corresponding Sat. peaks appearing at 798.4 and 803.9 eV.^[^ ^24^ ^]^ For NiCoMoO, the doublet peaks (781.3/786.5 eV) correspond to the spin‐orbit characteristic of CoMoO_4_, and its Sat. peaks appear at 797.0 and 802.7 eV.^[^ ^25^ ^]^ In the XPS Mo 3d spectrum (Figure 2c) of NiCoMoO, its doublet peaks of Mo 3d_5/2_/3d_3/2_ can be observed at 232.2/235.33 eV, which is associated with the Mo^6+^ of MoO_4_^2−^.^[^ ^22^, ^26^ ^]^ Whereas, the XPS Mo 3d curve of NiCoMoPO is deconvoluted into two spin‐orbit doublets, observed at 230.4/233.6 eV and 232.3/235.4 eV, which are associated with Mo^4+^ of MoO_2_ and Mo^6+^ of MoO_3_, respectively,^[^ ^27^ ^]^ clarifying that the Mo species of NiCoMo precursor was pyrolyzed and reduced into Mo‐based oxides (MoO_x_) of mixed MoO_2_ and MoO_3_ in PH_3_ gas atmosphere from decomposed NaH_2_PO_2_·H_2_O under high temperatures. With regard to O 1s (Figure S9, Supporting Information), for NiCoMoPO, the peak at 531.5 eV belongs to MoO_x_,^[^ ^28^ ^]^ and the other peak at 533.2 eV corresponds to PO_3_^−^.^[^ ^24^, ^27^ ^]^ At the same time, two peaks of NiCoMoO at 529.8 and 530.7 eV are related to Ni/Co oxides and MoO_4_^2−^, respectively.^[^ ^25^, ^29^ ^]^ In P 2p (Figure S10, Supporting Information) of NiCoMoPO, the doublet peaks (P 2p_3/2_/2p_1/2_) located at 134.1/135.0 eV are assigned to PO_3_^−^, verifying the existence of metal metaphosphates.^[^ ^22^, ^24^ ^]^ Accordingly, the above results reveal the successful preparation of NiCoMoPO comprising crystalline Ni_x_Co_1−x_(PO_3_)_2_ and amorphous MoO_x_.

To deeply explore the local electronic structure and coordinative geometry of NiCoMoO and NiCoMoPO, X‐ray absorption fine structure (XAFS) spectra were conducted.^[^ ^30^ ^]^ Figure 2d shows X‐ray absorption near edge structure (XANES) spectra for Ni *K*‐edge of the reference Ni foil, NiCoMoO, and NiCoMoPO. The Ni *K*‐edge absorption position of NiCoMoPO is very close to that of NiCoMoPO, but much higher than that of Ni foil, indicating that the chemical valences of Ni in both NiCoMoO and NiCoMoPO may be +2. To further confirm the Ni valence, soft XANES spectra of Ni *L*‐edge for reference NiCl_2_ and NiCoMoPO were obtained. The white‐line shapes greatly depend on the multiplet structures given by the Ni 2p → 3d interactions and crystal field effects.^[^ ^31^ ^]^ As shown in Figure S11 (Supporting Information), the peak position and white line shape of the spectrum for NiCoMoPO are very similar to those of reference NiCl_2_, signifying that the chemical valence of Ni in NiCoMoPO is +2, which corresponds to the Ni 2p XPS result (Figure 2a). Based on the Ni *K*‐edge result, the Ni element in NiCoMoO is also Ni^2+^ ions. For Co *K*‐edge (Figure 2e), NiCoMoO and NiCoMoPO have the same absorption position, much higher than that of Co foil, indicating the same oxidation state of Co in both NiCoMoO and NiCoMoPO. Moreover, Co *L*‐edge XANES spectra of reference CoCl_2_ and NiCoMoPO are displayed in Figure S12 (Supporting Information). They own almost the same peak positions with a characteristic peak of Co^2+^ at 777.1 eV and a similar white line shape with five peaks for Co *L*_3_‐edge,^[^ ^32^ ^]^ demonstrating their Co elements are the chemical valence of +2. Moreover, Figure 2e displays different white‐line shapes of NiCoMoO and NiCoMoPO, verifying their different Co local coordination environments. Compared to NiCoMoO, Ni and Co *K*‐edge positions of NiCoMoPO shift slightly to higher and lower photon energies (the insets in Figure 2d,e) respectively, which could be ascribed to that the transformation from Ni_x_Co_1−x_MoO_4_ to Ni_x_Co_1−x_(PO_3_)_2_/MoO_x_ causes the electron transfer from Ni to Co, signifying the synergistic effect of NiCoMoPO, benefiting optimizing its electronic structure, then improving its intrinsic water‐splitting activity. Figure 2f and Figure S13 (Supporting Information) show Mo *L*‐edge XANES spectra for NiCoMoO, NiCoMoPO, reference Mo foil, and reference MoO_3_. The Mo *L*_3_‐edge and *L*_2_‐edge positions of NiCoMoO are much close to those of reference MoO_3_, revealing that the chemical valence of Mo in NiCoMoO is +6, in accord with the Mo 3d XPS result (Figure 2c). The Mo *L*_3_‐edge position of NiCoMoPO is far away from that of reference Mo foil, but lower than that of NiCoMoO, manifesting the chemical valence of Mo of MoO_x_ in NiCoMoO is below +6, which is attributed to the co‐existence of MoO_2_ and MoO_3_ in MoO_x_. Moreover, in the Mo *L*_3_‐edge XANES spectra, the white‐line shape of MoO_x_ in NiCoMoPO is similar to that of reference MoO_3_, proving the MoO_x_ has the same octahedral structure as reference MoO_3_,^[^ ^33^ ^]^ while MoO_4_^2−^ has a tetrahedral structure.^[^ ^33^ ^]^ So, NiCoMoO has a completely different white‐line shape from the MoO_x_ and reference MoO_3_. Because different electronic structures of Mo lead to different intrinsic water‐splitting activities, the MoO_x_ of NiCoMoPO may own a favorable electronic structure for water splitting. For P *K*‐edge XANES spectra (Figure 2g), the *K*‐edge absorption position of NiCoMoPO is much higher than that of reference P powders, which can be assigned to the chemical valence of +5 for P in PO_3_^−^. Furthermore, k^3^‐weighted Fourier transform‐extended X‐ray absorption fine structure (FT‐EXAFS) spectra were applied to analyze the coordination environments of as‐fabricated samples. Figure 2h displays Ni *K*‐edge FT‐EXAFS spectra of NiCoMoO, NiCoMoPO, and Ni foil. For Ni foil, the maximum intensity peak at 2.21 Å comes from Ni–Ni bonds. The NiMoO_4_ in NiCoMoO has one main peak of Ni–O bonds at 1.78 Å,^[^ ^34^ ^]^ while the main peak of Ni–O bonds for the Ni(PO_3_)_2_ in NiCoMoPO is located at 1.87 Å. For Co *K*‐edge FT‐EXAFS (Figure 2i), the main peak of Co foil, associated with Co–Co bonds, is located at 2.18 Å. The CoMoO_4_ in NiCoMoO and the Co(PO_3_)_2_ in NiCoMoPO have one main peak of Co–O bonds at 1.23 and 1.34 Å, respectively. The above results indicate that owing to the replacement of a partial quantity of O in NiCoMoO by P, the lengths of Ni–O and Co–O bonds are slightly elongated in NiCoMoPO, causing the electron redistribution of Ni and Co atoms to adjust the electronic structure of NiCoMoPO, which is beneficial to optimizing the intrinsic HER and OER activities. Furthermore, the wavelet transform (WT) was applied to further clarify the Ni and Co *K*‐edges EXAFS oscillation of NiCoMoO and NiCoMoPO in both R and k space resolution.^[^ ^35^ ^]^ Figure 2j shows that the WT image of Ni foil exhibits one maximum intensity at ≈7.2 Å^−1^/2.2 Å, which is from the contribution of Ni–Ni bonds, while those of NiCoMoO and NiCoMoPO appear at ≈9.9 Å^−1^/1.8 Å and 11.0 Å^−1^/2.0 Å respectively, assigned to the contributions of the Ni–O bonds. Regarding the Co *K*‐edge (Figure 2k), Co foil has one maximum intensity at ≈7.2 Å^−1^/2.2 Å, related to the contribution of Co–Co bonds, while for NiCoMoO and NiCoMoPO, their maximum intensities at ≈9.4 Å^−1^/1.2 Å and 7.0 Å^−1^/1.4 Å respectively, which are both related to the contribution of Co–O bonds. It is worth noting that positions (i.e., R and k values) of the maximum intensity in WT images are mainly associated with the bond length and atomic number of scattering atoms.^[^ ^36^ ^]^ Compared to Ni/Co foil and Ni/CoMoO_4_, the Ni(PO_3_)_2_ and Co(PO_3_)_2_ in NiCoMoPO have many different R and k values, indicating their entirely different geometric coordination for Ni and Co in NiCoMoPO, namely completely different surface electronic structures for Ni and Co. Consequently, compared to NiCoMoO, NiCoMoPO have totally different coordination geometries of Ni, Co, and Mo atoms, indicating the different crystalline structures and the formation of heterostructures, leading to the optimized electronic structures, which could cause a favorable surface electronic structure for NiCoMoPO to enhance its intrinsic activities for HER and OER in the water‐splitting process.

**Figure 3:**
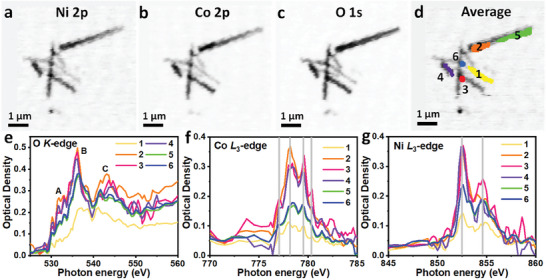
STXM elemental optical density maps of NiCoMoPO derived from a) Ni and b) Co *L*‐edges, and c) O *K*‐edge. d) Average STXM image of Ni 2p, Co 2p, and O 1s for NiCoMoPO. The spatially resolved STXM‐XANES spectra of e) O *K*‐edge, f) Co *L*_3_‐edge, and g) Ni *L*_3_‐edge extracted from different selected regions (marked by 1, 2, 3, 4, 5, and 6) of interest in Figure 3d.

To further reveal the heterostructures and synergistic effect in NiCoMoPO, scanning transmission X‐ray microscopy (STXM) chemical imaging was conducted by using high brilliance synchrotron radiation to obtain spectroscopic information of the electronic structure of atoms and molecules.^[^ ^37^ ^]^ **Figure** **3**a–c exhibits STXM elemental optical density maps of Ni 2p, Co 2p, and O 1s for NiCoMoPO respectively, derived from the corresponding Ni *L*‐edge, Co *L*‐edge, and O *K*‐edge, respectively. These element maps show the almost same shape and contrast, and no obvious lack of Ni, Co, and O elements on NiCoMoPO nanowires, indicating their similar spatial distribution along the nanowires. By extracting the different selected regions (marked by 1, 2, 3, 4, 5, and 6 in Figure 3d) data of interest, the spatially resolved STXM‐XANES spectra of O *K*‐edge, Co *L*_3_‐edge, and Ni *L*_3_‐edge were obtained, as shown in Figure 3e–g, respectively. For O *K*‐edge (Figure 3e), the peaks labeled with A at ≈531.8 eV can be associated with the electron transition of O 1s → Ni/Co 3d–O 2p and Mo 4d–O 2p hybrid unoccupied orbits.^[^ ^38^ ^]^ Besides, the B peaks at ≈536.2 eV can be ascribed to the electron transition from O 1s to P 3p–O 2p hybrid orbits.^[^ ^38^, ^39^ ^]^ The intensities of A peaks in 1, 5/6, 3/4, and 2 regions are obviously different, testifying to the existence of different O–M (M = Ni, Co, and Mo) coordination distortions in those corresponding regions. In addition, the 1, 5/6, and 2/3/4 regions have different B peak intensities, verifying the appearance of different O–P coordination distortions in the corresponding regions. The above distortion results may be attributed to that the heterogeneous spatial nanoscale distribution for Ni(PO_3_)_2_, Co(PO_3_)_2_, and MoO_x_ in NiCoMoPO nanowires generates different coordination environments, which can form the heterostructures, leading to the synergistic effect among them, then optimizing the electronic structure of NiCoMoPO to improve the inherent OER and HER activities. Furthermore, the absorption C peaks, located at ≈542.8 eV, are associated with the electron transition from O 1s to the hybridized orbits of the unoccupied O 2p orbit and the outermost Ni/Co 4sp and Mo 5sp character orbits.^[^ ^38b^ ^]^ By comparing the positions, intensities, and white‐line shapes of C peaks in 1, 5/6, 3/4, and 2 regions, they are different from each other owing to different multiple scattering processes, indicating different O–M (M = Ni, Co, and Mo) coordination geometries in those regions, which is agreement with the result of the A peaks. Consequently, nonuniformly spatial nanoscale distribution of Ni(PO_3_)_2_, Co(PO_3_)_2_, and MoO_x_ in NiCoMoPO nanowires leads to constructing heterostructures and forming different coordination environments of Ni, Co, and Mo in NiCoMoPO, which can generate the synergistic effect in NiCoMoPO to enhance its HER and OER intrinsic activities. Figure 3f,g displays Co and Ni *L*_3_‐edges XANES spectra of those regions in Figure 3d, respectively. The absorption peaks for Co and Ni *L*_3_‐edges can be ascribed to the electron transition of 2p_3/2_ → 3d orbits of Co and Ni ions.^[^ ^34^, ^40^ ^]^ These peaks in all regions have approximately the same positions and white‐line shapes except the intensities, testifying that for Co and Ni‐based composites in NiCoMoPO, only the Co(PO_3_)_2_ and Ni(PO_3_)_2_ components exist, while no impurity components, such as Ni_2_P and CoP, exist, indicating the high purity of as‐prepared NiCoMoPO. Moreover, for Co *L*_3_‐edge (Figure 3f), the 5 and 6 regions have the almost same white‐line shape and intensity, verifying that the two regions own the nearly same Co coordination environment. Similarly, the 2, 3, and 4 regions possess the same result. Nonetheless, the white‐line shapes of the 1, 5/6, and 2/3/4 regions are very different, indicating the different coordination environments of Co ions in those different regions. Interestingly, the Ni *L*_3_‐edge (Figure 3g) shows the same result as the Co *L*_3_‐edge. As a result, for NiCoMoPO, although O, Co, and Ni elements seem to have uniform spatial distribution in the nanowires, they have different coordination environments in the different local regions of nanowires, which can be ascribed to the existence of numerous heterostructures, resulting in optimizing the electronic structure of NiCoMoPO to improve the inherent HER and OER activities of NiCoMoPO.

### 2.2 Electrocatalytic Performance for OER and HER in Alkaline Freshwater

**Figure 4:**
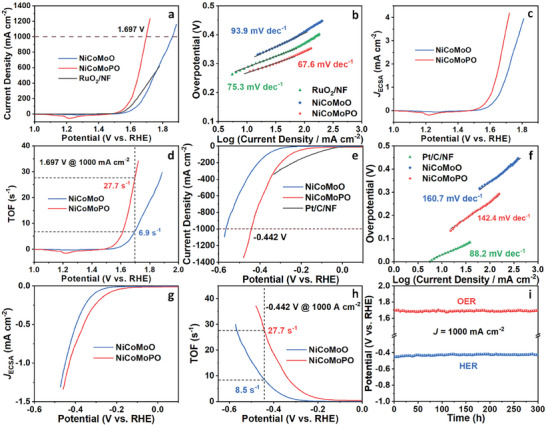
a) OER LSV curves of NiCoMoO, NiCoMoPO, and RuO_2_/NF. b) The corresponding OER Tafel plots. c) The corresponding OER LSV curves standardized by ECSA, and d) OER TOF plots for NiCoMoO and NiCoMoPO. e) HER LSV curves of NiCoMoO, NiCoMoPO, and Pt/C/NF. f) The corresponding HER Tafel plots. g) The corresponding HER LSV curves standardized by ECSA, and h) HER TOF plots for NiCoMoO and NiCoMoPO. i) Chronopotentiometry curves of NiCoMoPO at 1000 mA cm^−2^ for OER and HER. All tests were conducted in 1.0 M KOH freshwater.

Subsequently, OER performances of NiCoMoO and NiCoMoPO were estimated in 1.0 M KOH freshwater. First, for optimizing the preparing condition of NiCoMoPO, Figure S14 (Supporting Information) shows OER linear sweep voltammetry (LSV) curves of NiCoMoPO with Ni/Co molar ratio of 1:1 at different phosphatization temperatures, indicating that 350 °C is the best temperature for the phosphatization process. By comparing the OER LSV curves of NiCoMoPO with a Ni/Co molar ratio of 1:1 and more Mo (Figure S15, Supporting Information), it is found that larger Mo content decreases the OER activity of NiCoMoPO, which may be ascribed to that the MoO_x_ of NiCoMoPO is not the main active phase of OER. Therefore, the molar content of the Mo and Ni/Co is preferably equal to make the Ni/Co ions and MoO_4_^2−^ form Ni_x_Co_1−x_MoO_4_ precipitates. To optimize the molar ratio of Ni:Co in NiCoMoPO, five samples with different molar ratios were prepared. As shown in Figure S16 (Supporting Information), their OER LSV curves demonstrate that the best Ni/Co molar ratio is 2:1. Then, the OER activity of the optimized NiCoMoPO was compared with those of NiCoMoO and RuO_2_/NF via LSV curves (**Figure** **4**a). NiCoMoPO owns the best OER activity with a low overpotential of 467 mV at an ultra‐high current density of 1000 mA cm^−2^. Further, Figure S17 (Supporting Information) compares their OER activities at different current densities. NiCoMoPO requires small overpotentials of 272, 342, 419, and 467 mV to reach 10, 100, 500, and 1000 mA cm^−2^ respectively, which are considerably lower than those of NiCoMoO (318, 410, 518, and 631 mV at 10, 100, 500, and 1000 mA cm^−2^, respectively) and RuO_2_/NF (286, 360, and 463 mV at 10, 100, and 500 mA cm^−2^, respectively), manifesting its excellent OER activity in alkaline freshwater, which outperforms most non‐noble electrocatalysts, particularly at high current densities of 500 and 1000 mA cm^−2^ (Table S1, Supporting Information). Such outstanding OER activity can be attributed to that the optimized electronic structure of NiCoMoPO improves its intrinsic OER activity, while the 3D nanowire structure benefits exposing plentiful active sites and accelerating generated bubbles removing from the surface of NiCoMoPO. Moreover, these samples’ kinetics were studied by the corresponding Tafel slopes and Nyquist plots. Figure 4b shows that NiCoMoPO has a smaller Tafel slope of 67.6 mV dec^−1^ than NiCoMoO (93.9 mV dec^−1^) and RuO_2_/NF (75.3 mV dec^−1^), proving that NiCoMoPO possesses better kinetics than NiCoMoO and RuO_2_/NF. Meanwhile, the charge‐transfer resistance (*R*_ct_) of 3.2 Ω for NiCoMoPO is much lower than that of NiCoMoO (9.2 Ω) (Figure S18, Supporting Information), also testifying to the better kinetics and faster charge‐transfer ability for NiCoMoPO. Consequently, owing to the optimized electronic structure, NiCoMoPO exhibits favorable kinetics, leading to the enhanced intrinsic activity of OER.

In addition, their electrocatalytic active surface areas (ECSAs) were estimated by their electrochemical double layer capacitance (*C*_dl_) derived from cyclic voltammetry (CV) curves.^[^ ^41^ ^]^ Figure S19 (Supporting Information) shows CV curves of NiCoMoO and NiCoMoPO, and their corresponding *C*_dl_ plots (Figure S20, Supporting Information) demonstrate that the *C*_dl_ of NiCoMoPO is 5.6 mF cm^−2^, which is larger than that of NiCoMoO (4.1 mF cm^−2^), manifesting larger ECSA for NiCoMoPO. Based on SEM and TEM results of NiCoMoPO and NiCoMoO (Figure 1), their morphologies are the almost same 3D nanowire structure, meaning that their geometric surface areas are almost equal. Accordingly, compared to NiCoMoO, the larger ECSA of NiCoMoPO comes from more exposed active sites of OER, which reveals that preferable heterostructures of NiCoMoPO are beneficial to affording more OER active sites. For further comparing their OER intrinsic activities, their LSV curves in Figure 4a are standardized by the corresponding ECSAs, as shown in Figure 4c. NiCoMoPO has better inherent activity for OER than NiCoMoO. Then, their turnover frequency (TOF) values were calculated based on CV curves in Figure S21 (Supporting Information). TOF plots (Figure 4d) exhibit that NiCoMoPO owns a much larger TOF value than NiCoMoO, especially at high potentials. At 1.697 V, NiCoMoPO can drive an ultra‐large current density of 1000 mA cm^−2^, and the corresponding TOF value is 27.7 s^−1^, which is 4.0 times as large as that of NiCoMoO (6.9 s^−1^), further verifying its extremely higher OER inherent activity. Thereby, the optimized electronic structure of NiCoMoPO can incredibly promote its OER inherent activity.

Motivated by the outstanding OER performance of NiCoMoPO, its HER performance was carried out in the same alkaline freshwater. Figure S22 (Supporting Information) shows NiCoMoPO with the 2:1 molar ratio of Ni/Co owns the best HER activity among as‐fabricated NiCoMoPO samples with different molar ratios of Ni/Co. Afterward, Figure 4e shows noble Pt/C/NF has the best HER activity with an overpotential of 146 mV at 100 mA cm^−2^. For NiCoMoPO, it can afford an ultra‐high HER current density of 1000 mA cm^−2^ at a low overpotential of 442 mV, presenting better HER activity than NiCoMoO. Moreover, by comparing overpotentials, NiCoMoPO needs low overpotentials of 251, 368, and 442 mV to obtain 100, 500, and 1000 mA cm^−2^ respectively, whereas NiCoMoO requires much higher overpotentials of 364, 485, and 566 mV respectively (Figure S23, Supporting Information). Such superb HER activity surpasses most non‐noble electrocatalysts, particularly at high current densities of 500 and 1000 mA cm^−2^ (Table S2, Supporting Information), which is associated with the optimized electronic structure and 3D nanowire morphology. Concerning the HER kinetics (Figure 4f; Figure S24, Supporting Information), NiCoMoPO owns lower Tafel slope (142.4 mV dec^−1^) and *R*_ct_ (3.3 Ω) than those of NiCoMoO (160.7 mV dec^−1^ and 17.4 Ω), signifying that NiCoMoPO has faster kinetics and facilitated charge transfer, which reveals its higher inherent activity for HER due to the favorable electronic structure.

Moreover, Figures S25 and S26 (Supporting Information) show that NiCoMoPO possesses larger *C*_dl_ (16.9 mF cm^−2^) than NiCoMoO (7.6 mF cm^−2^). Therefore, NiCoMoPO has 2.2 times the ECSA of NiCoMoO. Because of their almost same shapes, the larger ECSA implies that the electronic structure of NiCoMoPO benefits exposing more active sites for HER. The current densities standardized by ECSAs are exhibited in Figure 4g. NiCoMoPO presents higher HER intrinsic activity than NiCoMoO. Their TOF plots (Figure 4h) demonstrate that NiCoMoPO has a larger TOF value than NiCoMoO. At −0.442 V (1.0 A cm^−1^), the TOF value of NiCoMoPO (27.7 s^−1^) is 3.3 times as large as NiCoMoO (8.5 s^−1^), testifying to its outstanding HER intrinsic activity because of the optimized electronic structure.

Durability is an extraordinarily important characteristic of electrocatalysts for practical applications. Figures S27 and S28 (Supporting Information) display multi‐current processes of NiCoMoPO for OER and HER in a range of 100–500 mA cm^−2^, respectively. All potentials can keep stable for 1000 s in each stair, revealing that NiCoMoPO owns robust mechanical and electrochemical stability for both OER and HER in the dramatic changing processes of high current densities. Furthermore, regarding chronopotentiometry tests at 100 and 500 mA cm^−2^ for OER and HER (Figures S29–S31, Supporting Information), it can sustain long‐term stabilities of more than 170 h. Impressively, at an ultra‐large current density of 1000 mA cm^−2^ for OER and HER, NiCoMoPO can maintain almost steady OER and HER potentials for 300 h (Figure 4i), suggesting its unbelievable stability, which exceeds almost all non‐noble electrocatalysts for OER and HER at high current densities (≥500 mA cm^−2^) (Tables S3 and S4, Supporting Information). The above stability results indicate the extremely excellent durability of NiCoMoPO. As is known to all, to drive high current densities, especially OER, electrocatalysts need to resist electrochemical corrosion at high potentials, which easily causes component change and structure collapse, leading to inferior durability. However, NiCoMoPO can maintain superior durability at ultra‐large current densities, such as 500 and 1000 mA cm^−2^, for a long time, implying its great potential for commercial applications.

### 2.3 Electrocatalytic Performance for OER and HER in Alkaline Seawater

**Figure 5:**
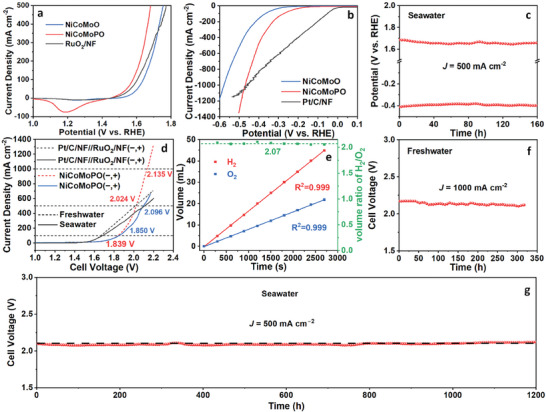
a) OER LSV tests of NiCoMoO, NiCoMoPO, and RuO_2_/NF in alkaline seawater. b) HER LSV tests of NiCoMoO, NiCoMoPO, and Pt/C/NF in alkaline seawater. c) Chronopotentiometry curves of NiCoMoPO for OER and HER at 500 mA cm^−2^ in alkaline seawater. d) LSV tests of overall water splitting for NiCoMoPO (−, +) and Pt/C/NF//RuO_2_/NF (−, +) in both alkaline freshwater and seawater. e) Faradic efficiency measurement by the volumes and corresponding ratio of collected H_2_ and O_2_ via a drainage method. f) Chronopotentiometry test for overall freshwater splitting of NiCoMoPO at 1000 mA cm^−2^. g) Chronopotentiometry test for overall seawater splitting of NiCoMoPO at 500 mA cm^−2^.

Due to the excellent performance of NiCoMoPO in alkaline freshwater, its water‐splitting performance was also evaluated in alkaline seawater (1.0 M KOH). As shown in **Figure** **5**a, compared with NiCoMoO and RuO_2_/NF, NiCoMoPO owns better OER activity in alkaline seawater with a low overpotential of 250 mV at 10 mA cm^−2^. Regarding the HER activity (Figure 5b), Pt/C/NF shows the best activity among all samples in the low HER potential region (0 ≈ −0.45 V), indicating its excellent intrinsic activity for HER. However, in the high potential region, NiCoMoPO possesses better activity than NiCoMoO and Pt/C/NF, which can be ascribed that 3D nanowire structure has an advantage of the rapid release of generated gas bubbles and sufficient contact with the electrolyte, while NiCoMoPO has favorable electronic structure for HER compared to NiCoMoO. Moreover, Figure S32 (Supporting Information) displays that to drive 100 and 500 mA cm^−2^ in alkaline seawater, NiCoMoPO only needs low overpotentials for OER (349 and 452 mV, respectively) and HER (275 and 404 mV, respectively). Particularly, it can afford 1000 mA cm^−2^ for HER at a low overpotentials of 470 mV, signifying superb activity for HER at ultra‐high current densities. Tables S5 and S6 (Supporting Information) compares respectively OER and HER overpotentials of NiCoMoPO and recently reported non‐noble electrocatalysts in alkaline seawater at different high current densities, e.g., 500 and 1000 mA cm^−2^, which validate that the OER and HER activities of NiCoMoPO outperform most non‐precious electrocatalysts, indicating that NiCoMoPO has outstanding activities for OER and HER in alkaline seawater. Then, its durability was tested in alkaline seawater (Figure 5c). NiCoMoPO can maintain almost unchanged potentials of both OER and HER for 160 h at 500 mA cm^−2^, revealing its high chlorine‐corrosion resistance and excellent electrochemical stability in the OER and HER processes of seawater splitting. Chen et al. reported that through the electrostatic repulsion, sulfate anions can serve as a negative charge layer to repulse the chlorine anions away from the surface of electrodes, which improves about 5 times the stability.^[^ ^42^ ^]^ In addition, Dai et al. prepared NiFe‐LDH/NiS_x_ precursor.^[^ ^11^ ^]^ After anodic activation, sulfate and carbonate‐rich passivating layers were in‐situ formed on the surface of the precursor, which endows the electrocatalyst with chloride‐repelling characteristics, leading to the superior corrosion resistance in the seawater splitting. Therefore, in this work, the unbelievable durability of NiCoMoPO at high current densities for OER and HER in alkaline seawater can be attributed to the chlorine‐corrosion resistance of the PO_3_^−^ in NiCoMoPO by imposing a repulsive effect on Cl^−^ owing to the electrostatic repulsion.

### 2.4 Electrocatalytic Performance for Overall Water Splitting in Alkaline Freshwater and Seawater

Subsequently, NiCoMoPO served simultaneously as anodic and cathodic electrocatalysts of overall water splitting in alkaline freshwater and seawater. Figure 5d displays that NiCoMoPO needs low cell voltages of 1.850 and 2.096 V to afford 100 and 500 mA cm^−2^ in alkaline seawater respectively, while in basic freshwater, it requires lower cell voltages of 1.839 and 2.024 V to achieve the same current densities. Furthermore, in alkaline freshwater, to attain an ultra‐high current density of 1000 mA cm^−2^, it only requires a small cell voltage of 2.135 V, demonstrating the wonderful activity of overall water splitting at high current densities. Table S7 (Supporting Information) compares cell voltages of overall freshwater/seawater splitting at 100 and 500 mA cm^−2^ for NiCoMoPO and recently reported non‐noble electrocatalysts in alkaline electrolytes, demonstrating that NiCoMoPO's cell voltages are lower than those of most non‐noble electrocatalysts, which proves its tremendous activity for alkaline overall freshwater/seawater splitting. In addition, the Faradic efficiency (FE) of NiCoMoPO was tested via a facile drainage method (Figure S33, Supporting Information). Figure 5e displays the volumes of H_2_ and O_2_ as a function of the time, and the corresponding average volume‐ratio value (H_2_:O_2_) is ≈2.07, very close to the theoretical value of 2.0, signifying its high FE of nearly 100%.

Apart from the excellent activity, the durability of NiCoMoPO for overall freshwater/seawater splitting is incredibly surprising. First, in alkaline freshwater, its robust multi‐current process (Figure S34, Supporting Information) testifies to its outstanding mechanical stability. Furthermore, Figure S35 (Supporting Information) and Figure 5f show that NiCoMoPO can sustain nearly settled cell voltages for more than 200 and 300 h at high current densities of 500 and 1000 mA cm^−2^ respectively, surpassing almost all recently reported non‐noble electrocatalysts, as shown in Table S8 (Supporting Information), which reveals its salient stabilities at high current densities for alkaline overall freshwater splitting. Concerning alkaline overall seawater splitting (Figure 5g), the cell voltage of NiCoMoPO is almost unchanged for 1200 h at 500 mA cm^−2^, also implying superior durability for alkaline seawater splitting. Subsequently, the ClO^−^ content of the seawater electrolyte was detected by N, N‐diethyl‐p‐phenylenediamine (DPD) reagent. As shown in Figure S36 (Supporting Information), the post‐stability electrolyte is almost colorless and transparent, which is the same as the initial electrolyte, proving that no obvious ClO^−^ was produced in the long‐period durability for overall seawater splitting at the high current density of 500 mA cm^−2^. As comparisons, stabilities for overall seawater splitting of noble Pt/C/NF//RuO_2_/NF (−, +) were tested at 100, 300, and 500 mA cm^−2^ (Figures S37–S39, Supporting Information). It can only keep stable for a long time at the low current density of 100 mA cm^−2^, whereas it corroded rapidly at high current densities, especially at 500 mA cm^−2^, revealing that noble Pt/C/NF//RuO_2_/NF (−, +) has the inferior chlorine‐corrosion resistance in the seawater splitting process. In addition, the durability of NiCoMoO//NiCoMoO (−, +) was also performed to analyze the stability origin in the seawater splitting. Figure S40 (Supporting Information) shows that the cell voltage of NiCoMoO//NiCoMoO (−, +) increased quickly within ≈1 h, and it produced many ClO^−^ anions in the process of overall seawater splitting at 500 mA cm^−2^, manifesting its bad durability of overall seawater splitting at high current densities, which eliminates the effect of MoO_4_^2−^ via the electrostatic repulsion to enhance the chlorine‐corrosion resistance. The above result implies that the chlorine‐corrosion resistance of PO_3_^–^ in NiCoMoPO mainly contributes to its excellent stability for seawater splitting because of the electrostatic repulsion. Therefore, NiCoMoPO can hinder the CER and accelerate the OER in the overall seawater‐splitting process at high current densities. Table S9 (Supporting Information) compares stabilities at 500 mA cm^−2^ of NiCoMoPO and recently reported non‐noble electrocatalysts for OER, HER, and overall water splitting in alkaline seawater, demonstrating that the stabilities of NiCoMoPO exceed nearly all other compared electrocatalysts at the high current density in alkaline seawater. As a consequence, the incredibly superior durability of NiCoMoPO at high current densities (≥500 mA cm^−2^) implies its great potential for industrial application in both freshwater and seawater splitting.

### 2.5 Origin Analysis of the Excellent Activity and Stability for NiCoMoPO

**Figure 6:**
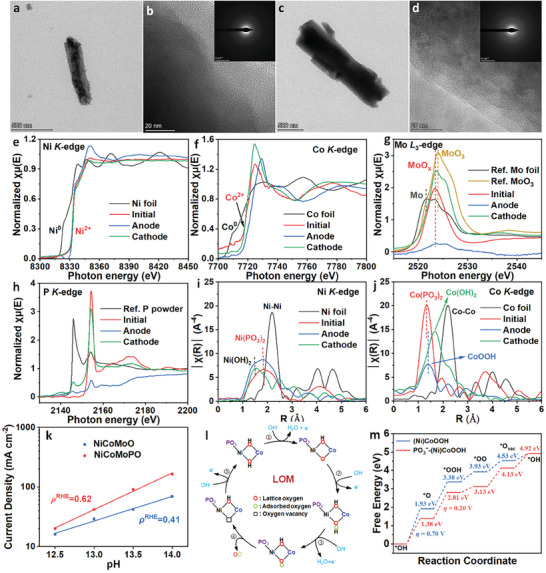
TEM images of the a,b) cathode and c,d) anode (The upper right inset is the corresponding SAED pattern). e) Ni *K*‐edge XANES spectra of reference Ni foil, initial NiCoMoPO, anode, and cathode. f) Co *K*‐edge XANES spectra of reference Co foil, initial NiCoMoPO, anode, and cathode. g) Mo *L*_3_‐edge XANES spectra of reference Mo foil, reference MoO_3_, initial NiCoMo, anode, and cathode. h) P *K*‐edge XANES spectra of reference P powder, initial NiCoMoPO, anode, and cathode. i) Ni *K*‐edge k^3^‐weighted FT‐EXAFS spectra of reference Ni foil, initial NiCoMoPO, anode, and cathode. j) Co *K*‐edge k^3^‐weighted FT‐EXAFS spectra of reference Co foil, initial NiCoMoPO, anode, and cathode. The cathode and anode are post‐NiCoMoPO electrocatalysts after the overall‐seawater‐splitting stability test. k) The logarithms of current density for NiCoMoO and NiCoMoPO at 1.60 V as a function of the pH value. l) Proposed OER mechanism of LOM. m) The calculated Gibbs free energy diagram of OER steps for the LOM pathway.

To study the origin of the excellent activity and stability for NiCoMoPO, the anodes and cathodes after the stability test of 300 h for overall seawater splitting were characterized by SEM, TEM, XRD, XPS, and XAFS. First, SEM images (Figure S41, Supporting Information) show that after the stability test of overall seawater splitting, the cathode and anode maintain almost the initial 3D‐nanowire morphology, indicating the excellent mechanical stability of the cathode and anode in the seawater splitting processes. Besides, SEM and the corresponding elemental mapping images of the cathode (Figure S42, Supporting Information) and anode (Figure S43, Supporting Information) after the overall‐seawater‐splitting stability test show the existence and homogeneous dispersion of Ni, Co, Mo, P, and O elements. Meanwhile, their EDS and the corresponding elemental content (Figures S44 and S45, Supporting Information) display that after the overall‐seawater‐splitting stability test, the Ni and Co contents of the cathode and anode are similar in atomic percent. However, compared to the cathode, the Mo and P contents of the anode considerably reduce, while its O content increases, signifying that the limited amount of MoO_x_ and metaphosphates in the anode are oxidized and transformed into some oxygen‐containing species appear at high anodic potential due to the oxidation, which is similar to the self‐reconstruction of some OER electrocatalysts, such as Pr_3_Ir_1−x_Mo_x_O_7_,^[^ ^43^ ^]^ NiMoO_4_,^[^ ^44^ ^]^ and CoFe(H_3_O)(PO_4_)_2_.^[^ ^45^ ^]^ Subsequently, TEM was applied to analyze the cathode and anode after the stability test. **Figure** **6**a,c shows their nanowire structure. Meanwhile, their HRTEM images (Figure 6b,d) and the corresponding SAED patterns (The upper right insets in Figure 6b,d) exhibit that all lattice fringes and corresponding electron diffusion spots disappear after the overall‐seawater‐splitting stability test, which proves that the crystalline NiCoMoPO in both cathode and anode were completely transformed into amorphous components in the overall‐seawater‐splitting process. In addition, Figures S46–S49 (Supporting Information) show HAADF‐TEM, the corresponding elemental mapping images, and the corresponding line‐scan EELS spectra of the cathode and anode. All elements in both the cathode and anode are uniformly dispersed in the corresponding nanowires. Moreover, the Co and P intensities of the anode are lower than those of the cathode, indicating the lower Co and P contents in the anode, which is consistent with the SEM‐EDS result.

Then, XRD was used to analyze the composites of the anode and cathode after the stability test of overall seawater splitting. No obvious diffusion peaks exist in the XRD patterns (Figure S50, Supporting Information) of the corresponding anode and cathode, manifesting their amorphous structures, in agreement with the TEM result. At last, XPS and XAFS were used to further analyze the composites, chemical valences, and geometric environments of the cathode and anode after the stability test of overall seawater splitting. The XPS spectra (Figures S51–S55, Supporting Information) show that after the overall‐seawater‐splitting stability test, the MoO_x_ and PO_3_^−^ in the cathodic NiCoMoPO remain, and the Ni(PO_3_)_2_ and Co(PO_3_)_2_ may become Ni(OH)_2_ and Co(OH)_2_ as active phases for HER, owing to the high cathodic potentials.^[^ ^15^, ^46^ ^]^ Regarding the Co 2p spectrum of the anodic NiCoMoPO (Figure S51, Supporting Information), a new peak of Co^3+^, related to CoOOH, appears, verifying that the Co(PO_3_)_2_ is oxidized into CoOOH as an active component for OER under high anodic potentials.^[^ ^45^ ^]^ Interestingly, in the Ni 2p spectrum of the anodic NiCoMoPO (Figure S52, Supporting Information), only the doublet peaks of Ni^2+^ are present, and no peak of NiOOH exists, which testifies that Ni(PO_3_)_2_ may be transformed into Ni(OH)_2_ as an active phase for OER under high anodic potentials. Moreover, the intensity of the Mo 3d spectrum for the anode is low (Figure S53, Supporting Information), implying the low content of MoO_x_ in the anodic NiCoMoPO, which reveals that under high anodic potentials, the MoO_x_ in NiCoMoPO was oxidized and dissolved in the alkaline seawater electrolyte. Regarding the P 2p spectra (Figure S54, Supporting Information), the main peaks of the anode and cathode are assigned to the PO_3_^−^, revealing that at high anodic and cathodic potentials, lots of PO_3_^−^ are preserved, which can be applied to impose a repulsive effect on Cl^−^ through the electrostatic repulsion, endowing the NiCoMoPO with excellent durability for overall seawater splitting. In the O 1s spectrum (Figure S55, Supporting Information), the peak of the anode, associated with MoO_x_, almost disappears, which accords with the result of the Mo 3d spectrum for the anode. Interestingly, for the anode, two new peaks, ascribed to OH and OOH,^[^ ^47^ ^]^ appear, while for the cathode, one new peak of OH is observed, which indicates further that at high anodic potentials, Ni(PO_3_)_2_ and Co(PO_3_)_2_ were transformed into Ni(OH)_2_ and CoOOH respectively; at high cathodic potentials, they became Ni(OH)_2_ and Co(OH)_2_ respectively. Furthermore, normalized Ni *K*‐edges XANES spectra (Figure 6e) show that the Ni valences of the cathodic and anodic NiCoMoPO sustain +2, revealing that Ni(PO_3_)_2_ is not easy to be oxidized and reduced under high anodic and cathodic potentials respectively, which is corresponding to the Ni 2p XPS spectra result (Figure S52, Supporting Information). Besides, Co *K*‐edges XANES spectra (Figure 6f) exhibit that the Co absorption edge position of the anode is greatly higher than those of the cathode and initial NiCoMoPO, indicating that the Co valence in the anode is much larger than +2, which reveals that the Co^3+^ related to CoOOH exists, corresponding to the Co 2p XPS spectra result of the anode (Figure S51, Supporting Information). For Mo *L*‐edge (Figure 6g; Figure S56, Supporting Information) and P *K*‐edge (Figure 6h) XANES spectra, Mo and P valences of the cathode and initial NiCoMoPO are the almost same, testifying to the excellent stability of the MoO_x_ and PO_3_^−^ in the cathode. However, the Mo and P intensities of the anode are lower, indicating its lower Mo and P contents in the anode, which is coincident with the Mo 3d (Figure S53, Supporting Information) and P 2p (Figure S54, Supporting Information) XPS spectra, as well as the EDS result (Figure S45, Supporting Information). Moreover, Figure 6i shows Ni *K*‐edge k^3^‐weighted FT‐EXAFS spectra of the anode, cathode, and initial NiCoMoPO, respectively. After the stability test, for the anode and cathode, the peak of Ni–O for Ni(PO_3_)_2_ remains, one new peak at ≈1.50 Å, belonging to Ni(OH)_2_, appears, verifying that part of Ni(PO_3_)_2_ was transformed into Ni(OH)_2_ at both high anodic and cathodic potentials. Concerning the Co *K*‐edge of the cathode (Figure 6j), the peak of Co–O for Co(PO_3_)_2_ disappears, and the peak of Co–O for Co(OH)_2_ can be observed, signifying that Co(PO_3_)_2_ was completely converted into Co(OH)_2_. For the anode, the peak of Co–O shifts positively to 1.41 Å, which can be ascribed to the existence of CoOOH. That is to say, at high anodic potentials, Co(PO_3_)_2_ was oxidized into CoOOH, which is consistent with the Co 2p XPS result (Figure S51, Supporting Information). Accordingly, in the process of overall seawater splitting at a high current density of 500 mA cm^−2^, NiCoMoPO can maintain its original 3D nanowire shape, signifying excellent mechanical stability. Meanwhile, under high cathodic and anodic potentials, the components of crystalline NiCoMoPO were completely transformed into amorphous phases. Under high cathodic potentials, Ni(OH)_2_/Co(OH)_2_ is the main active composite for HER. However, in the anode, the contents of MoO_x_ and PO_3_^−^ decrease, and new CoOOH and Ni(OH)_2_ components appear, signifying that Ni(OH)_2_/CoOOH is the OER main active phases. Concerning the origin of the outstanding durability for seawater splitting at high current densities, the PO_3_^−^ in both cathode and anode could contribute to the chloride‐repelling characteristics due to the electrostatic shield against anionic Cl^−^, leading to the high chlorine‐corrosion resistance for NiCoMoPO.

Due to the sluggish OER kinetics as the main bottleneck of water splitting, it is more important to investigate the OER mechanism of NiCoMoPO to disclose its excellent activity origin. First, LSV curves of as‐activated NiCoMoO and NiCoMoPO in different electrolytes with increasing pH values from 12.5 to 14.0 were obtained, as shown in Figure S57 (Supporting Information). Moreover, Figure 6k shows the logarithms of current density for NiCoMoO and NiCoMoPO at 1.60 V as a function of the pH value, which exhibits that OER activities of both NiCoMoO and NiCoMoPO have a strong pH dependence, signifying their non‐concerted proton‐electron transfer process.^[^ ^14^, ^48^ ^]^ Therefore, it is proposed that both NiCoMoO and NiCoMoPO follow the lattice oxygen oxidation mechanism (LOM) pathway. To get theoretical insights into the reaction mechanism, Density Functional Theory (DFT) calculations were used to determine the electronic structure and energy barrier. Based on the above experiment results, (Ni)CoOOH and PO_3_^−^‐(Ni)CoOOH were selected as models for DFT calculations because Ni(OH)_2_ can also be oxidized into NiOOH as the OER active phase at anodic potentials.^[^ ^49^ ^]^ Figures S58 and S59 (Supporting Information) demonstrate the optimized structures of (Ni)CoOOH and PO_3_^−^‐(Ni)CoOOH respectively. The proposed LOM pathway is displayed in Figure 6l, and the corresponding calculated Gibbs free energy diagram (Figure 6m) indicates that for the (Ni)CoOOH, the rate‐determining step (RDS) is the first step, namely, the formation of *O (*OH + OH^−^ → *O + H_2_O + e^−^), and the corresponding theoretical overpotential (*η*) is 0.70 V. However, coupled with PO_3_^−^, the RDS of PO_3_^−^‐(Ni)CoOOH becomes the formation of *OOH (*O + OH^−^ → *OOH + e^−^) and the theoretical *η* can be efficiently decreased from 0.70 V to 0.20 V. This verifies that the PO_3_^−^ can efficiently accelerate the OER process, leading to better OER kinetics for NiCoMoPO.

## 3 Conclusion

In this work, 3D Ni‐Co metaphosphates/molybdenum oxides heterostructure nanowires grown on NF were prepared via simple hydrothermal and phosphatization strategies. Owing to the optimized electronic structure, 3D nanowire structure, and chloride‐repelling characteristics, NiCoMoPO exhibits excellent activities and stabilities in both alkaline freshwater and seawater splitting. In addition, its OER mechanism determined by experiments and DFT calculations follows the LOM pathway, proving that PO_3_^−^ can significantly facilitate the OER process. Therefore, NiCoMoPO can drive 1000 mA cm^−2^ at low overpotentials of 467 and 442 mV for OER and HER respectively. Moreover, as a bifunctional electrocatalyst for overall freshwater splitting, it only requires a low cell voltage of 2.135 V to achieve 1000 mA cm^−2^ with robust stability of 300 h. Particularly, owing to the chloride‐repelling feature, to secure 500 mA cm^−2^ for alkaline seawater splitting, it only needs a low cell voltage of 2.096 V with incredible stability of 1200 h, which surpasses almost all recently reported non‐noble electrocatalysts. Hereby, this work provides an outstanding route for developing non‐noble electrocatalysts for freshwater/seawater splitting at high current densities.

## Conflict of Interest

The authors declare no conflict of interest.

## Supporting information



Supporting Information

## Data Availability

The data that support the findings of this study are available in the supplementary material of this article.
