# Supplementary material for: Bimetal Metaphosphate/Molybdenum Oxide Heterostructure Nanowires for Boosting Overall Freshwater/Seawater Splitting at High Current Densities
Source: Adv Sci (Weinh). 2024 Sep 30;11(44):2407892. doi: 10.1002/advs.202407892 (PMC11600247; doi:10.1002/advs.202407892)
Supplement: Supplementary file 1 — Supporting Information [file ADVS-11-2407892-s001.docx]

Supporting Information

Bimetal Metaphosphate/Molybdenum Oxide Heterostructure Nanowires for Boosting Overall Freshwater/Seawater Splitting at High Current Densities

Pan Wang^1,3^, Pai Wang^1^, Tongwei Wu^1^, Xuping Sun^1,2,^*, and Yanning Zhang^1,^*

^1^Institute of Fundamental and Frontier Sciences, University of Electronic Science and Technology of China, Chengdu, 610054, China

^2^College of Chemistry Chemical Engineering and Materials Science, Shandong Normal University, Jinan, 250014, China

^3^School of Materials and Energy, Guangdong University of Technology, Guangzhou, 510006, China

*Corresponding authors: Xuping Sun (E-mail: xpsun@uestc.edu.cn) and Yanning Zhang (E-mail: yanningz@uestc.edu.cn)

1. Experimental section

**1.1. Chemical reagents**

Ni(NO_3_)_2_·6H_2_O, Co(NO_3_)_2_·6H_2_O, Na_2_MoO_4_·2H_2_O, NaH_2_PO_2_·H_2_O, concentrated HCl, Pt/C (20 wt.%), RuO_2_ (99.95%), and Nafion dispersion (5% w/w in water and 1-propanol) were obtained from Alfa Aesar. Nickel foam (NF) was purchased from Taiyuan Lizhiyuan Battery Materials Co., Ltd. N, N-diethyl-p-phenylenediamine (DPD) reagent test kit was provided by Guangdong Huankai Microbial Technology Co., Ltd.

**1.2. Synthesis processes of electrocatalysts**

*1.2.1. Pretreatment of NF*

NFs were cut into pieces with a size of 2.0 × 3.5 cm. Then, they were washed ultrasonically by using ethanol, 3.0 M HCl, and deionized (DI) water, respectively.

*1.2.2. NiCoMo precursor*

0.291 g of Ni(NO_3_)_2_·6H_2_O, 0.291 g of Co(NO_3_)_2_·6H_2_O, and 0.484 g of Na_2_MoO_4_·H_2_O dissolved in 50 mL of H_2_O to form a clear solution. Subsequently, two pieces of NF were put in an autoclave with the above solution. After keeping at 160 °C for 6 h, NiCoMo precursor was grown on NF. Furthermore, NiCoMo precursors with other different molar ratios of Ni:Co (1:2, 2:1, 1:3, and 3:1) were prepared with the above same procedure, while the total molar amount of Ni and Co is always 2.0 mmol. In addition, NiCoMo precursor with more Mo was fabricated via the above process, apart from that Na_2_MoO_4_·H_2_O was changed to 3.5 mmol.

*1.2.3. Bimetal metaphosphates/molybdenum oxides (**Ni_x_Co_1−x_(PO_3_)_2_/MoO_x_, identified as “NiCoMoPO”)*

In a quartz tube furnace with an Ar gas flow, NiCoMo precursors were put in a porcelain boat at 350 °C, while NaH_2_PO_2_·H_2_O (1.0 g) was placed on the upstream side of the furnace. After 2 h, NiCoMoPO with different Ni/Co molar ratios and more Mo were fabricated. To optimize the best temperature, the phosphatization temperature was changed to 300 and 400 ℃.

*1.2.4.* *Bimetal molybdate (**Ni_x_Co_1−x_MoO_4_, identified as “NiCoMoO”)*

NiCoMo precursor was pyrolyzed in Ar flow at 350 ℃ for 2 h. After being rinsed and dried, NiCoMoO was achieved.

*1.2.5. Pt/C/NF and RuO_2_/NF*

5.0 mg of Pt/C was sufficiently dispersed in 300 μL of ethanol with 20 μL of Nafion dispersion via an ultrasound. Subsequently, it dripped uniformly on 1 cm^−2^ of NF surface area to prepare the Pt/C/NF electrode. Moreover, RuO_2_/NF has the same fabrication process as Pt/C/NF except that Pt/C was replaced by RuO_2_.

**1.3. Structural and Morphological Characterization**

X‐ray diffraction (XRD) was conducted on an X-ray diffractometer with Cu Kα radiation (λ = 1.54 Å) of Bruker D8. SEM was carried out on JEOL JSM-7600F with an elemental composition analysis by energy‐dispersive X‐ray spectroscopy (EDS). Transmission electron microscopy (TEM), high-angle annular dark-field scanning TEM (HAADF-STEM), the corresponding elemental mapping, line-scan electron energy loss spectroscopy (EELS), and the corresponding selected area electron diffraction (SAED) images were obtained on FEI Talos F200x, combined with EDS functionality. X‐ray photoelectron spectroscopy (XPS) measurements were conducted on Escalab 250Xi with an Al Kα radiation source. All the peaks were calibrated by referring to the C 1s peak of C–C at 284.8 eV. The X-ray absorption fine spectroscopy (XAFS) measurements were performed at the Shanghai synchrotron radiation facility (SSRF) and Beijing synchrotron radiation facility (BSRF).

**1.4. Electrochemical tests**

All tests were conducted on an Autolab PGSTAT302N/FRA workstation at room temperature. The three-electrode system was applied to measure HER and OER, where NF‐supporting electrocatalysts were directly applied as working electrodes, a carbon rod as the counter electrode, and a Hg/HgO electrode as the reference electrode. Meanwhile, a two-electrode system was used to test overall water splitting, where NF‐supporting electrocatalysts both directly served as the anode and cathode. Freshwater and seawater electrolytes are 1.0 M KOH and 0.5 M NaCl + 1.0 M KOH, respectively. All measured potentials vs. Hg/HgO electrode (*E*_Hg/HgO_) were calibrated to the potentials vs. reversible hydrogen electrode (*E*_RHE_), according to the equation (*E*_RHE_ = *E*_Hg/HgO_ + 0.924). All LSV curves were measured at a scan rate of 2 mV s^−1^. Electrochemical impedance spectroscopies were obtained at 1.53 V for OER and -0.2 V for HER in a frequency range of 10^5^–0.1 Hz with an amplitude of 10 mV. *C*_dl_ was derived from CV with different scan rates in non-faradic potential regions. Chronopotentiometry was used to evaluate the durability. For convenience to compare the activity with recently reported electrocatalysts, all potentials in this work were calibrated via iR compensation unless it was indicated individually.

**1.5. Turnover frequency (TOF) calculation^[1]^**

The TOF (s^−1^) can be estimated according to the below equations (1,2):

TOF (s^−1^) = I/2nF (HER) (1)

TOF (s^−1^) = I/4nF (OER) (2)

where I is the current density for samples during the LSV measurement in 1.0 M KOH, F is the Faraday constant (C/mol), and n is the number of active sites (mol) for different samples. The number of active sites (n) was measured from CV curves within the potential range from -0.2 to 0.6 V vs. RHE at a scan rate of 50 mV/s in 1.0 M PBS (pH=7). The n (mol) could be determined with the following equations (3,4):

n (mol) = Q/2F (HER) (3)

n (mol) = Q/4F (OER) (4)

**1.6. First-principle calculation and model details**

Density Functional Theory (DFT) calculations were carried out using the Vienna Ab-initio Simulation Package (VASP).^[2]^ The Perdew-Burke-Ernzerhof (PBE) functional within the generalized gradient approximation (GGA) was used to describe the exchange-correlation interactions.^[3]^ The electron-ion interactions were described by projector-augmented wave (PAW) potentials. The Hubbard-U terms for Co and Ni were considered, with the effective U value of 3.52 eV on the Co ions^[4]^ and 5.5 eV on the Ni ions.^[5]^ The DFT-D3 method was used to evaluate the van der Waals (vdW) correction.^[6]^ The energy cutoff for the plane wave-basis expansion was set to 500 eV. The force and energy convergence criteria were set as 0.02 eV Å^−1^ and 10^−5^ eV. The CoOOH (10$\overline{1}$4) plane has been identified as the potential active phase of metal oxyhydroxide.^[7]^ Five-layer CoOOH (10$\overline{1}$4) surfaces with or without PO_3_^−^ modification were used for simulation, where the bottom two layers were fixed while the other three layers fully relaxed. Moreover, all layers were separated by 15 Å of vacuum to avoid the interaction between the slab and its period images. The Brillouin zone integration was performed using 4×2×1 k-point sampling for all systems.

In alkaline conditions, the Gibbs free energy changes for the water oxidation steps using the LOM mechanism were calculated using the following equations (5–9) of four steps:

*OH + OH^−^ → *O + H_2_O + e^−^ (5)

*O + OH^−^ → *OOH + e^−^ (6)

*OOH + OH^−^→ *OO + H_2_O + e^−^ (7)

*OO → * + O_2_ (8)

* + OH^−^ → *OH + e^−^ (9)

where “*” represents the vacancy sites.

The free energies of the reaction intermediates were defined as by Δ*G* = Δ*E* + Δ*ZPE* – TΔ*S*, where Δ*E*, Δ*ZPE*, *T*, and Δ*S* represent the reaction energy, zero-point energy, temperature (298.15 K), and the entropy, respectively.


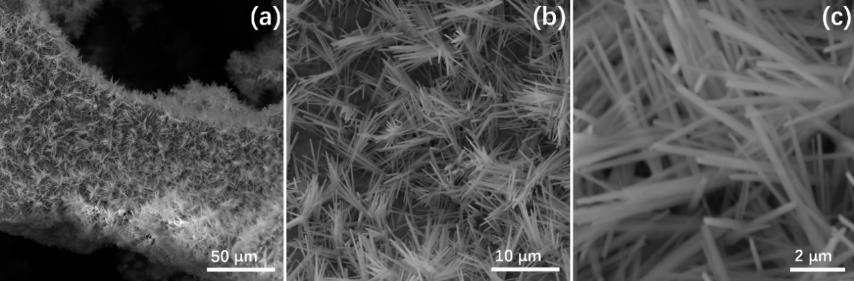


**Figure S1. (**a–c) SEM images of NiCoMo precursor.


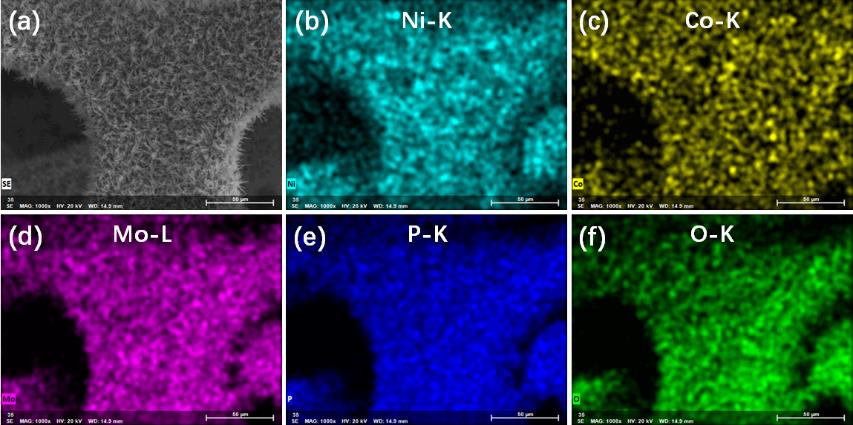


**Figure S2.** SEM and corresponding EDS elemental mapping images of NiCoMoPO.


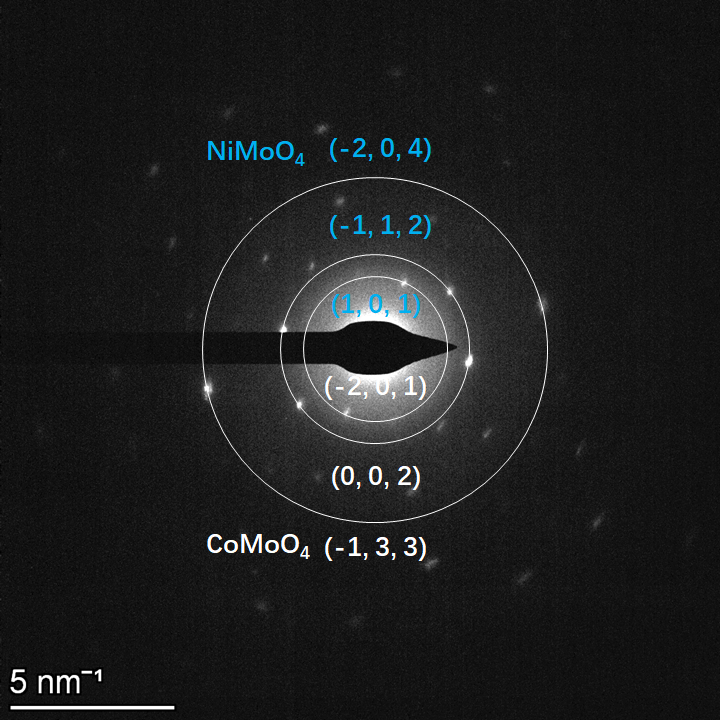


**Figure S3.** SAED pattern of NiCoMoO.





**Figure S4.** SAED pattern of NiCoMoPO obtained in the left marked area of Figure 2h via a Fast Fourier Transform.


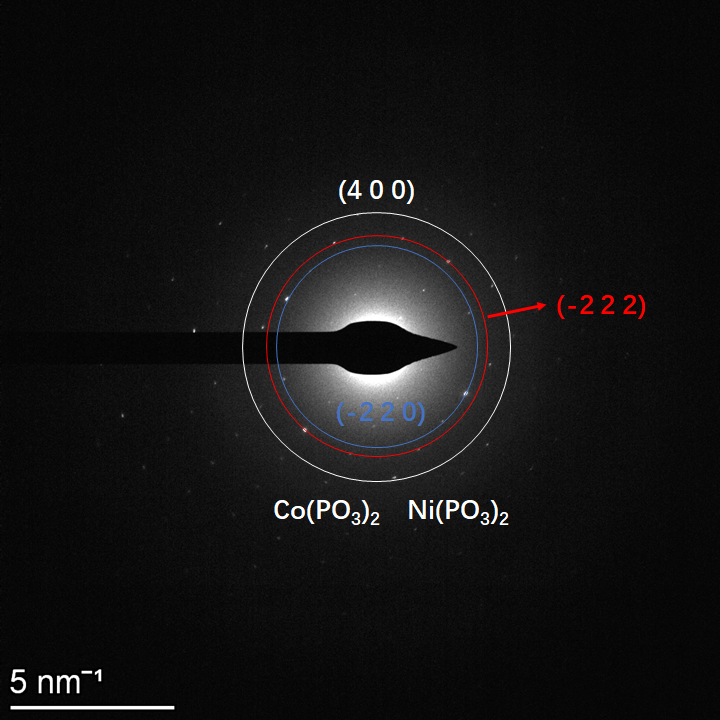


**Figure S5.** SAED pattern of NiCoMoPO.


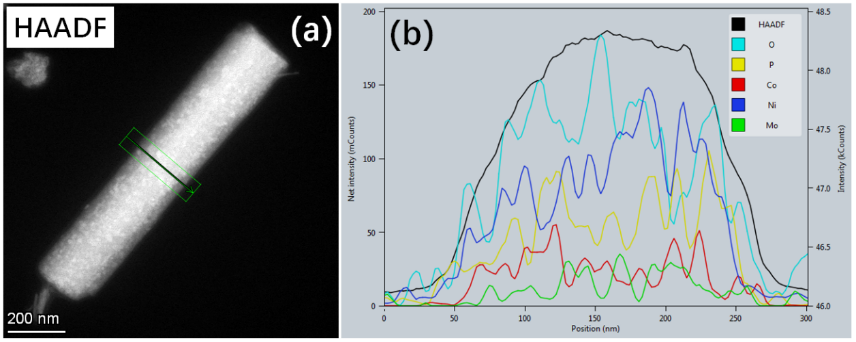


**Figure S6.** (a) HAADF-STEM image and b) the corresponding elemental profiles of line-scan EELS of NiCoMoPO.





**Figure S7.** XRD patterns of NiCoMo precursor, NiCoMoO, and NiCoMoPO (* indicates characteristic peaks of NF).





**Figure S8.** XPS survey spectra of NiCoMoPO and NiCoMoO.


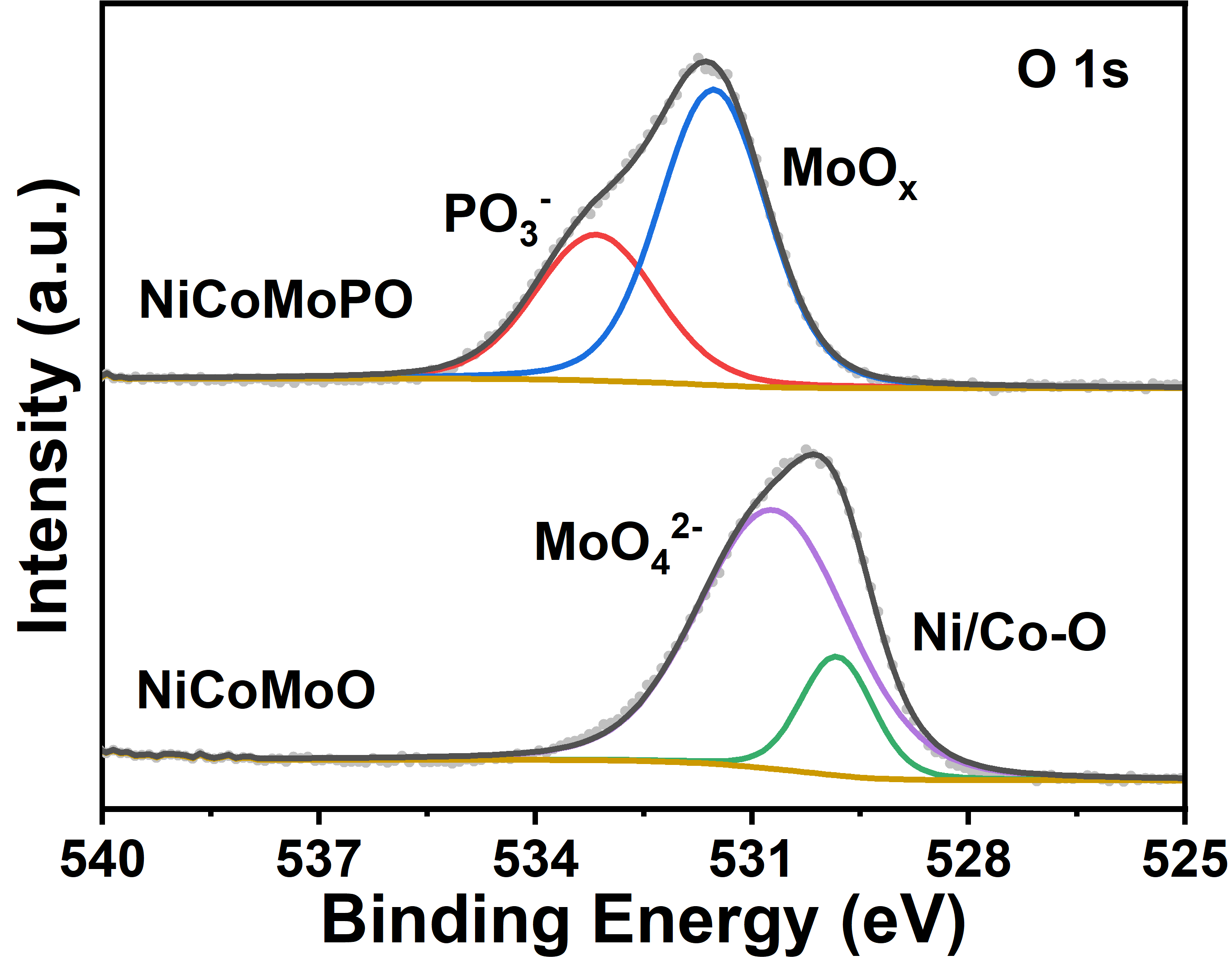


**Figure S9.** XPS spectra of O 1s for NiCoMoO and NiCoMoPO.


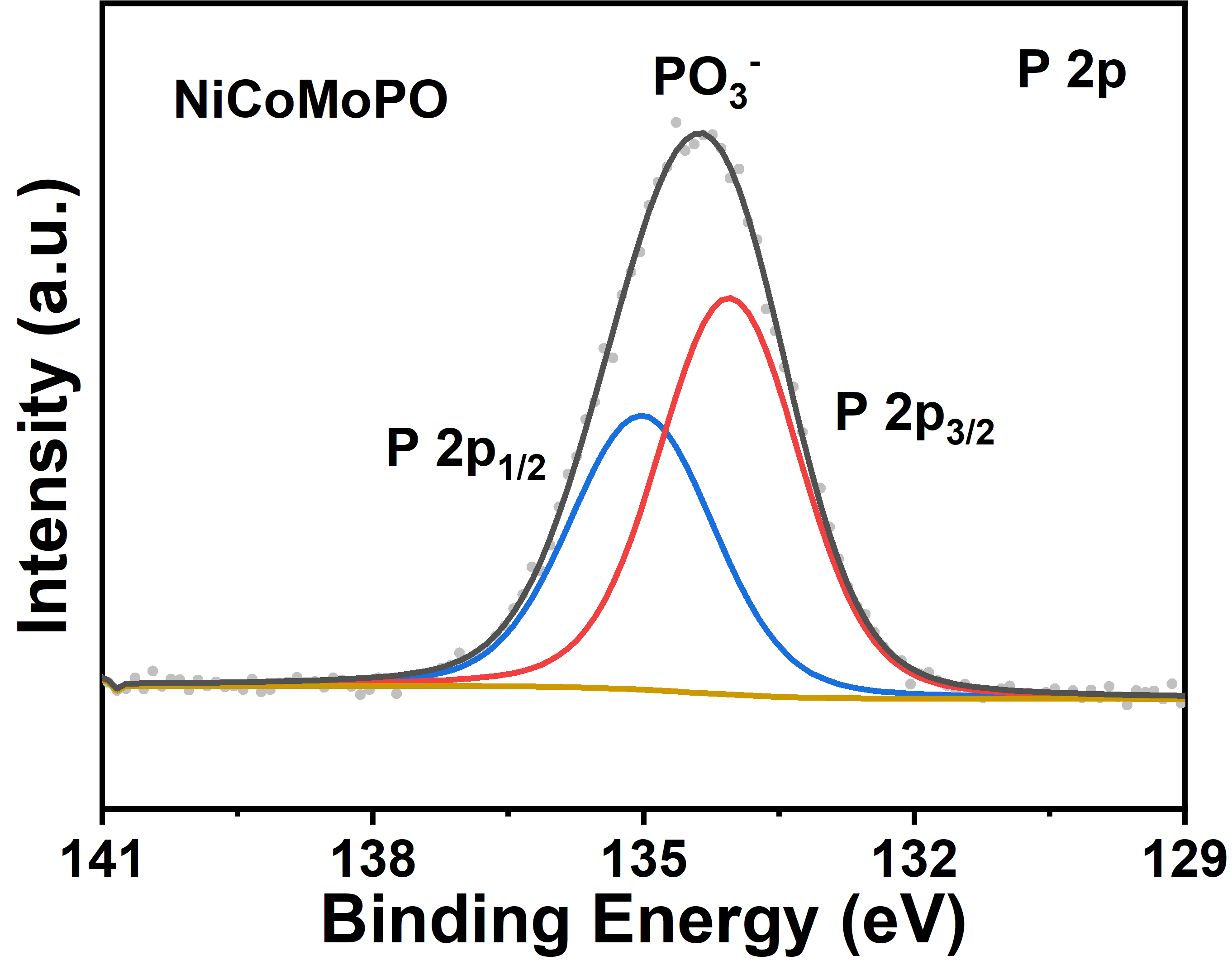


**Figure S10.** XPS spectra of P 2p for NiCoMoO and NiCoMoPO.


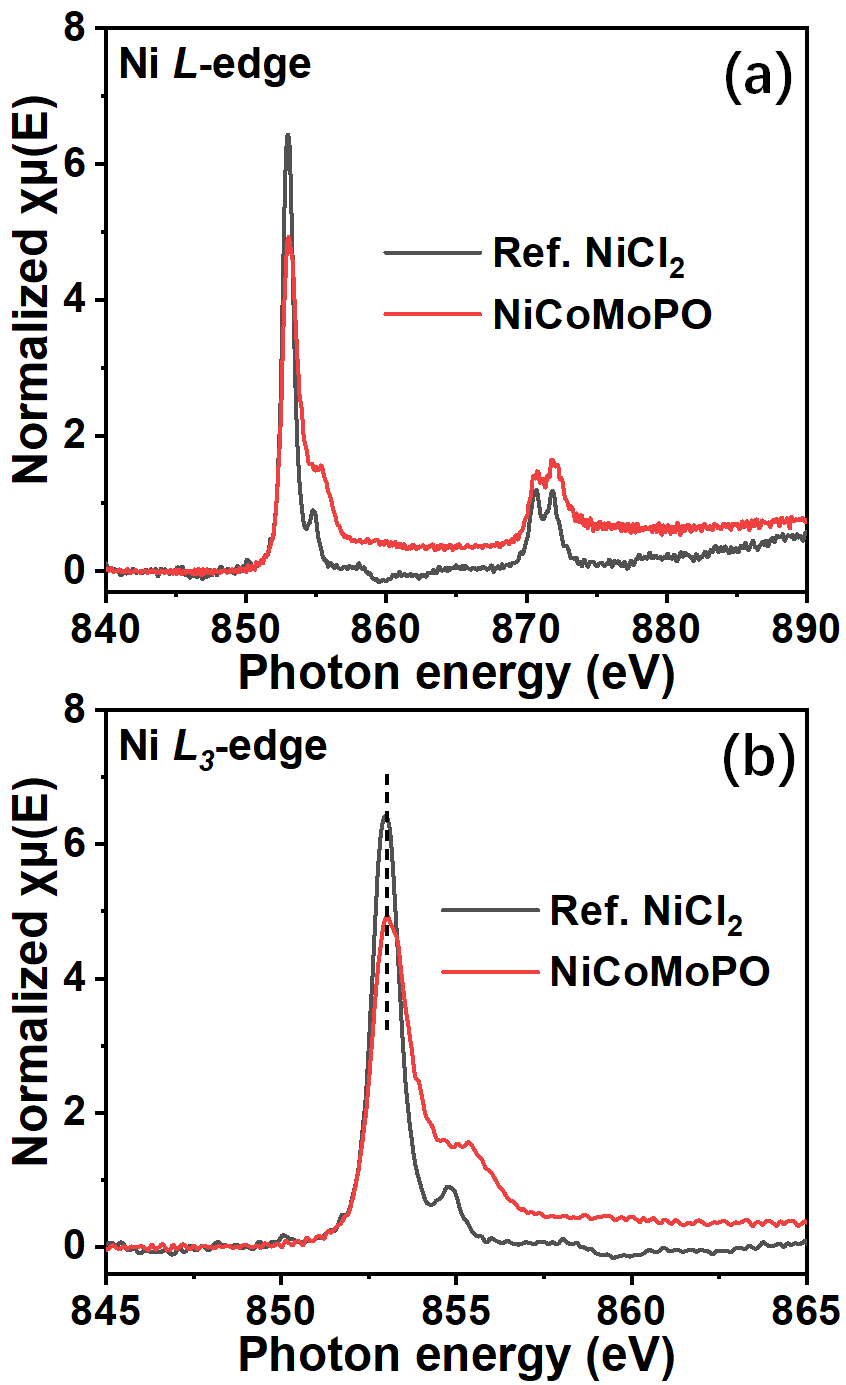


**Figure S11.** Normalized Ni (a) *L*-edge and (b) *L*_3_-edge XANES spectra of reference NiCl_2_ and NiCoMoPO.


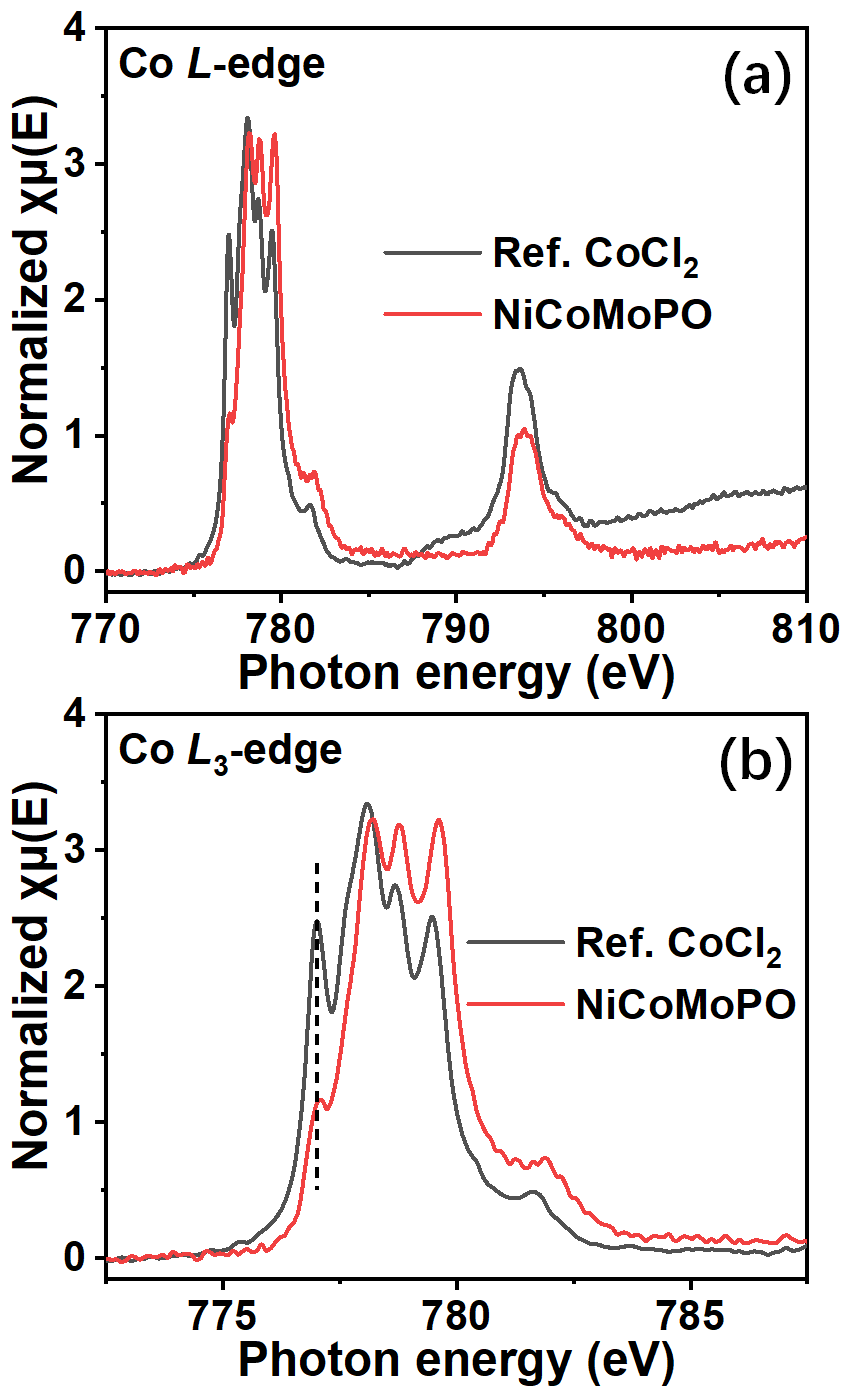


**Figure S12.** Normalized Co (a) *L*-edge and (b) *L*_3_-edge XANES spectra of reference CoCl_2_ and NiCoMoPO.


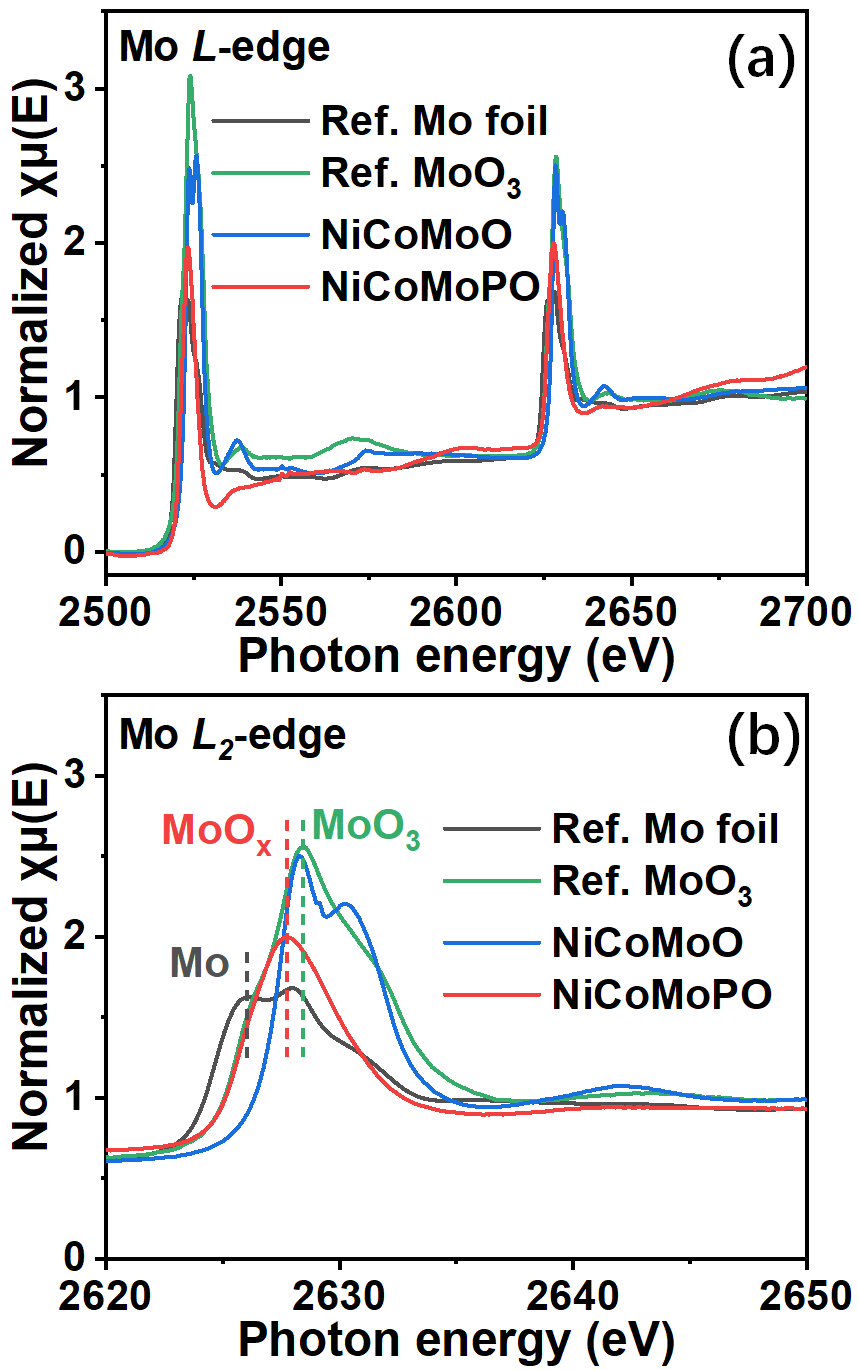


**Figure S13.** Normalized Mo (a) *L*-edge and (b) *L*_2_-edge XANES spectra of reference Mo foil, reference MoO_3_, NiCoMoO, and NiCoMoPO.





**Figure S14.** OER LSV curves of NiCoMoPO with Ni/Co of 1:1 at different temperatures of phosphatization.





**Figure S15.** OER LSV curves of NiCoMoPO with Ni/Co of 1:1 and NiCoMoPO with more Mo.





**Figure S16.** OER LSV curves of NiCoMoPO at different molar ratios of Ni/Co.


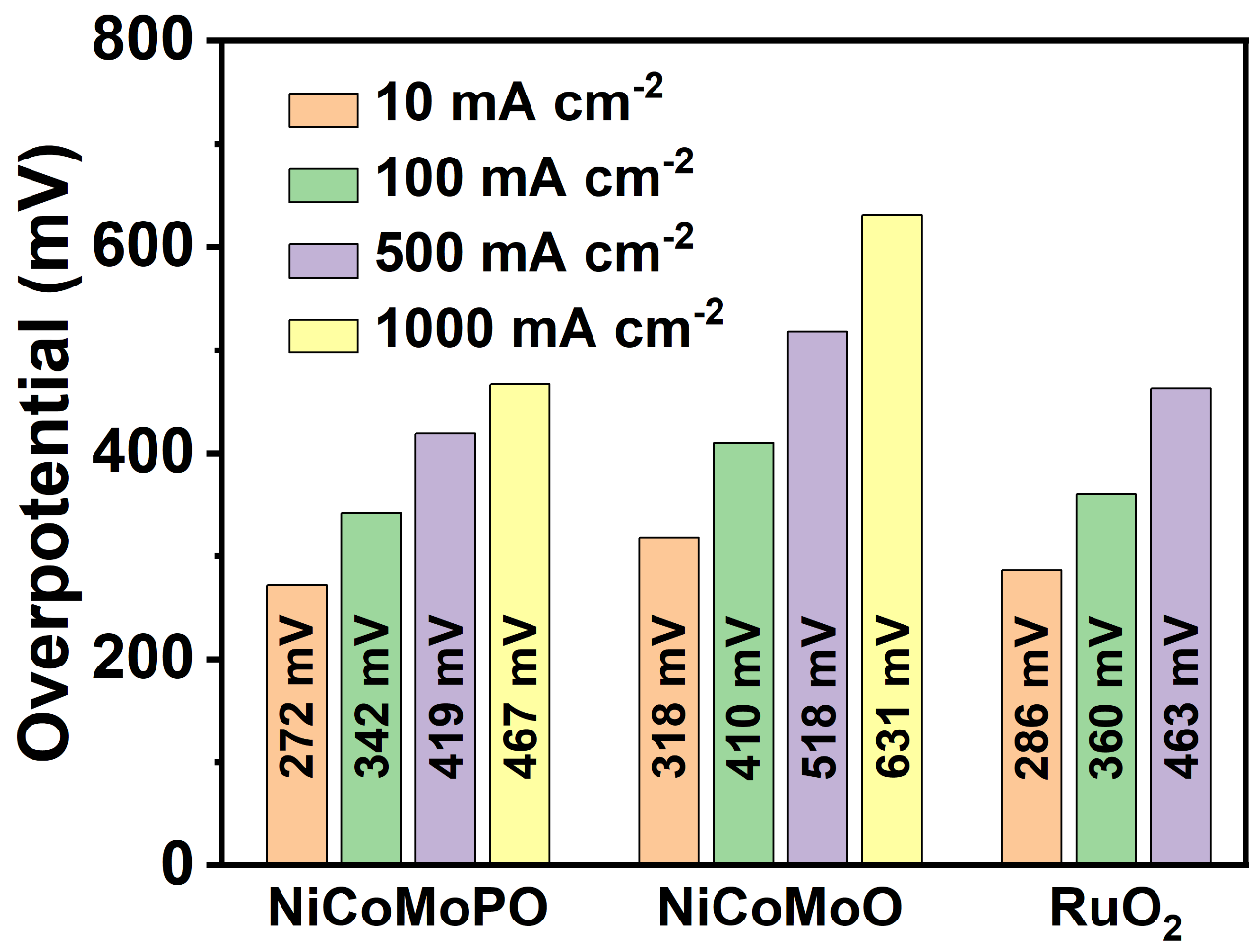


**Figure S17.** Comparison of overpotentials at different current densities for NiCoMoO, NiCoMoPO, and RuO_2_/NF.


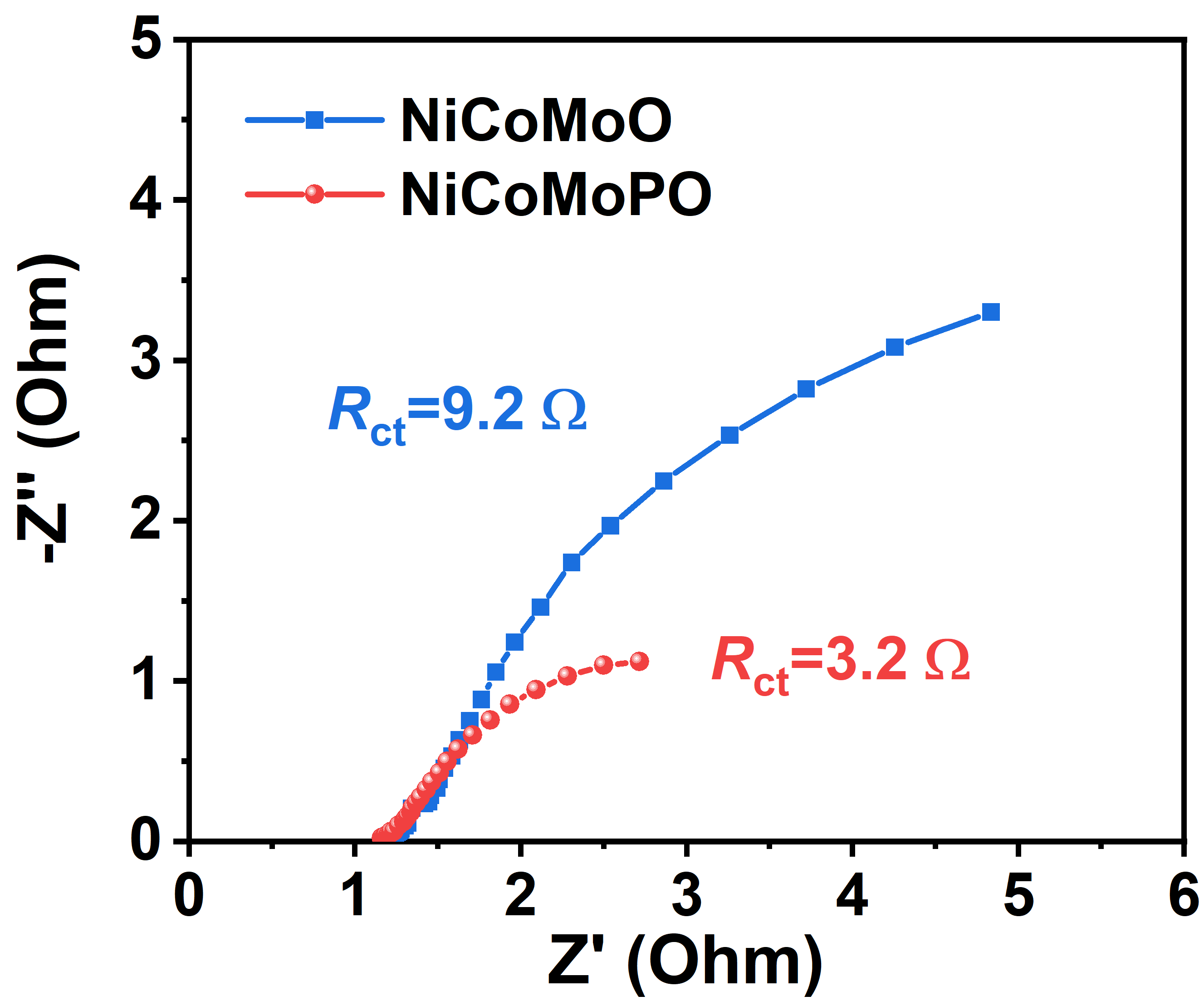


**Figure S18.** Nyquist plots of as-prepared samples.


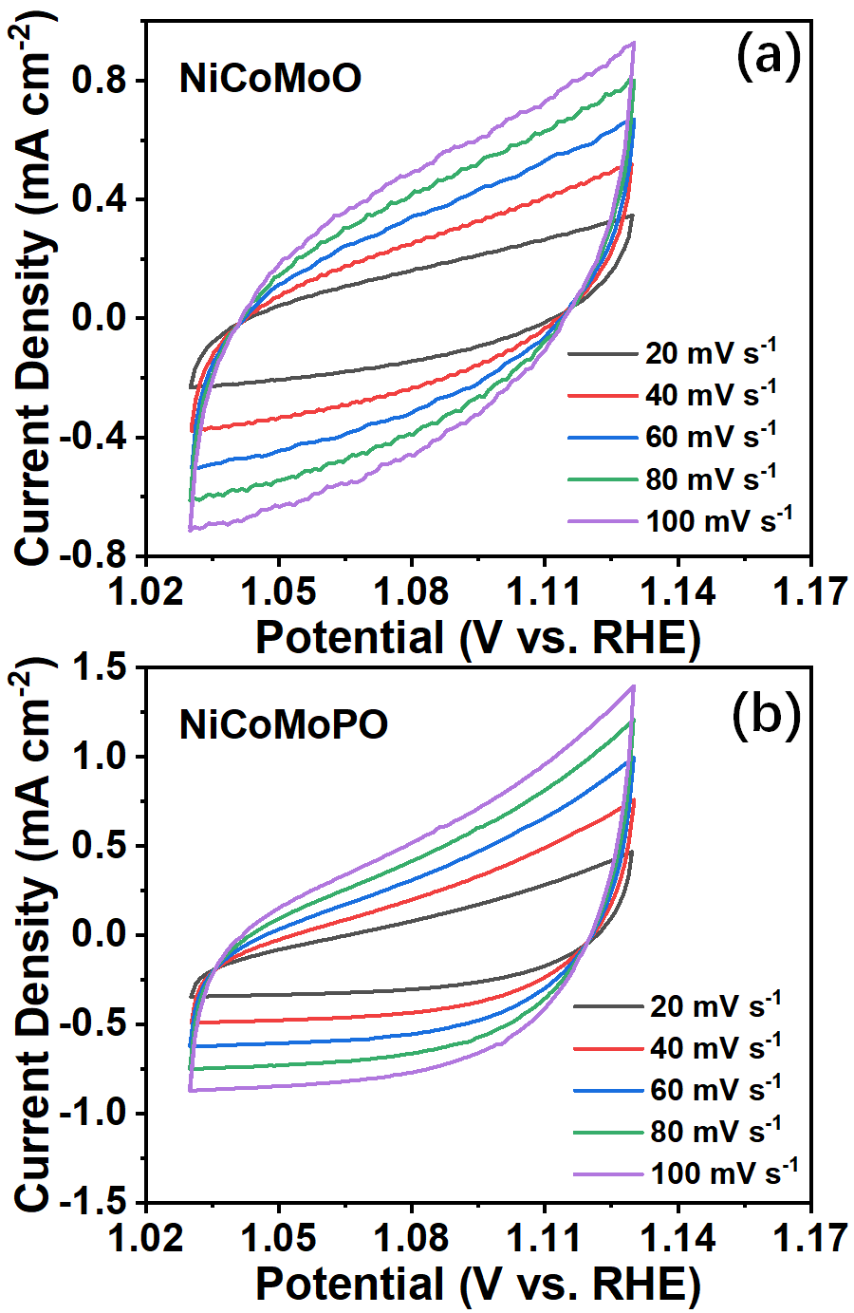


**Figure S19.** CV curves of (a) NiCoMoO and (b) NiCoMoPO at different scan rates.


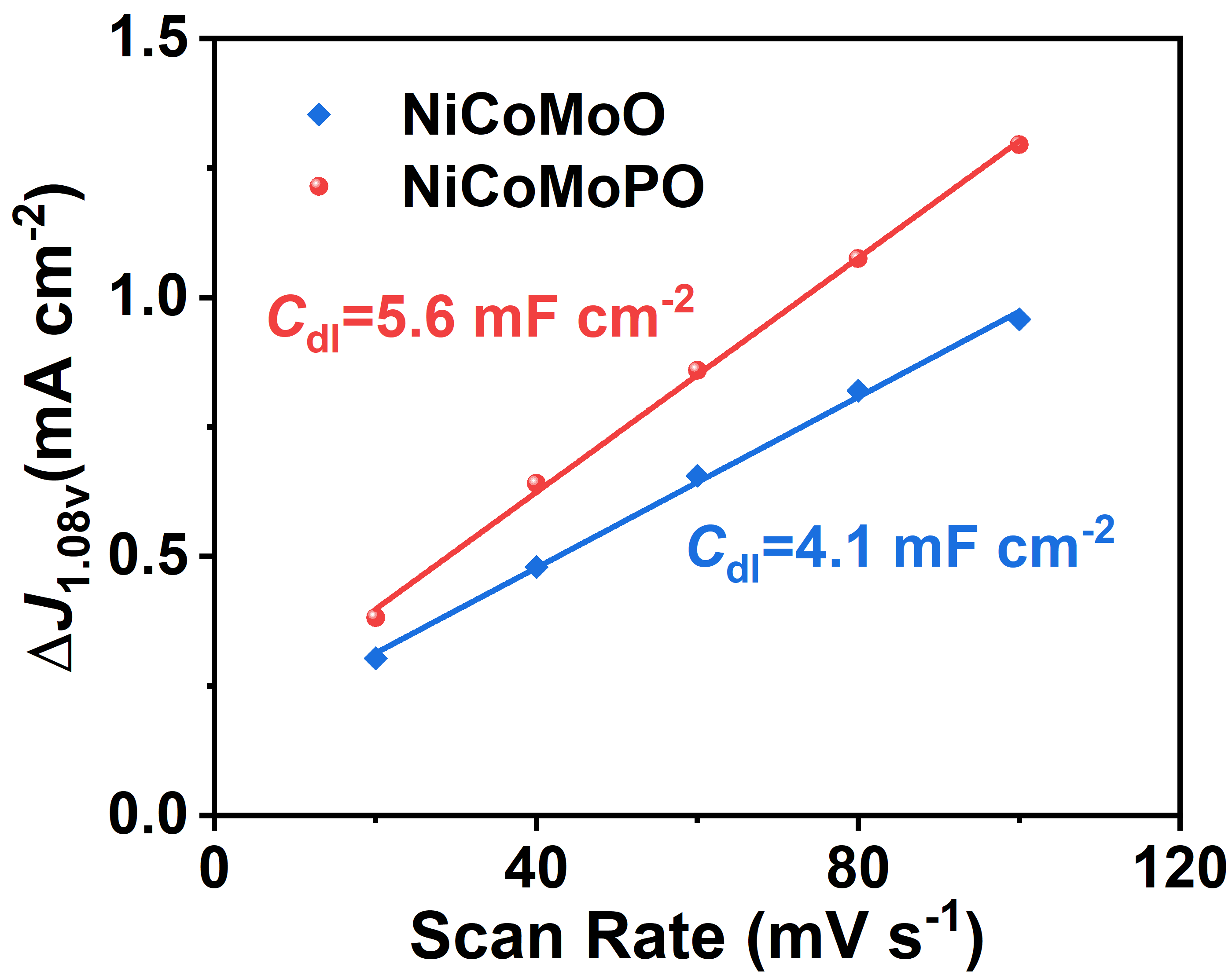


**Figure S20.** *C*_dl_ plots for NiCoMoO and NiCoMoPO.





**Figure S21.** CV curves of NiCoMoO and NiCoMoPO.





**Figure S22.** HER LSV curves of NiCoMoPO at different molar ratios of Ni/Co.


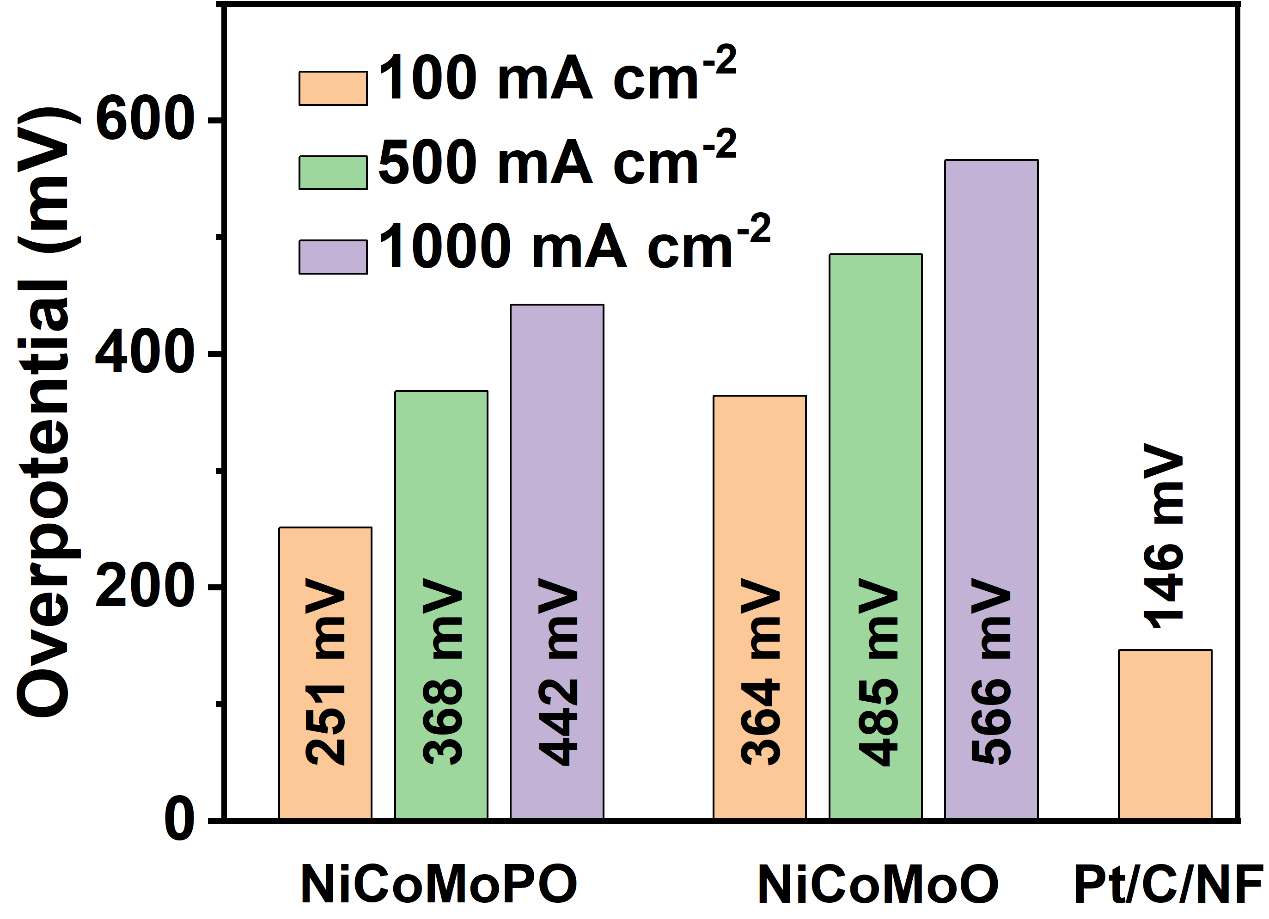


**Figure S23.** Comparison of overpotentials at different HER current densities for NiCoMoO, NiCoMoPO, and Pt/C/NF.


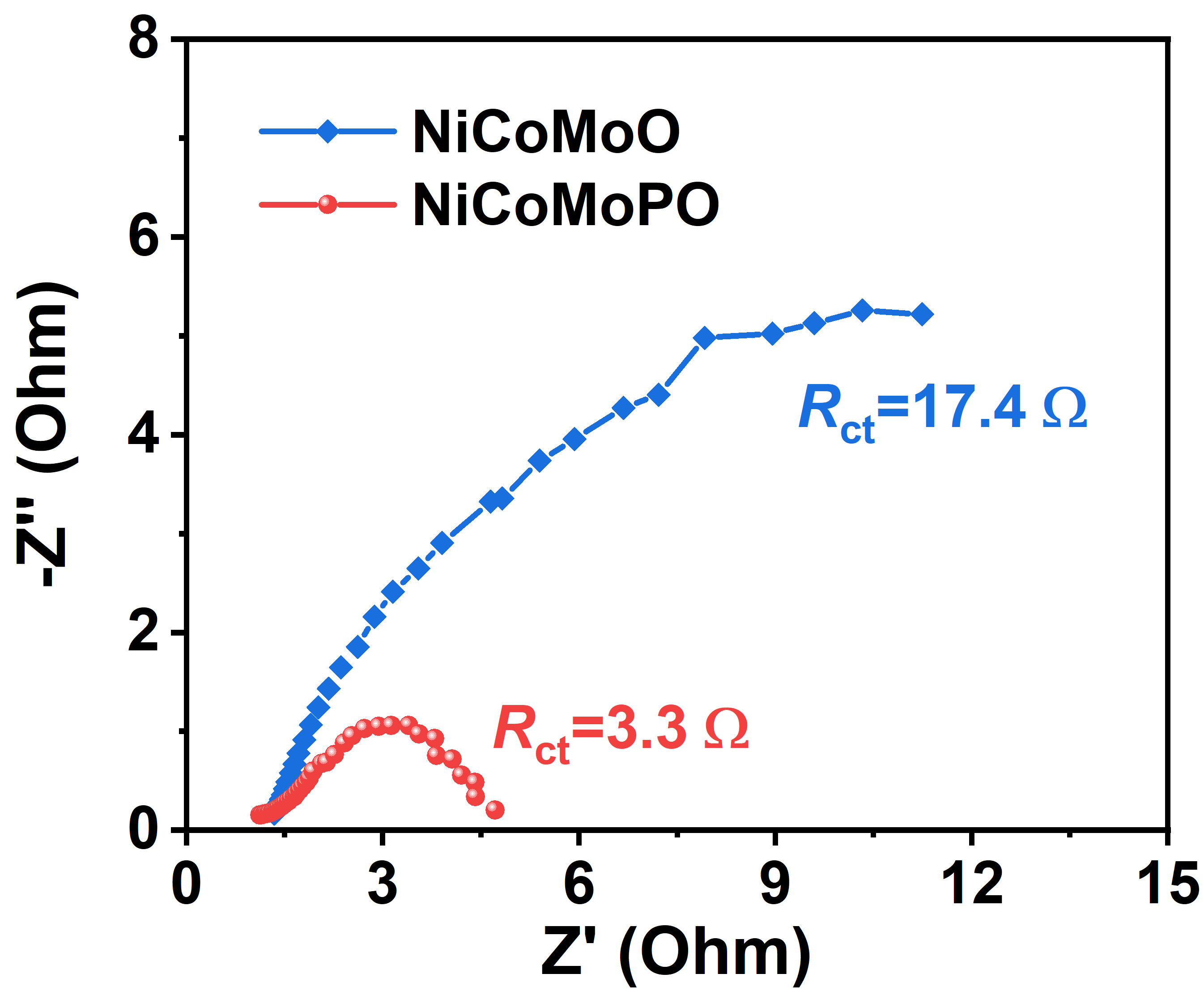


**Figure S24.** Nyquist plots of as-fabricated samples.


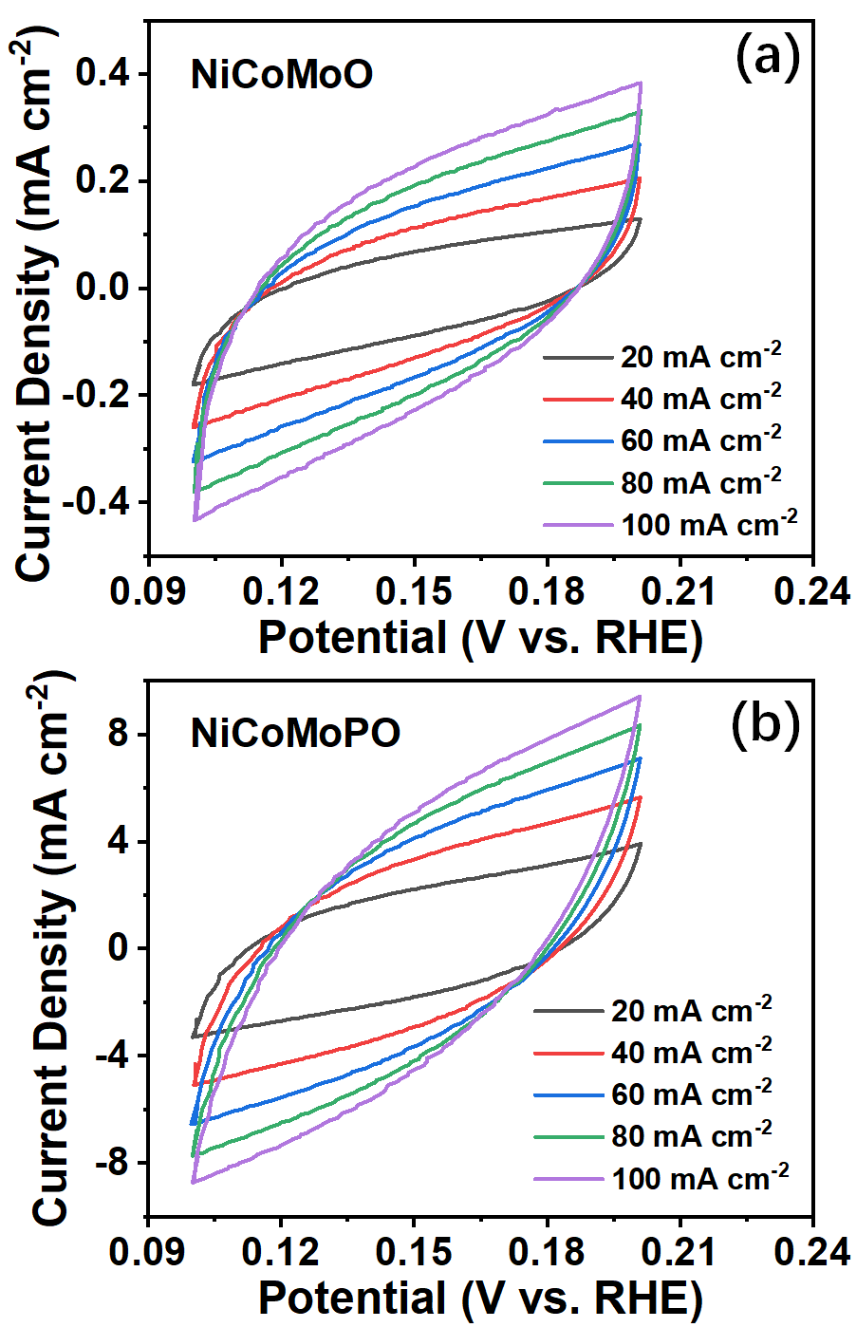


**Figure S25.** CV curves of (a) NiCoMoO and (b) NiCoMoPO at different scan rates.


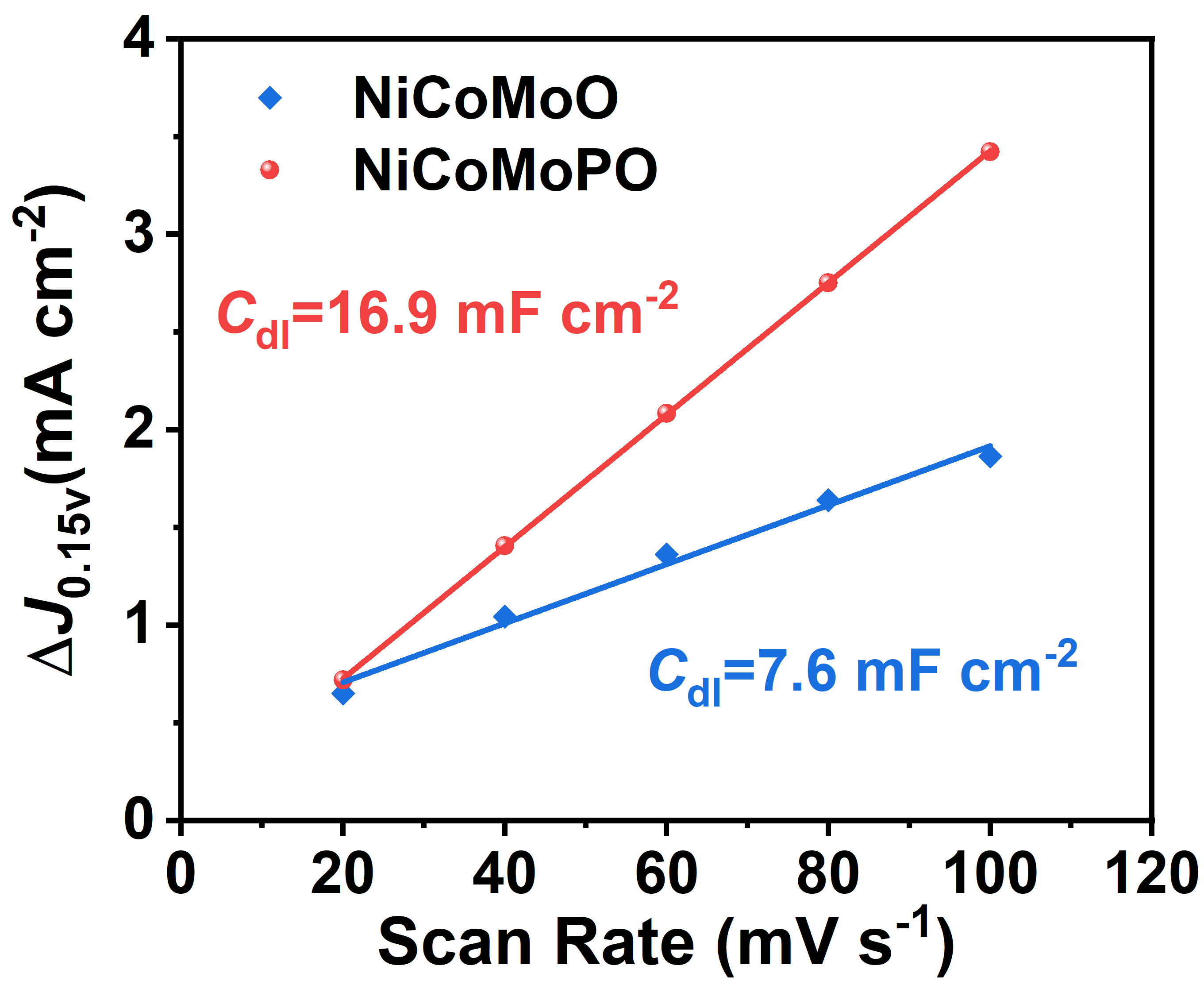


**Figure S26.** *C*_dl_ plots for NiCoMoO and NiCoMoPO.


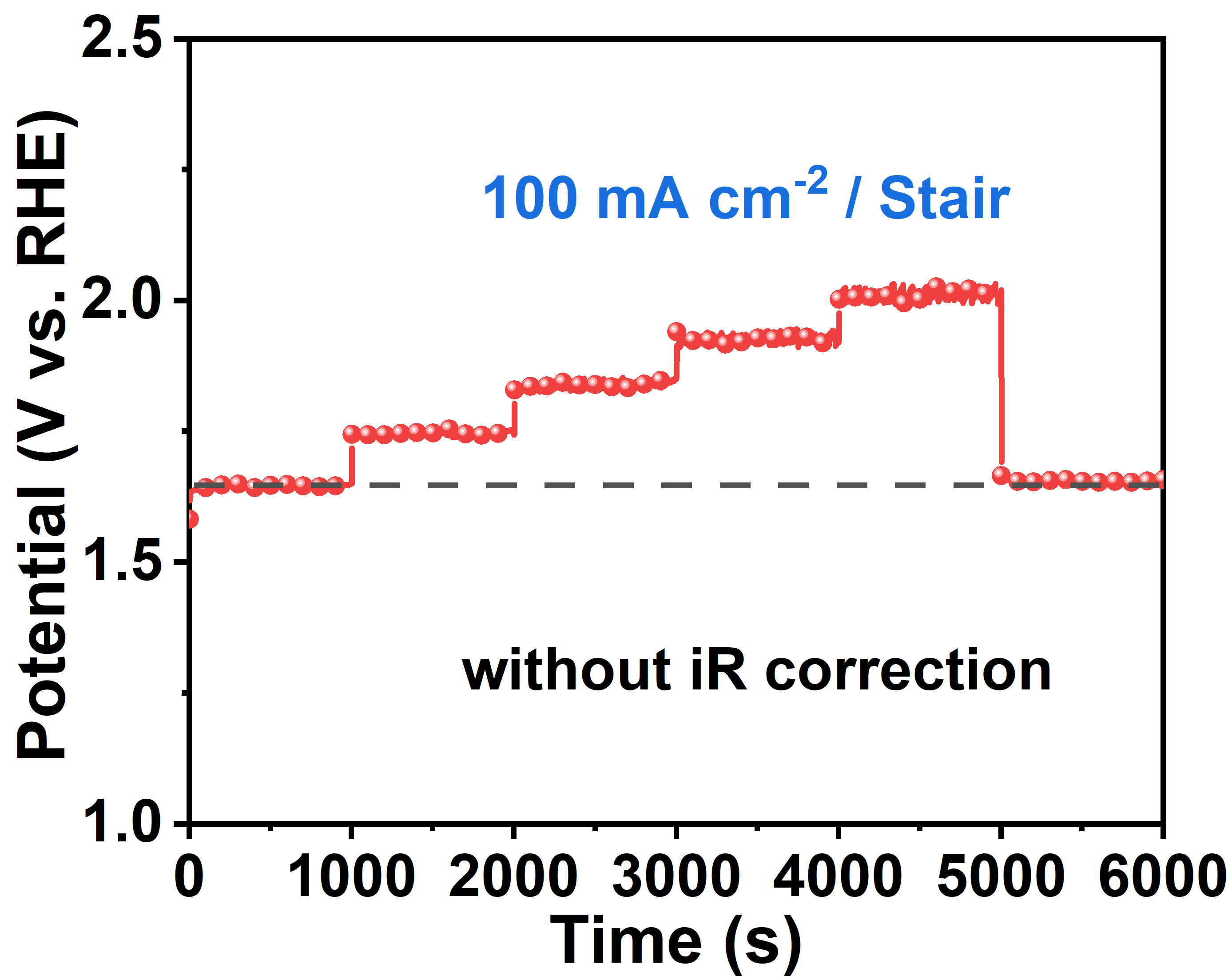


**Figure S27.** Multi-current OER process of NiCoMoPO without iR correction via a stair of 100 mA cm^−2^.


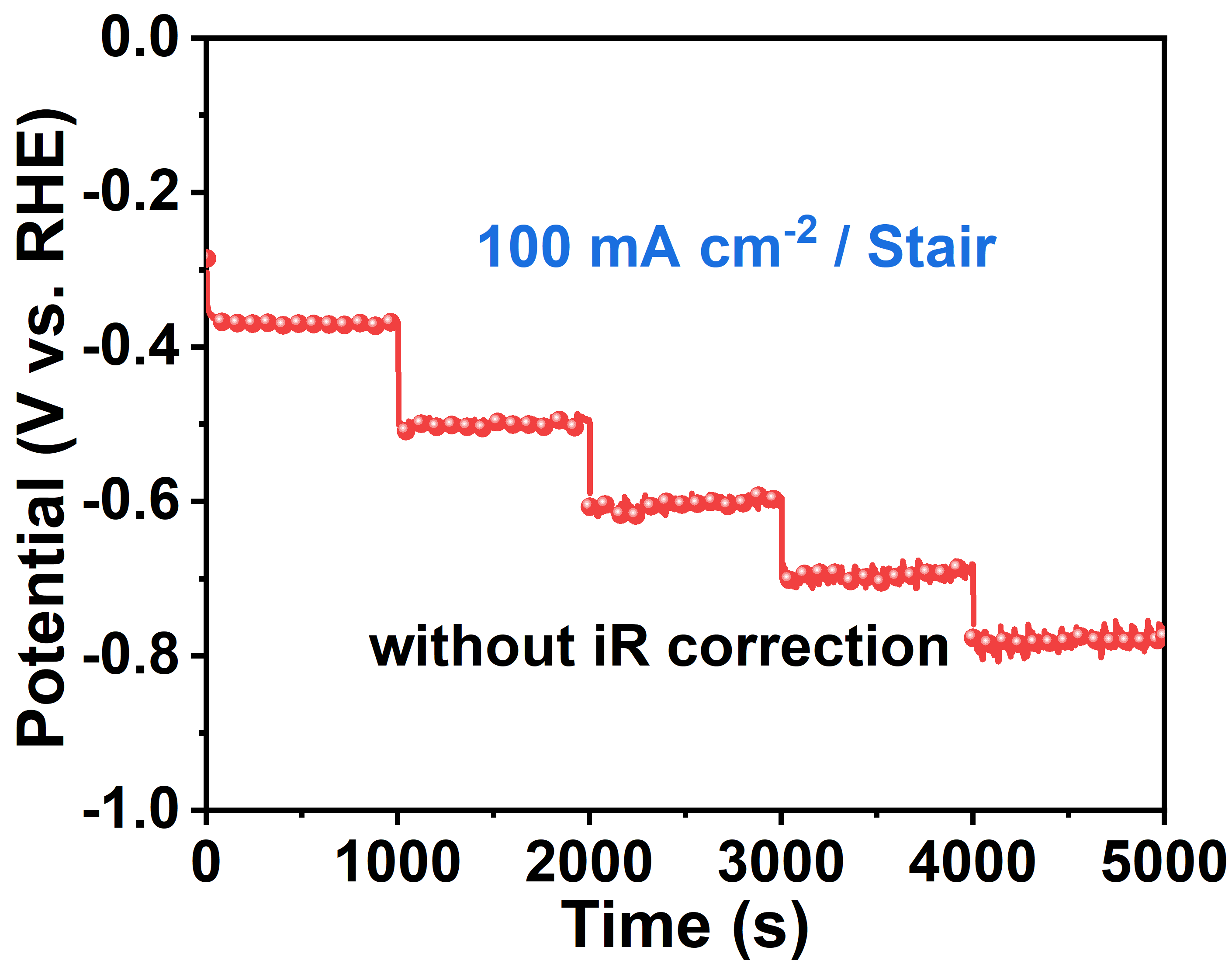


**Figure S28.** Multi-current HER process of NiCoMoPO without iR correction via a stair of 100 mA cm^−2^.


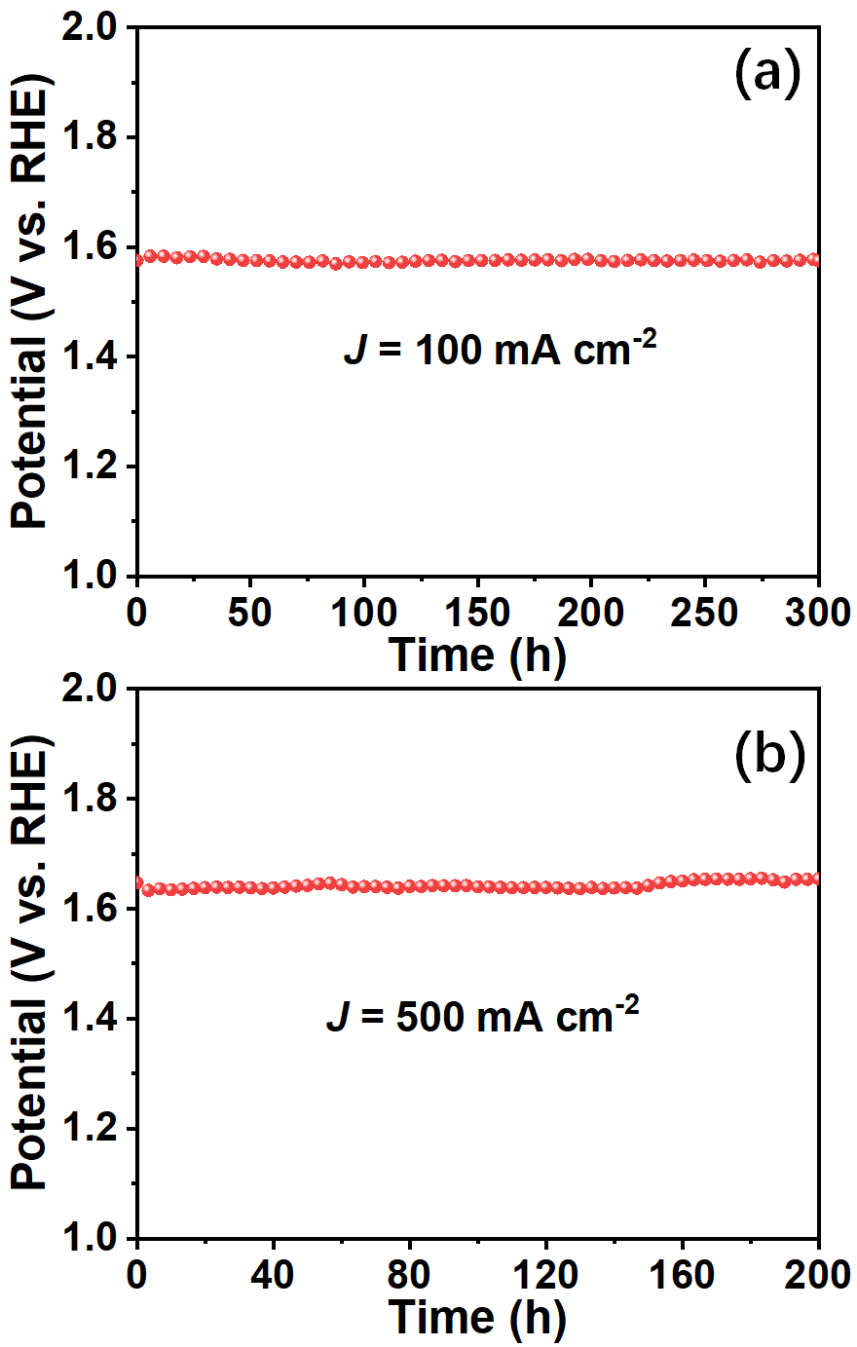


**Figure S29.** Chronopotentiometry curve of NiCoMoPO at (a) 100 and (b) 500 mA cm^−2^ for OER.





**Figure S30.** LSV curves of NiCoMoPO before and after chronopotentiometry test at 100 mA cm^−2^.


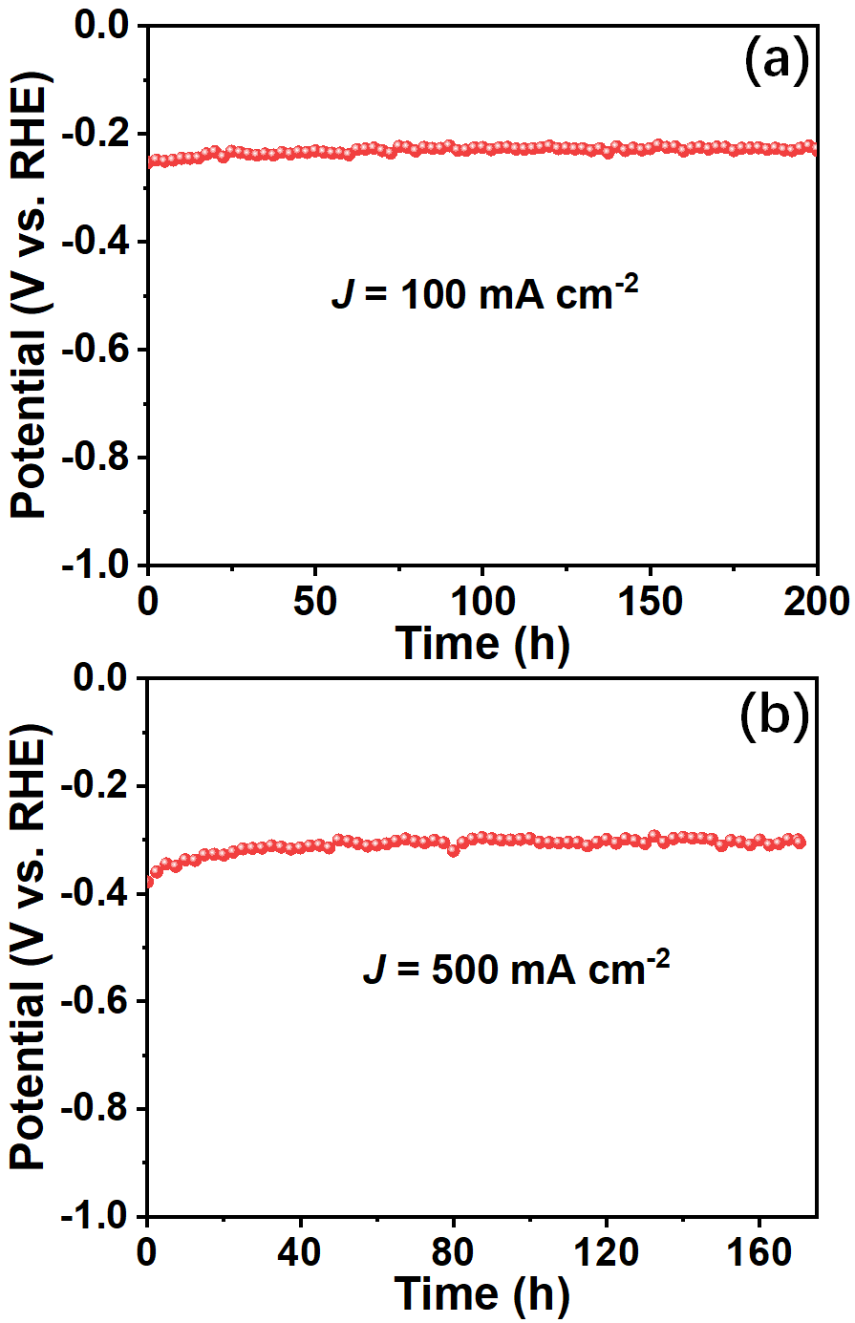


**Figure S31.** Chronopotentiometry curve of NiCoMoPO at (a) 100 and (b) 500 mA cm^−2^ for HER in freshwater.


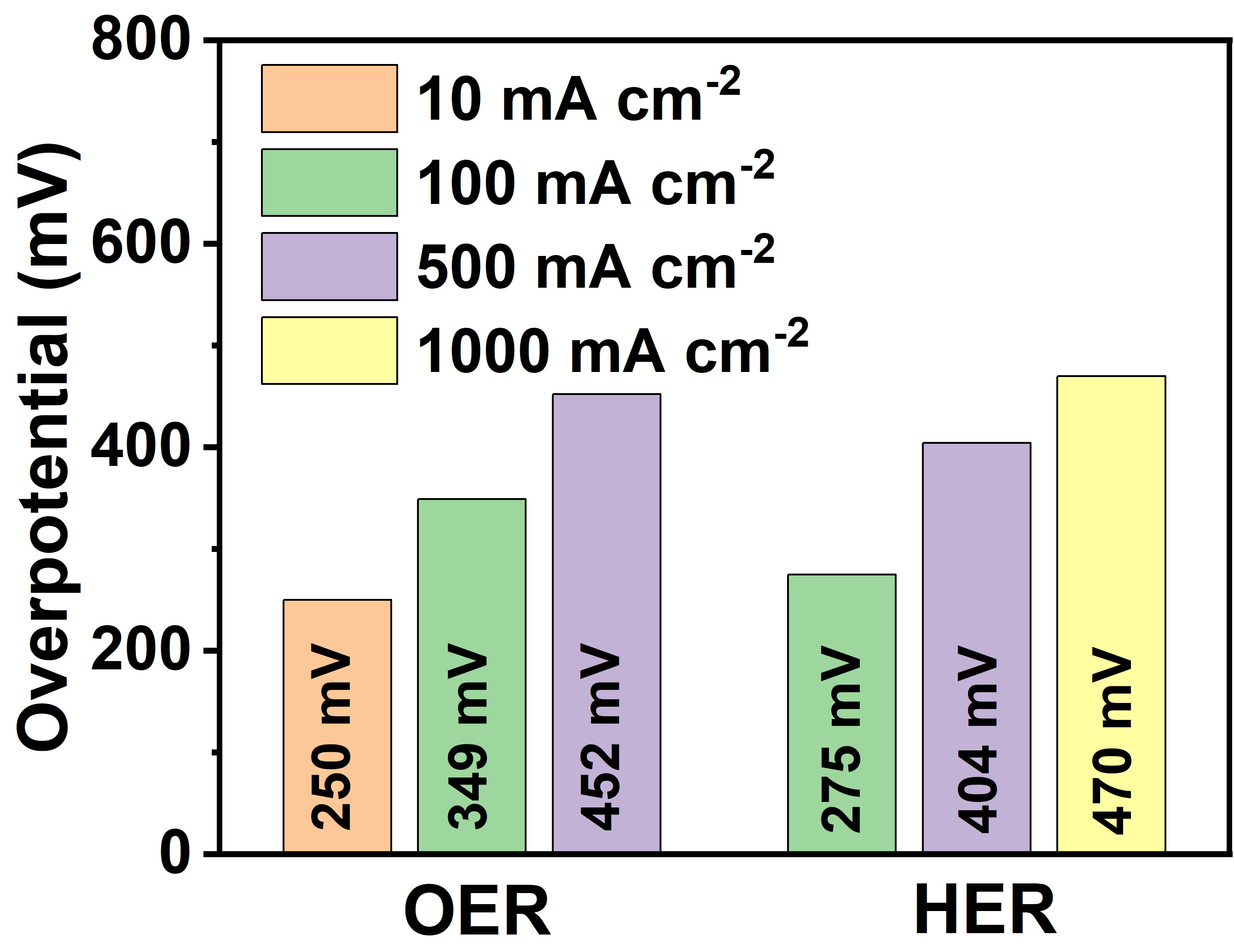


**Figure S32.** Overpotentials of NiCoMoPO for OER and HER at different current densities in alkaline seawater.


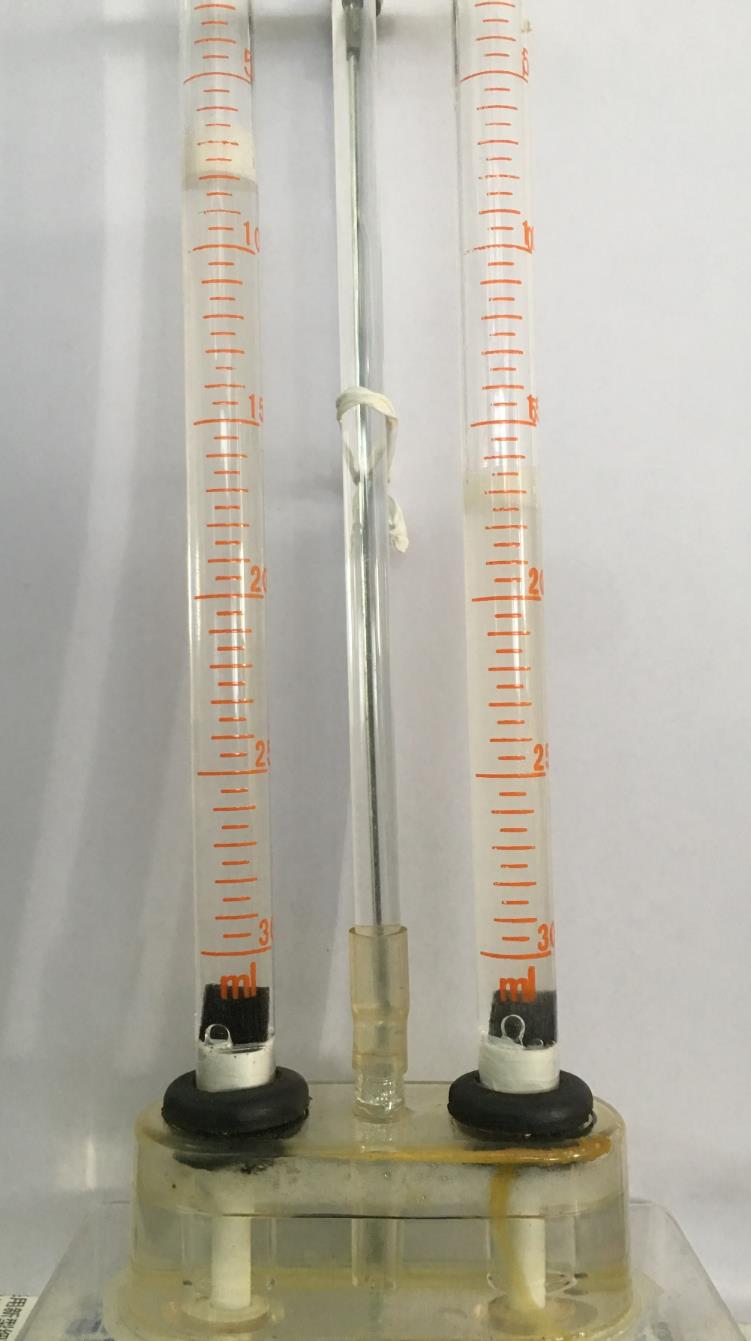


**Figure S33.** Image of FE measurement via a drainage method.


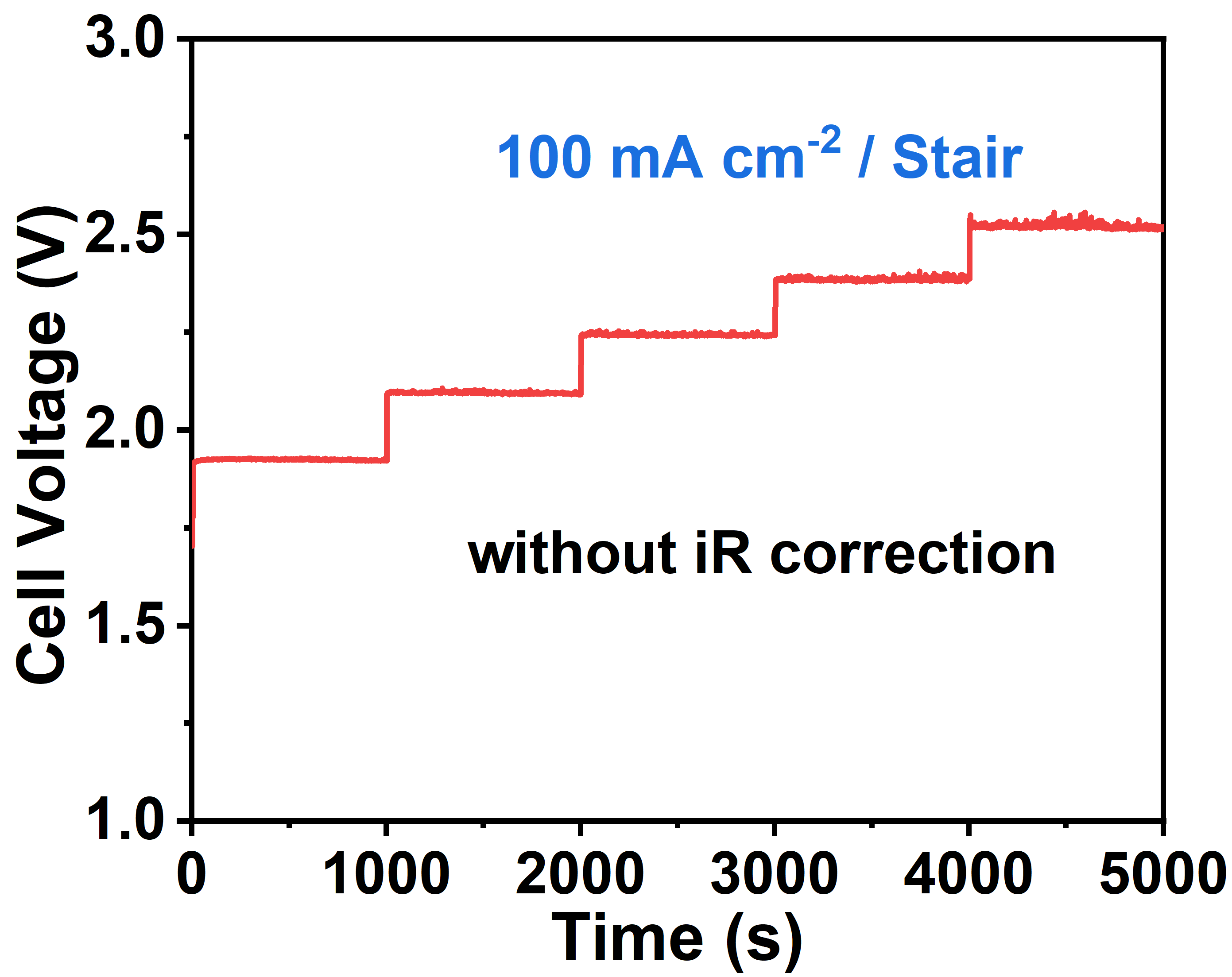


**Figure S34.** Multi-current process of NiCoMoPO for overall freshwater splitting without iR correction.





**Figure S35.** Chronopotentiometry curve for overall freshwater splitting of NiCoMoPO at 500 mA cm^−2^.


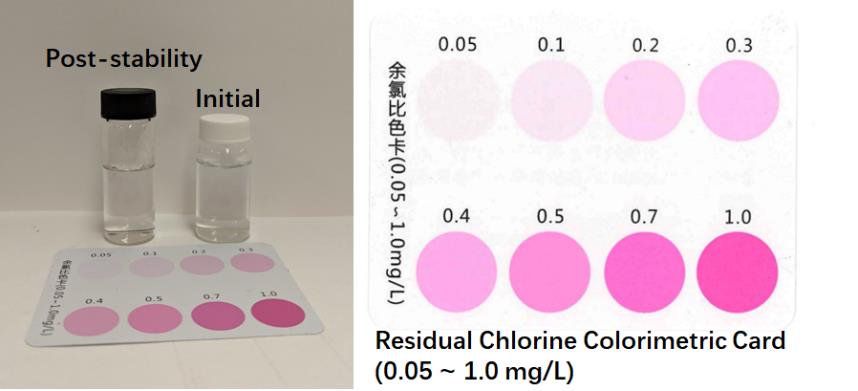


**Figure S36.** Checking the absence of ClO^−^ production by N, N-diethyl-p-phenylenediamine (DPD) reagent after the stability test of overall seawater splitting at 500 mA cm^−2^ for 1000 h in alkaline seawater.


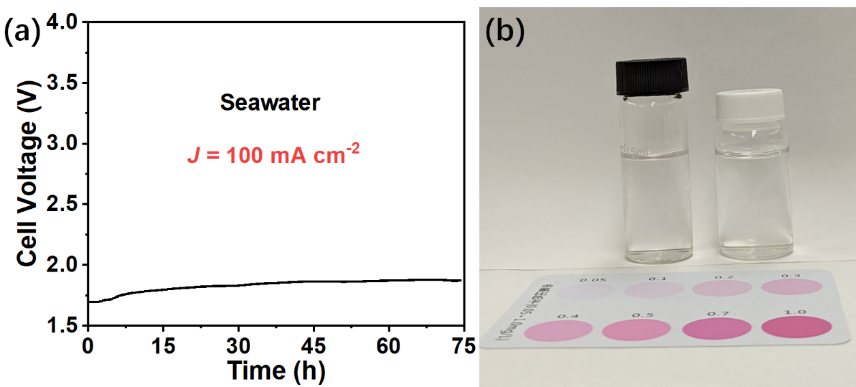


**Figure S37.** (a) Chronopotentiometry curve for overall seawater splitting of Pt/C/NF//RuO_2_/NF (−, +) at 100 mA cm^−2^. (b) Checking the absence of ClO^−^ production by the DPD reagent after the stability test.


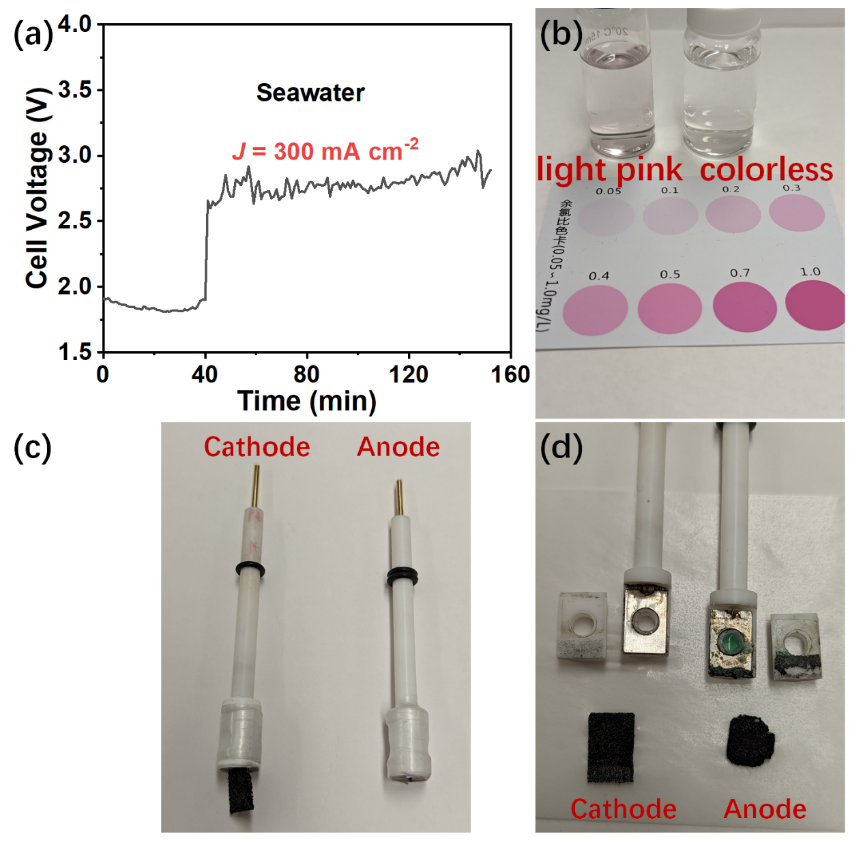


**Figure S38.** (a) Chronopotentiometry curve for overall seawater splitting of Pt/C/NF//RuO_2_/NF (−, +) at 300 mA cm^−2^. (b) Checking the absence of ClO^−^ production by the DPD reagent after the stability test (The light pink electrolyte indicates the generation of ClO^−^). (c, d) Images of the cathode and anode after the stability test.


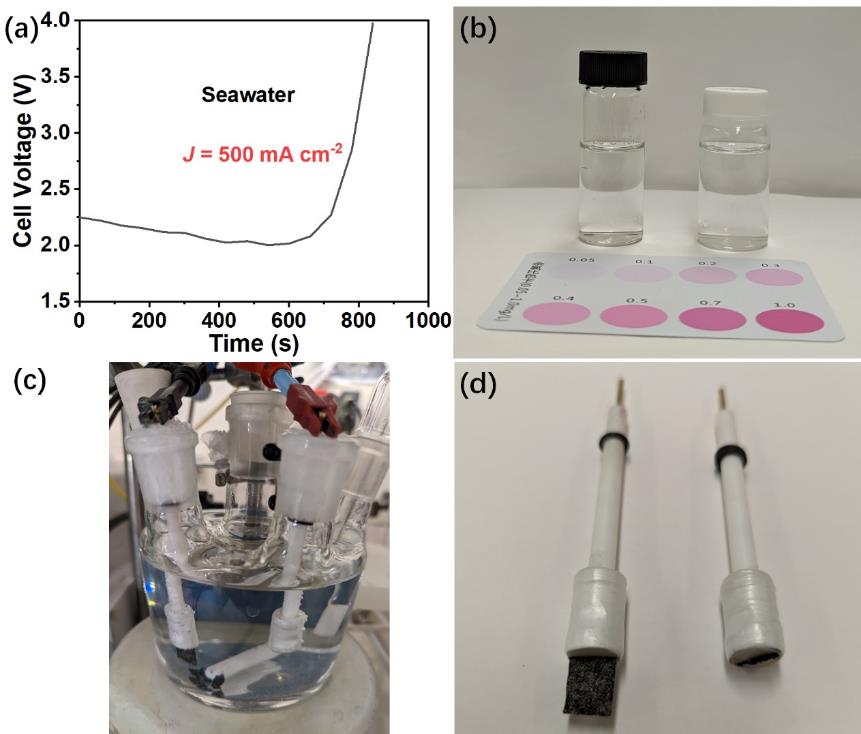


**Figure S39.** (a) Chronopotentiometry curve for overall seawater splitting of Pt/C/NF//RuO_2_/NF (−, +) at 500 mA cm^−2^. (b) Checking the absence of ClO^−^ production by the DPD reagent after the stability test. (c, d) Images of the cathode and anode after the stability test.


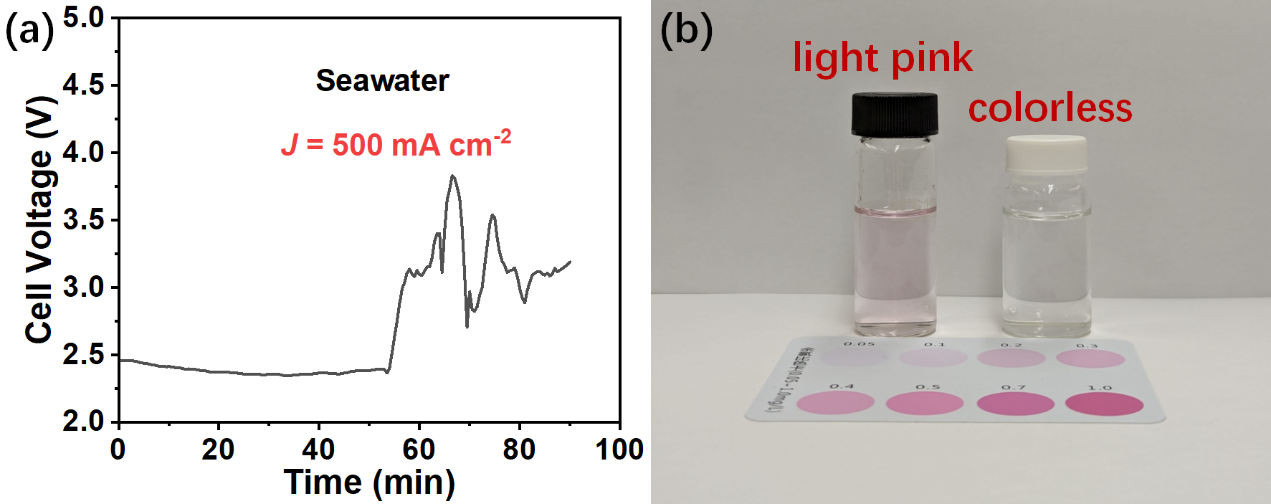


**Figure S40.** (a) Chronopotentiometry curve for overall seawater splitting of NiCoMoO//NiCoMoO (−, +) at 500 mA cm^−2^. (b) Checking the absence of ClO^−^ production by the DPD reagent after the stability test.


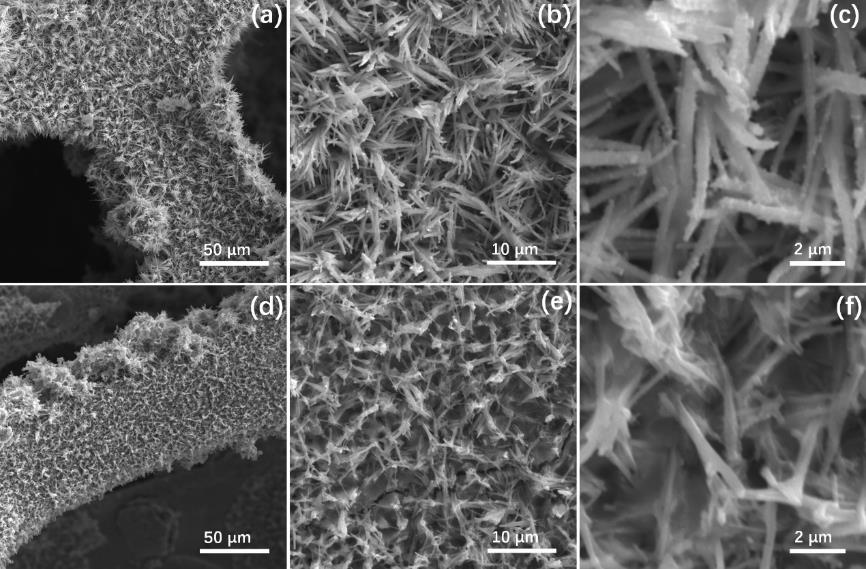


**Figure S41.** SEM images of the (a–c) cathode and (d–f) anode after the overall-seawater-splitting stability test.


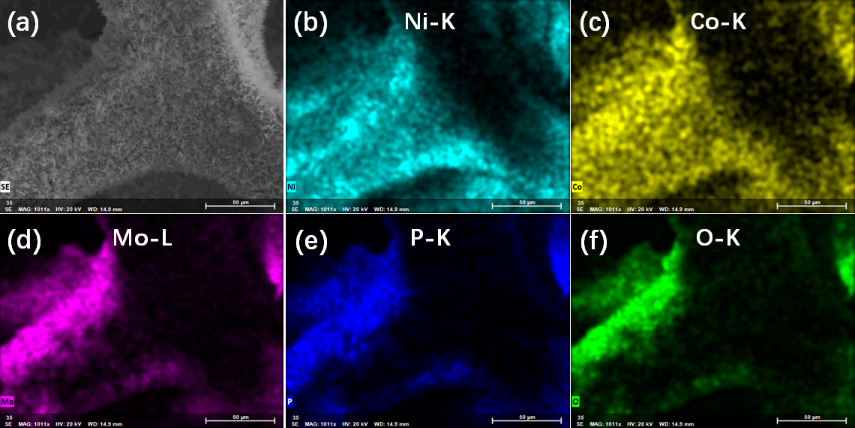


**Figure S42.** (a) SEM and (b–f) the corresponding elemental mapping images of the cathode after the overall-seawater-splitting stability test.


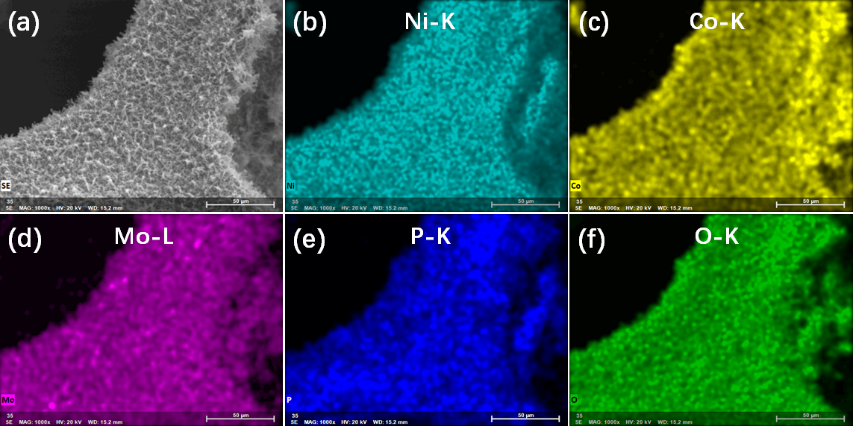


**Figure S43.** (a) SEM and (b–f) the corresponding elemental mapping images of the anode after the overall-seawater-splitting stability test.


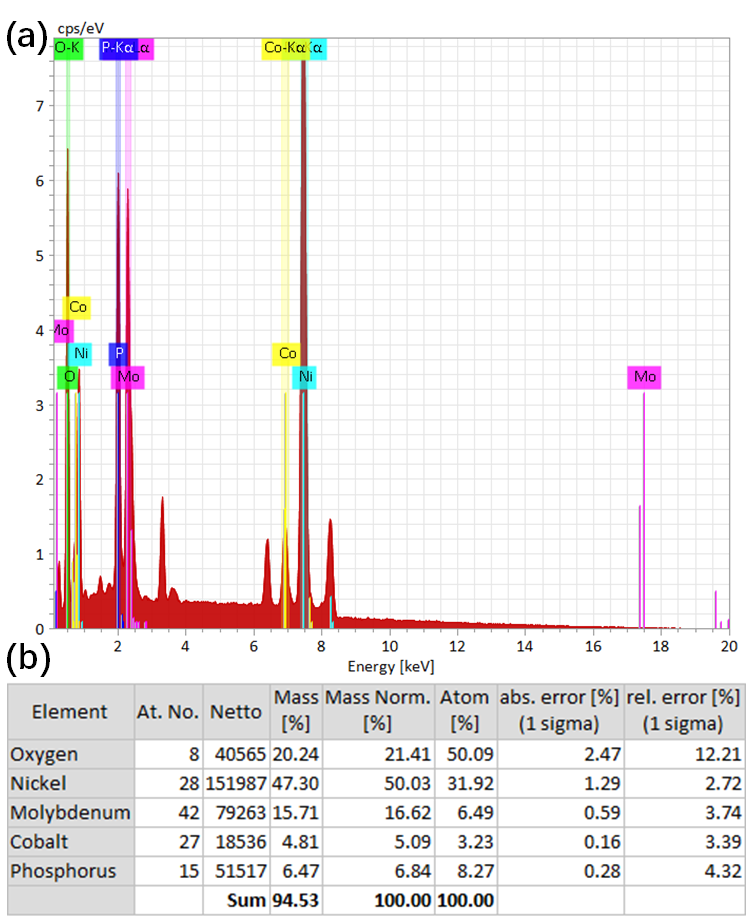


**Figure S44.** EDS and the corresponding elemental content of the cathode after the overall-seawater-splitting stability test.


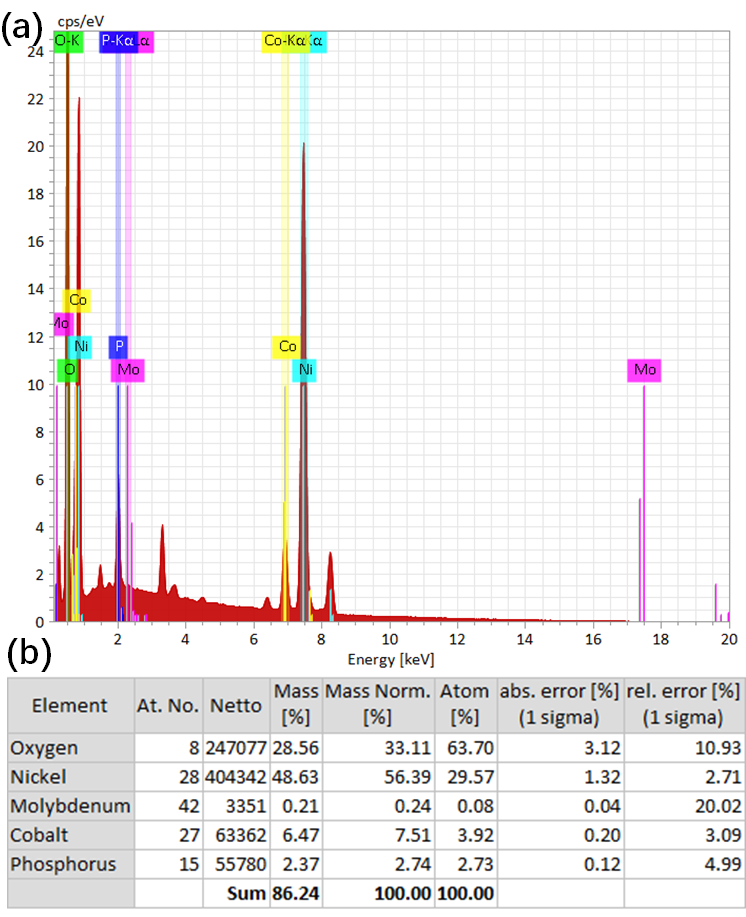


**Figure S45.** EDS and the corresponding elemental content of the anode after the overall-seawater-splitting stability test.


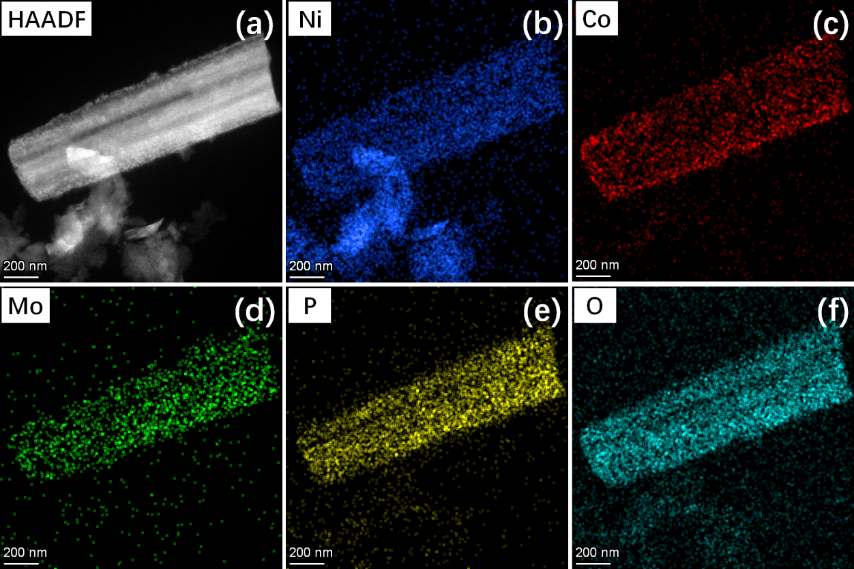


**Figure S46.** (a) HAADF-TEM and (b–f) the corresponding elemental mapping images of the cathode after the overall-seawater-splitting stability test.


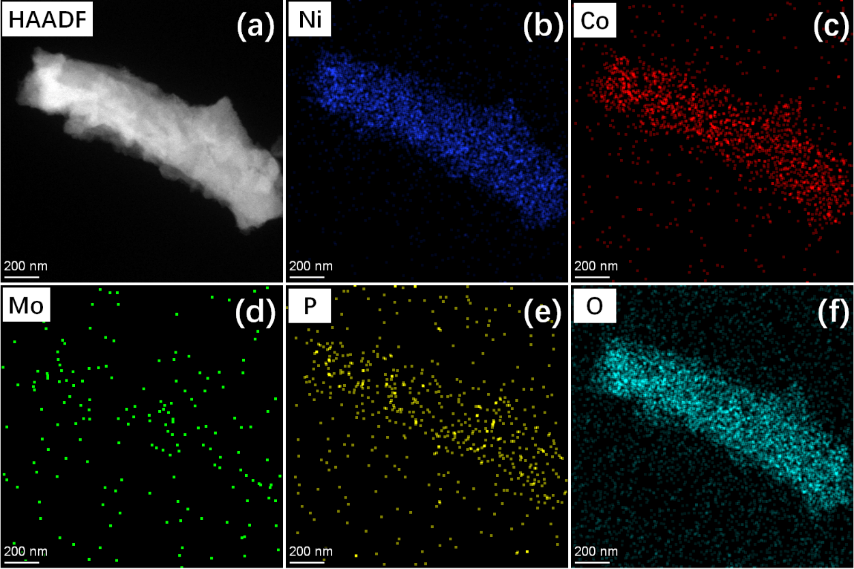


**Figure S47.** (a) HAADF-TEM and (b–f) the corresponding elemental mapping images of the anode after the overall-seawater-splitting stability test.


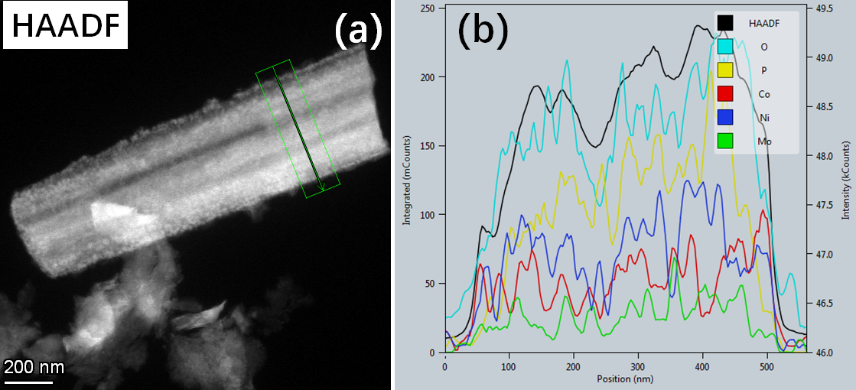


**Figure S48.** (a) HAADF-STEM image and (b) the corresponding elemental profiles of line-scan EELS of the cathode after the overall-seawater-splitting stability test.


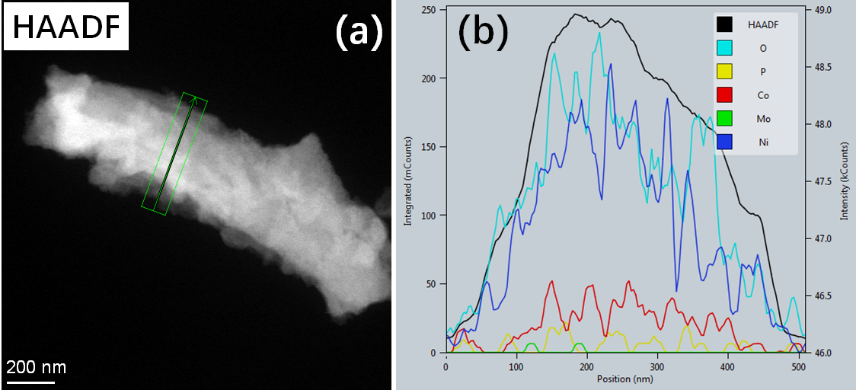


**Figure S49.** (a) HAADF-STEM image and (b) the corresponding elemental profiles of line-scan EELS of the anode after the overall-seawater-splitting stability test.


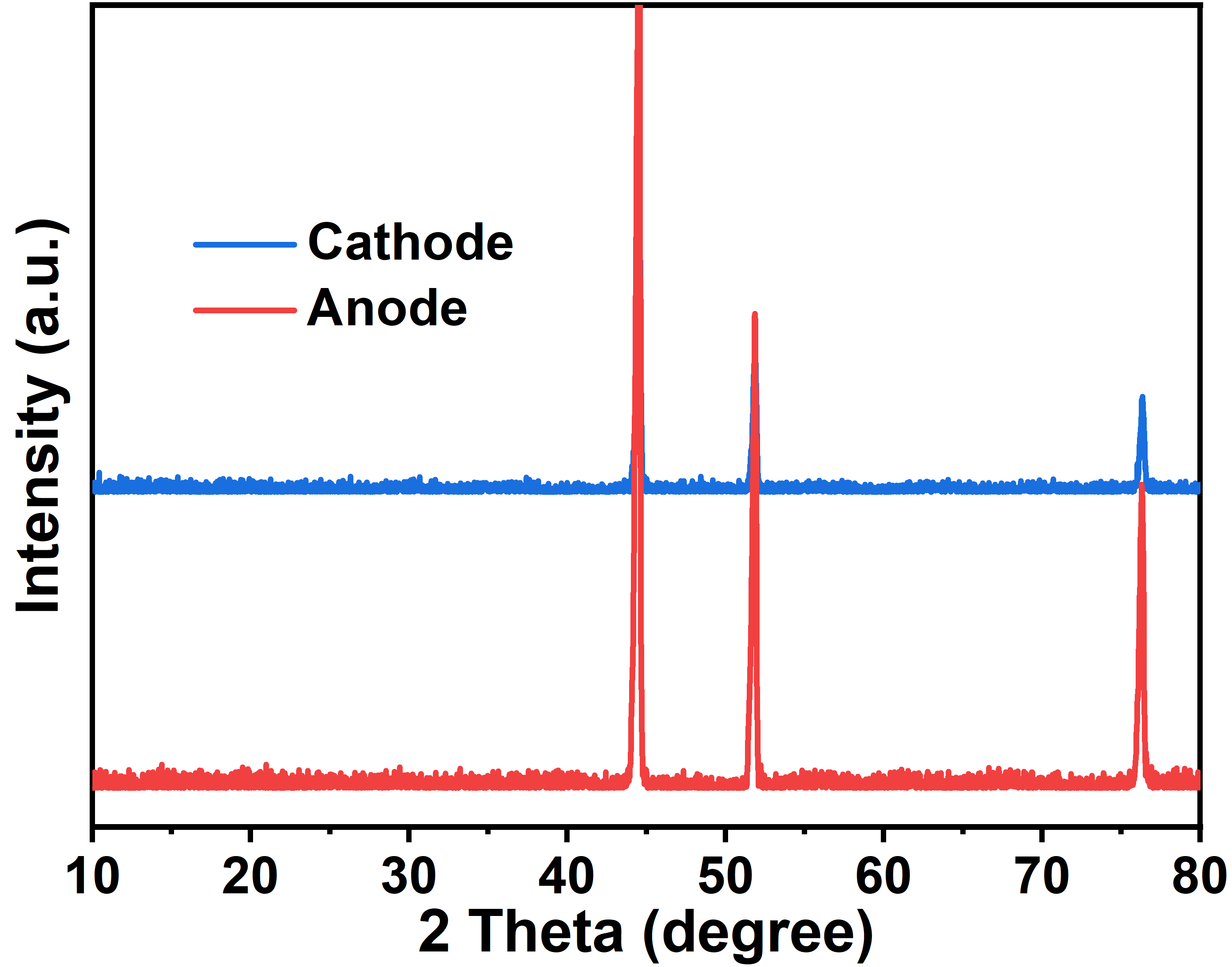


**Figure S50.** XRD patterns of the anode and cathode after the stability tests of overall seawater splitting.





**Figure S51.** High-resolution Co 2p XPS spectra of the anode and cathode after the overall-seawater-splitting stability test.





**Figure S52.** High-resolution Ni 2p XPS spectra of the anode and cathode after the overall-seawater-splitting stability test.





**Figure S53.** High-resolution Mo 3d XPS spectra of the anode and cathode after the overall-seawater-splitting stability test.





**Figure S54.** High-resolution P 2p XPS spectra of the anode and cathode after the overall-seawater-splitting stability test.





**Figure S55.** High-resolution O 1s XPS spectra of the anode and cathode after the overall-seawater-splitting stability test.





**Figure S56.** Normalized Mo *L*-edge XANES spectra of the initial NiCoMoO, anode, cathode, reference Mo foil, and reference MoO_3_.


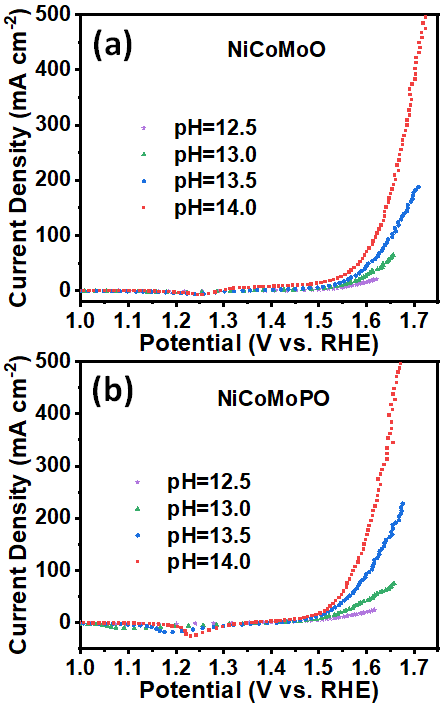


**Figure S57.** LSV curves of as-activated (a) NiCoMoO and (b) NiCoMoPO in different pH electrolytes.


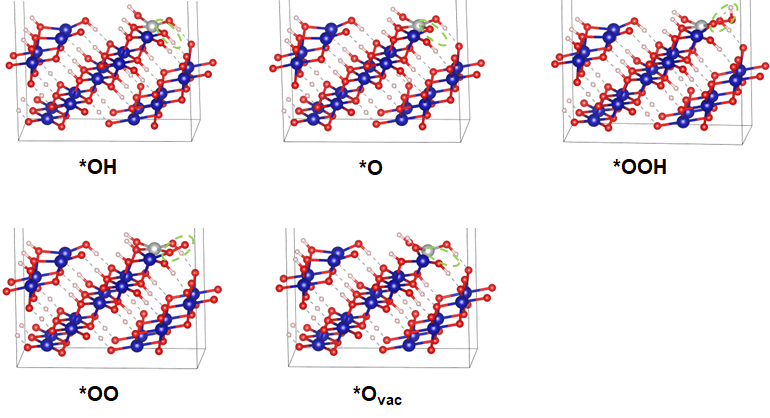


**Figure S58.** Optimized structures of (Ni)CoOOH model for LOM. The silver, blue, red, and light pink balls represent Ni, Co, O, and H, respectively.


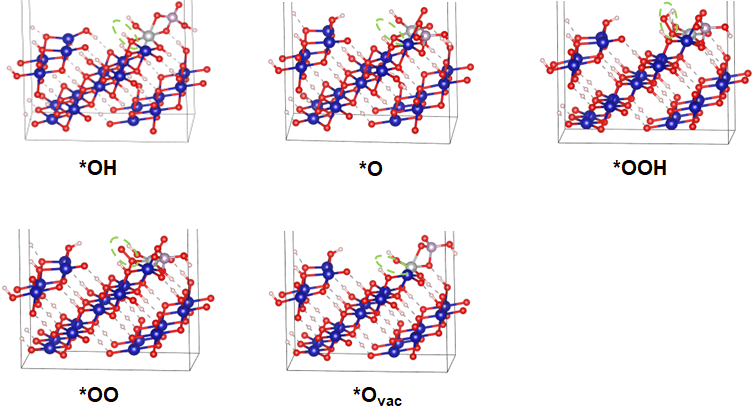


**Figure S59.** Optimized structures of PO_3_^−^‐(Ni)CoOOH model for LOM. The silver, blue, pink, red, and light pink balls represent Ni, Co, P, O, and H, respectively.

**Table S1.** Comparison of overpotentials for OER at 500 and 1000 mA cm^−2^ for recently non-noble electrocatalysts in alkaline freshwater.

| Electrocatalysts | Overpotentials (mV)  @500 mA cm^−2^ | Overpotentials (mV)  @1000 mA cm^−2^ | References |
| --- | --- | --- | --- |
| NiCoMoPO | 419 | 467 | This work |
| NiCoMoO | 518 | 631 | This work |
| Co_0.8_Ni_0.2_/NF | 430 | 500 | ^[8]^ |
| (Ni_7_Fe_3_)OOH-S | 399 | - | ^[9]^ |
| FeNi-MOF nanoarrays | 420 | - | ^[10]^ |
| FeOOH/NiFe LDHs@CCH NAs | 550 | - | ^[11]^ |
| C‐Ni_1−x_O/3DPNi | 390 | 425 | ^[12]^ |
| Ni-Fe-OH@Ni_3_S_2_/NF | 450 | - | ^[13]^ |
| Ni_x_Co_3−_xS_4_/Ni_3_S_2_/Ni Foam | 480 | - | ^[14]^ |
| Co_2.3_Fe_0.7_O_4_-NSs/CFP | 650 | - | ^[15]^ |
| Ni_x_S_y_/NF | 530 | - | ^[16]^ |
| NiFeNi(O) | 430 | - | ^[17]^ |
| FeCoNiWCuOOH@Cu | 570 | - | ^[18]^ |
| Ni_2_P-NiSe_2_/MoO_x_/NF | 592 | - | ^[19]^ |

**Table S2.** Comparison of overpotentials for HER at 500 and 1000 mA cm^−2^ for recently non-noble electrocatalysts in alkaline freshwater.

| Electrocatalysts | Overpotentials (mV)  @500 mA cm^−2^ | Overpotentials (mV)  @1000 mA cm^−2^ | References |
| --- | --- | --- | --- |
| NiCoMoPO | 368 | 442 | This work |
| NiCoMoO | 485 | 566 | This work |
| GDY/MoO_3_ | 1100 | 1.7 | ^[20]^ |
| HC-MoS_2_/Mo_2_C | 390 | 440 | ^[21]^ |
| α-MoB_2_ | - | 334 | ^[22]^ |
| Fe-Ni_2_P/NF | 389 | 468 | ^[23]^ |
| N-MoO_2_/Ni_3_S_2_/NF | 430 | - | ^[24]^ |
| NC/Ni_3_Mo_3_N/NF | 400 | 700 | ^[25]^ |
| Sn–Ni(OH)_2_ | 480 | 550 | ^[26]^ |
| Ni_2_P–NiMoOx/NF | 297 | - | ^[27]^ |
| FeNi/NF | 600 | - | ^[8]^ |
| Sn−Ni_3_S_2_/NF | 380 | - | ^[28]^ |
| Ni_0.96_Co_0.04_P nanosheet | 161.1 | 249.7 | ^[29]^ |
| FeCoNiWCuOOH@Cu | 290 | - | ^[18]^ |
| FeCoP/CF | 410 | - | ^[30]^ |

**Table S3.** Comparison of OER stability at 500 and 1000 mA cm^−2^ for recently non-noble electrocatalysts in alkaline freshwater.

| Electrocatalysts | Stability(h)  @500 mA cm^−2^ | Stability(h)  @1000 mA cm^−2^ | References |
| --- | --- | --- | --- |
| NiCoMoPO | 200 | 300 | This work |
| FeNi-MOF nanoarrays | 200 | - | ^[10]^ |
| NiMoO_x_/NiMoS | 25 | - | ^[31]^ |
| NiSe_2_/NiFe_2_Se_4_@NiFe | 11 | - | ^[32]^ |
| Ni‐FeOOH  @NiFe alloy nanowire | 24 | 24 | ^[33]^ |
| Fe_2_O_3_@Ni_2_P/Ni(PO_3_)_2_ hybrid | - | 8 | ^[34]^ |
| Ni-Fe-OH@Ni_3_S_2_/NF | 50 | 50 | ^[13]^ |
| Ni–Fe–S | - | 12 | ^[35]^ |
| Fe_11.8%_-Ni_3_S_2_/NF | 14 | - | ^[36]^ |
| NiCo-OH@Ni_x_Fe_y_O_4_/FF | - | 100 | ^[37]^ |
| NiFe-MOF-74/NiFe-LDH | - | 10 | ^[38]^ |
| (Ni_7_Fe_3_)OOH-S | 400 | - | ^[9]^ |
| Ce-NiMo-PS@NF | 50 | - | ^[39]^ |
| NiFe‐LDH@NiMo‐H_2_ | 400 | - | ^[40]^ |

**Table S4.** Comparison of HER stability at 500 and 1000 mA cm^−2^ for recently non-noble electrocatalysts in alkaline freshwater.

| Electrocatalysts | Stability(h)  @500 mA cm^−2^ | Stability(h)  @1000 mA cm^−2^ | References |
| --- | --- | --- | --- |
| NiCoMoPO | 170 | 300 | This work |
| Ni_0.95_Cu_0.05_DSS | - | 110 | ^[41]^ |
| GDY/MoO_3_ | - | 24 | ^[20]^ |
| HC-MoS_2_/Mo_2_C | - | 24 | ^[21]^ |
| NiMoOx/NiMoS | 25 | - | ^[31]^ |
| Ni_2_P | - | 2.7 | ^[42]^ |
| N-MoO_2_/Ni_3_S_2_ | 10 | - | ^[24]^ |
| α-MoB_2_ | - | 6 | ^[22]^ |
| MoS_1.7_ | - | 24 | ^[43]^ |
| Co_0.59_Ni_0.41_C_2_O_4_@  PANI/NF | 30 | 30 | ^[44]^ |
| NC@NiNPs | - | 260 | ^[45]^ |
| Ni_3_S_2_/Cr_2_S_3_@NF | - | 35 | ^[46]^ |
| Ni_2_P/WO_2.83_ | - | 50 | ^[47]^ |
| Co-P_0.43_@NF | 150 | - | ^[48]^ |
| Ce-NiMo-PS@NF | 50 | - | ^[39]^ |

**Table S5.** Comparison of overpotentials for OER at 100 and 500 mA cm^−2^ for recently non-noble electrocatalysts in alkaline seawater.

| Electrocatalysts | Overpotentials (mV)  @100 mA cm^−2^ | Overpotentials (mV)  @500 mA cm^−2^ | References |
| --- | --- | --- | --- |
| NiCoMoPO | 349 | 452 | This work |
| Fe-Co-S/Cu_2_O/Cu | 440 | - | ^[49]^ |
| Co_2_(OH)_3_Cl | 368 | - | ^[50]^ |
| NiCoP/NiCo-LDH | 520 | - | ^[51]^ |
| Fe_2_P/Ni_1.5_Co_1.5_N/Ni_2_P | 255 | 307 | ^[52]^ |
| Co-N_3_P_1_ | 490 | - | ^[53]^ |
| Ni_2_P-Fe_2_P/NF | 305 | 380 | ^[54]^ |
| MoN–Co_2_N/NF | 357 | 432 | ^[55]^ |
| CoSe_2_-NCF | 455 | - | ^[56]^ |
| H-CoFeSx | 380 | - | ^[57]^ |
| B-MnFe_2_O_4_@MFOC | 405 | - | ^[58]^ |
| MoO_3_@CoO/CC | 480 | - | ^[59]^ |
| Co/Mo–N–C/Cu | 290 | 380 | ^[60]^ |

**Table S6.** Comparison of overpotentials for HER at 100, 500, and 1000 mA cm^−2^ for recently non-noble electrocatalysts in alkaline seawater.

| Electrocatalysts | Overpotentials  (mV)@100 mA cm^−2^ | Overpotentials (mV)@500 mA cm^−2^ | Overpotentials (mV)@1000 mA cm^−2^ | References |
| --- | --- | --- | --- | --- |
| NiCoMoPO | 275 | 404 | 470 | This work |
| MoN–Co_2_N/NF | 304 | 451 | - | ^[55]^ |
| CoSe_2_-NCF | 463 | - | - | ^[56]^ |
| 3%Er-MoO_2_ | 284 | - | - | ^[61]^ |
| Co-N_3_P_1_ | 287 | - | - | ^[53]^ |
| NiCoP/NiCo-LDH | 310 | - | - | ^[51]^ |
| Mn-doped Ni_2_P/Fe_2_P | 308 | 425 | 470 | ^[62]^ |
| Ni-SA/NC | 280 | - | - | ^[63]^ |
| NiFeSP | 276 | 380 | - | ^[64]^ |
| Ni/Co-CuOx | 180 | - | - | ^[65]^ |
| NiFeS/NF | 217 | 347 | - | ^[66]^ |
| FeMoSe@NiCo-LDH | 455 | - | - | ^[67]^ |
| CeOx@NiCo_2_O_4_/NF | 300 | - | - | ^[68]^ |

**Table S7.** Comparison of cell voltages for overall water splitting at 100 and 500 mA cm^−2^ for recently non-noble electrocatalysts in alkaline freshwater and seawater.

| Electrocatalysts | Electrolyte | Cell voltages (V)  @100 mA cm^−2^ | Cell voltages (V)  @500 mA cm^−2^ | References |
| --- | --- | --- | --- | --- |
| NiCoMoPO | freshwater | 1.839 | 2.024 | This work |
| NiCoMoPO | seawater | 1.850 | 2.096 | This work |
| P-Fe_2_O_3_-CoP | freshwater | 1.80 | - | ^[69]^ |
| P-Fe_2_O_3_-CoP | seawater | 1.86 | - | ^[69]^ |
| P-Fe_3_O_4_-MoO_2_/N-doped carbon | freshwater | 1.69 | - | ^[70]^ |
| P-Fe_3_O_4_-MoO_2_/N-doped carbon | seawater | 1.88 | - | ^[70]^ |
| Co-N_3_P_1_ | seawater | 2.07 | - | ^[53]^ |
| Ni_2_P-Fe_2_P | freshwater | 1.682 | 1.865 | ^[54]^ |
| Ni_2_P-Fe_2_P | seawater | 1.811 | 2.004 | ^[54]^ |
| NiP_x_@hydrophobic asbestos | seawater | - | 2.37 | ^[71]^ |
| MnCo/NiSe | seawater | - | 2.025 | ^[72]^ |
| Mo-CoP_x_/NF | freshwater | 2.01 | - | ^[73]^ |
| Mo-CoP_x_/NF | seawater | 2.16 | - | ^[73]^ |
| Er-MoO_2_ | seawater | 2.08 | - | ^[61]^ |
| Co–P@NN | seawater | - | 2.161 | ^[74]^ |
| NiFe LDH/FeOOH | freshwater | 1.85 | - | ^[75]^ |
| NiFe LDH/FeOOH | seawater | 1.87 | - | ^[75]^ |
| Ni_2_P-NiSe_2_/MoO_x_/NF | freshwater | 1.76 | - | ^[19]^ |
| NiCo_2_S_4_/NiMn‐LDH | freshwater | 1.95 | - | ^[76]^ |
| NiCo_2_S_4_/NiMn‐LDH | seawater | 1.99 | - | ^[76]^ |
| Co_4_N–Ni_3_N | freshwater | 1.97 | - | ^[77]^ |
| Co_4_N–Ni_3_N | seawater | 2.19 | - | ^[77]^ |
| NiFeP/NiP | freshwater | 1.90 | - | ^[78]^ |
| NiFeP/NiP | seawater | 2.02 | - | ^[78]^ |
| Ru-P-Ni(OH)_2_/NF | freshwater | 1.92 | 2.88 | ^[79]^ |
| Ru-P-Ni(OH)_2_/NF | seawater | 1.94 | - | ^[79]^ |

**Table S8.** Comparison of stability at 500 and 1000 mA cm^−2^ of NiCoMoPO and recently non-noble electrocatalysts for alkaline overall freshwater splitting.

| Electrocatalysts | Stability(h)  @500 mA cm^−2^ | Stability(h)  @1000 mA cm^−2^ | References |
| --- | --- | --- | --- |
| NiCoMoPO | 200 | 300 | This work |
| Ni_3_N@2M-MoS_2_ | - | 300 | ^[80]^ |
| NiMoOx/NiMoS | 500 | - | ^[31]^ |
| NiSe_2_/NiFe_2_Se_4_@NiFe | 10 | 11 | ^[32]^ |
| Ni–Fe oxyhydroxide  @NiFe alloy nanowire | 24 | - | ^[33]^ |
| C–Ni_1−x_O/3DPNi | - | 16 | ^[12]^ |
| MFN-MOFs(2:1)/NF | 100 | - | ^[81]^ |
| Fe_2_O_3_@Ni_2_P/Ni(PO_3_)_2_ hybrid | - | 10 | ^[34]^ |
| Fe^0^-NixSy/NF | - | 10 | ^[82]^ |
| Ni_0.96_Co_0.04_P  nanosheet | 500 | - | ^[29]^ |
| Ni-MoN | 40 | - | ^[83]^ |
| Fe_2_P/Co_2_N | 120 | - | ^[84]^ |
| Ni_2_P-NiMoO_x_ | 12 | - | ^[27]^ |
| Mn-doped Ni_2_P/Fe_2_P | 25 | - | ^[62]^ |
| Ce-doped triphasic NiMoP_2_/NiMo_3_S_4_/NiMoO_4_ | 50 | - | ^[39]^ |

**Table S9.** Comparison of stability at 500 mA cm^−2^ of NiCoMoPO and recently reported non-noble electrocatalysts for OER, HER, and overall water splitting in alkaline seawater.

| Electrocatalysts | OER  stability(h) | HER  stability(h) | Overall  Seawater splitting stability(h) | References |
| --- | --- | --- | --- | --- |
| NiCoMoPO | 160 | 160 | 1200 | This work |
| CoP_x_@FeOOH | 80 | - | - | ^[85]^ |
| CoP_x_ | - | 15 | - | ^[85]^ |
| CoP_x_\|\|CoP_x_  @FeOOH | - | - | 80 | ^[85]^ |
| Ni_2_P-Fe_2_P/NF | - | - | 38 | ^[54]^ |
| NiFeS/NF | 24 | 25 | 25 | ^[66]^ |
| Fe–NiS/NF | 25 | 25 | 50 | ^[86]^ |
| Co, Cl-Cu(OH)_2_ | 20 | - | - | ^[87]^ |
| Mn-doped  Ni_2_P/Fe_2_P | 200 | 120 | 120 | ^[62]^ |
| Fe_2_P/Ni_3_N | 40 | 40 | 40 | ^[88]^ |
| MnCo/NiSe | 200 | 200 | 200 | ^[72]^ |
| Fe_4_N/Co_3_N/MoO_2_ | 40 | 70 | 200 | ^[89]^ |
| Ni/NF  @Mo_2_N/FeO_x_N_y_ | - | - | 50 | ^[90]^ |
| P-doped graphene quantum dots | - | - | 1000 | ^[91]^ |

**References**

[1] a) G. Zhang, B. Wang, L. Li, S. Yang, *Small* **2019**, *15*, 1904105-1904113; b) F. Lin, Z. Dong, Y. Yao, L. Yang, F. Fang, L. Jiao, *Adv. Energy Mater.* **2020**, *10*, 2002176.

[2] a) G. Kresse, J. Furthmüller, *Comput. Mater. Sci* **1996**, *6*, 15-50; b) G. Kresse, J. Furthmüller, *Phys. Rev. B* **1996**, *54*, 11169-11186.

[3] J. P. Perdew, K. Burke, M. Ernzerhof, *Phys. Rev. Lett.* **1996**, *77*, 3865-3868.

[4] M. Bajdich, M. García-Mota, A. Vojvodic, J. K. Nørskov, A. T. Bell, *J. Am. Chem. Soc.* **2013**, *135*, 13521-13530.

[5] L. Yang, H. Xu, H. Liu, D. Cheng, D. Cao, *Small Methods* **2019**, *3*, 1900113.

[6] S. Grimme, J. Antony, S. Ehrlich, H. Krieg, *J. Chem. Phys.* **2010**, *132*, 154104.

[7] D. Friebel, M. W. Louie, M. Bajdich, K. E. Sanwald, Y. Cai, A. M. Wise, M.-J. Cheng, D. Sokaras, T.-C. Weng, R. Alonso-Mori, R. C. Davis, J. R. Bargar, J. K. Nørskov, A. Nilsson, A. T. Bell, *J. Am. Chem. Soc.* **2015**, *137*, 1305-1313.

[8] T. E. Seuferling, T. R. Larson, J. M. Barforoush, K. C. Leonard, *ACS Sustain. Chem. Eng.* **2021**, *9*, 16678-16686.

[9] W. Liu, X. Wang, F. Wang, X. Liu, Y. Zhang, W. Li, Y. Guo, H. Yin, D. Wang, *Chem. Eng. J.* **2023**, *454*, 140030.

[10] C.-P. Wang, Y. Feng, H. Sun, Y. Wang, J. Yin, Z. Yao, X.-H. Bu, J. Zhu, *ACS Catal.* **2021**, *11*, 7132-7143.

[11] J. Chi, H. Yu, G. Jiang, J. Jia, B. Qin, B. Yi, Z. Shao, *J. Mater. Chem. A* **2018**, *6*, 3397-3401.

[12] T. Kou, S. Wang, R. Shi, T. Zhang, S. Chiovoloni, J. Q. Lu, W. Chen, M. A. Worsley, B. C. Wood, S. E. Baker, E. B. Duoss, R. Wu, C. Zhu, Y. Li, *Adv. Energy Mater.* **2020**, *10*, 2002955.

[13] X. Zou, Y. Liu, G. D. Li, Y. Wu, D. P. Liu, W. Li, H. W. Li, D. Wang, Y. Zhang, X. Zou, *Adv. Mater.* **2017**, *29*, 1700404-1700410.

[14] Y. Wu, Y. Liu, G.-D. Li, X. Zou, X. Lian, D. Wang, L. Sun, T. Asefa, X. Zou, *Nano Energy* **2017**, *35*, 161-170.

[15] Z. Ye, C. Qin, G. Ma, X. Peng, T. Li, D. Li, Z. Jin, *ACS Appl. Mater. Interfaces* **2018**, *10*, 39809-39818.

[16] X. Cheng, C. Lei, J. Yang, B. Yang, Z. Li, J. Lu, X. Zhang, L. Lei, Y. Hou, K. Ostrikov, *ChemElectroChem* **2018**, *5*, 3866-3872.

[17] L. Chen, Y. Wang, X. Zhao, Y. Wang, Q. Li, Q. Wang, Y. Tang, Y. Lei, *J. Mater. Sci. Technol.* **2022**, *110*, 128-135.

[18] C. X. Zhang, D. Yin, Y. X. Zhang, Y. X. Sun, X. J. Zhao, W. G. Liao, J. C. Ho, *Adv. Sci.* **2024**, 2406008.

[19] J.-T. Ren, L. Chen, H.-Y. Wang, W.-W. Tian, S.-X. Zhai, Y. Feng, Z.-Y. Yuan, *Appl. Catal. B: Environ.* **2024**, *347*, 123817.

[20] Y. Yao, Y. Zhu, C. Pan, C. Wang, S. Hu, W. Xiao, X. Chi, Y. Fang, J. Yang, H. Deng, S. Xiao, J. Li, Z. Luo, Y. Guo, *J. Am. Chem. Soc.* **2021**, *143*, 8720–8730.

[21] C. Zhang, Y. Luo, J. Tan, Q. Yu, F. Yang, Z. Zhang, L. Yang, H.-M. Cheng, B. Liu, *Nat. Commun.* **2020**, *11*, 3724-3731.

[22] Y. Chen, G. Yu, W. Chen, Y. Liu, G.-D. Li, P. Zhu, Q. Tao, Q. Li, J. Liu, X. Shen, H. Li, X. Huang, D. Wang, T. Asefa, X. Zou, *J. Am. Chem. Soc.* **2017**, *139*, 12370–12373.

[23] D. Li, Z. Li, R. Zou, G. Shi, Y. Huang, W. Yang, W. Yang, C. Liu, X. Peng, *Appl. Catal. B: Environ.* **2022**, *307*, 121170.

[24] L. Wang, J. Cao, C. Lei, Q. Dai, B. Yang, Z. Li, X. Zhang, C. Yuan, L. Lei, Y. Hou, *ACS Appl. Mater. Interfaces* **2019**, *11*, 27743-27750.

[25] Y. Chen, J. Yu, J. Jia, F. Liu, Y. Zhang, G. Xiong, R. Zhang, R. Yang, D. Sun, H. Liu, W. Zhou, *Appl. Catal. B: Environ.* **2020**, *272*, 118956.

[26] J. Jian, X. Kou, H. Wang, L. Chang, L. Zhang, S. Gao, Y. Xu, H. Yuan, *ACS Appl. Mater. Interfaces* **2021**, *13*, 42861-42869.

[27] J.-T. Ren, L. Chen, H.-Y. Wang, W.-W. Tian, X.-L. Song, Q.-H. Kong, Z.-Y. Yuan, *ACS Catal.* **2023**, *13*, 9792-9805.

[28] J. Jian, L. Yuan, H. Qi, X. Sun, L. Zhang, H. Li, H. Yuan, S. Feng, *ACS Appl. Mater. Interfaces* **2018**, *10*, 40568-40576.

[29] X. Lv, S. Wan, T. Mou, X. Han, Y. Zhang, Z. Wang, X. Tao, *Adv. Funct. Mater.* **2023**, *33*, 2205161.

[30] Y. Xu, Y. Zhao, M. Sun, W. Xie, Y. Wu, G. Cheng, Y. Zhong, S. Han, L. Yu, *Chem. Eng. J.* **2024**, *490*, 151697.

[31] P. Zhai, Y. Zhang, Y. Wu, J. Gao, B. Zhang, S. Cao, Y. Zhang, Z. Li, L. Sun, J. Hou, *Nat. Commun.* **2020**, *11*, 5462.

[32] J. Yuan, X. Cheng, H. Wang, C. Lei, S. Pardiwala, B. Yang, Z. Li, Q. Zhang, L. Lei, S. Wang, Y. Hou, *Nano-Micro Lett.* **2020**, *12*, 104-115.

[33] C. Liang, P. Zou, A. Nairan, Y. Zhang, J. Liu, K. Liu, S. Hu, F. Kang, H. J. Fan, C. Yang, *Energy Environ. Sci.* **2020**, *13*, 86-95.

[34] X. Cheng, Z. Pan, C. Lei, Y. Jin, B. Yang, Z. Li, X. Zhang, L. Lei, C. Yuan, Y. Hou, *J. Mater. Chem. A* **2019**, *7*, 965-971.

[35] J. Zhang, Y. Hu, D. Liu, Y. Yu, B. Zhang, *Adv. Sci.* **2017**, *4*, 1600343.

[36] N. Cheng, Q. Liu, A. M. Asiri, W. Xing, X. Sun, *J. Mater. Chem. A* **2015**, *3*, 23207-23212.

[37] Z. Li, X. Zhang, Z. Zhang, P. Chen, Y. Zhang, X. Dong, *Appl. Catal. B: Environ.* **2023**, *325*, 122311.

[38] G. Mu, G. Wang, Q. Huang, Y. Miao, D. Wen, D. Lin, C. Xu, Y. Wan, F. Xie, W. Guo, R. Zou, *Adv. Funct. Mater.* **2023**, *33*, 2211260.

[39] Y. Cheng, A. Yuan, Y. Zhang, H. Liu, J. Du, L. Chen, *J. Colloid Interface Sci.* **2024**, *660*, 166-176.

[40] Y. Zhang, B. Feng, M. Yan, Z. Shen, Y. Chen, J. Tian, F. Xu, G. Chen, X. Wang, L. Yang, Q. Wu, Z. Hu, *Nano Res.* **2024**, *17*, 3769-3776.

[41] X. Zhang, J. Wang, J. Wang, J. Wang, C. Wang, C. Lu, *J. Phys. Chem. Lett.* **2021**, 11135-11142.

[42] X. Yu, Z. Y. Yu, X. L. Zhang, Y. R. Zheng, Y. Duan, Q. Gao, R. Wu, B. Sun, M. R. Gao, G. Wang, S. H. Yu, *J. Am. Chem. Soc.* **2019**, *141*, 7537-7543.

[43] A.-Y. Lu, X. Yang, C.-C. Tseng, S. Min, S.-H. Lin, C.-L. Hsu, H. Li, H. Idriss, J.-L. Kuo, K.-W. Huang, L.-J. Li, *Small* **2016**, *12*, 5530-5537.

[44] S. Zhao, L. Yin, L. Deng, J. Song, Y. M. Chang, F. Hu, H. Wang, H. Y. Chen, L. Li, S. Peng, *Adv. Funct. Mater.* **2022**, *33*, 2211576.

[45] Y. Tang, F. Liu, W. Liu, S. Mo, X. Li, D. Yang, Y. Liu, S.-J. Bao, *Appl. Catal. B: Environ.* **2023**, *321*, 122081.

[46] H. Q. Fu, M. Zhou, P. F. Liu, P. Liu, H. Yin, K. Z. Sun, H. G. Yang, M. Al-Mamun, P. Hu, H. F. Wang, H. Zhao, *J. Am. Chem. Soc.* **2022**, *144*, 6028-6039.

[47] Y. Zhou, R. Li, L. Dong, S. Yin, B. Chu, Z. Chen, J. Wang, B. Li, M. Fan, *ACS Appl. Mater. Interfaces* **2022**, *14*, 18816-18824.

[48] Y. Shi, M. Wang, D. Zhang, H. Li, C. Li, T. Zhan, J. Lai, L. Wang, *Adv. Funct. Mater.* **2024**, 2410825.

[49] J. Sun, P. Song, H. Zhou, L. Lang, X. Shen, Y. Liu, X. Cheng, X. Fu, G. Zhu, *Appl. Surf. Sci.* **2021**, *567*, 150757.

[50] L. Zhuang, J. Li, K. Wang, Z. Li, M. Zhu, Z. Xu, *Adv. Funct. Mater.* **2022**, *32*, 2201127.

[51] Y. Wu, Z. Tian, S. Yuan, Z. Qi, Y. Feng, Y. Wang, R. Huang, Y. Zhao, J. Sun, W. Zhao, W. Guo, J. Feng, J. Sun, *Chem. Eng. J.* **2021**, *411*, 128538.

[52] F. Zhang, Y. Liu, F. Yu, H. Pang, X. Zhou, D. Li, W. Ma, Q. Zhou, Y. Mo, H. Zhou, *ACS Nano* **2023**, *17*, 1681-1692.

[53] X. Wang, X. Zhou, C. Li, H. Yao, C. Zhang, J. Zhou, R. Xu, L. Chu, H. Wang, M. Gu, H. Jiang, M. Huang, *Adv. Mater.* **2022**, *34*, 2204021.

[54] L. Wu, L. Yu, F. Zhang, B. McElhenny, D. Luo, A. Karim, S. Chen, Z. Ren, *Adv. Funct. Mater.* **2020**, *31*, 2006484-2006495.

[55] X. Wang, X. Han, R. Du, C. Xing, X. Qi, Z. Liang, P. Guardia, J. Arbiol, A. Cabot, J. Li, *ACS Appl. Mater. Interfaces* **2022**, *14*, 41924-41933.

[56] H. Chen, S. Zhang, Q. Liu, P. Yu, J. Luo, G. Hu, X. Liu, *Inorg. Chem. Commun.* **2022**, *146*, 110170.

[57] J. Li, T. Yu, K. Wang, Z. Li, J. He, Y. Wang, L. Lei, L. Zhuang, M. Zhu, C. Lian, Z. Shao, Z. Xu, *Adv. Sci.* **2022**, *9*, 2202387.

[58] M. Chen, N. Kitiphatpiboon, C. Feng, Q. Zhao, A. Abudula, Y. Ma, K. Yan, G. Guan, *Appl. Catal. B: Environ.* **2023**, *330*, 122577.

[59] L. Zhou, D. Guo, L. Wu, Z. Guan, C. Zou, H. Jin, G. Fang, X. Chen, S. Wang, *Nat. Commun.* **2024**, *15*, 2481.

[60] K. Liu, X. Zhang, J. Li, Y. Liu, M. Wang, H. Cui, *Green Chem.* **2024**, *26*, 4677-4683.

[61] T. Yang, H. Lv, Q. Quan, X. Li, H. Lu, X. Cui, G. Liu, L. Jiang, *Appl. Surf. Sci.* **2023**, *615*, 156360.

[62] Y. Luo, P. Wang, G. Zhang, S. Wu, Z. Chen, H. Ranganathan, S. Sun, Z. Shi, *Chem. Eng. J.* **2023**, *454*, 140061-140072.

[63] W. Zang, T. Sun, T. Yang, S. Xi, M. Waqar, Z. Kou, Z. Lyu, Y. P. Feng, J. Wang, S. J. Pennycook, *Adv. Mater.* **2020**, *33*, 2003846-2003853.

[64] Z. Yu, Y. Li, V. Martin-Diaconescu, L. Simonelli, J. Ruiz Esquius, I. Amorim, A. Araujo, L. Meng, J. L. Faria, L. Liu, *Adv. Funct. Mater.* **2022**, *32*, 2206138.

[65] X. H. Wang, Y. Ling, B. Wu, B. L. Li, X. L. Li, J. L. Lei, N. B. Li, H. Q. Luo, *Nano Energy* **2021**, *87*, 106160.

[66] J. Chen, L. Zhang, J. Li, X. He, Y. Zheng, S. Sun, X. Fang, D. Zheng, Y. Luo, Y. Wang, J. Zhang, L. Xie, Z. Cai, Y. Sun, A. A. Alshehri, Q. Kong, C. Tang, X. Sun, *J. Mater. Chem. A* **2023**, *11*, 1116-1122.

[67] R. Kalusulingam, M. Mariyaselvakumar, S. Mathi, S. Arokiasamy, T. S. Mikhailova, G. M. Alexandrovich, I. V. Pankov, A. A. Jeffery, T. N. Myasoedova, *J. Alloy. Compd.* **2024**, *1002*, 175389.

[68] W. Liu, J. Zhao, L. Dai, Y. Qi, K. Liang, J. Bao, Y. Ren, *Inorg. Chem.* **2024**, *63*, 6016-6025.

[69] Z. Cui, Z. Yan, J. Yin, W. Wang, M.-E. Yue, Z. Li, *J. Colloid Interface Sci.* **2023**, *652*, 1117-1125.

[70] X. Wang, G. Liu, D. Zhang, S. Han, J. Yin, J. Jiang, W. Wang, Z. Li, *J. Colloid Interface Sci.* **2023**, *652*, 1217-1227.

[71] C. Fu, W. Hao, J. Fan, Q. Zhang, Y. Guo, J. Fan, Z. Chen, G. Li, *Small* **2023**, *19*, 2205689.

[72] R. Andaveh, A. Sabour Rouhaghdam, J. Ai, M. Maleki, K. Wang, A. Seif, G. Barati Darband, J. Li, *Appl. Catal. B: Environ.* **2023**, *325*, 122355.

[73] Y. Yu, J. Li, J. Luo, Z. Kang, C. Jia, Z. Liu, W. Huang, Q. Chen, P. Deng, Y. Shen, X. Tian, *Mater. Today Nano* **2022**, *18*, 100216.

[74] R. Liang, J. Fan, F. Lei, P. Li, C. Fu, Z. Lu, W. Hao, *J. Colloid Interface Sci.* **2023**, *645*, 227-240.

[75] K. Jiang, W. Liu, W. Lai, M. Wang, Q. Li, Z. Wang, J. Yuan, Y. Deng, J. Bao, H. Ji, *Inorg. Chem.* **2021**, *60*, 17371-17378.

[76] S. Gopalakrishnan, G. Anandha babu, S. Harish, E. S. Kumar, M. Navaneethan, *Chemosphere* **2024**, *350*, 141016.

[77] R. Kaur, A. Gaur, Aashi, V. Pundir, J. Sharma, C. Bera, V. Bagchi, *Energy Fuels* **2024**, *38*, 11137-11147.

[78] S.-Y. Lu, L. Wang, C. Wu, J. Zhang, W. Dou, T. Hu, R. Wang, Y. Liu, Q. Yang, M. Jin, *Inorg. Chem. Front.* **2024**, *11*, 3187-3199.

[79] L. Qian, Y. Zhu, H. Hu, Y. Zheng, Z. Yuan, Y. Dai, T. Zhang, D. Yang, S. Xue, F. Qiu, *J. Colloid Interface Sci.* **2024**, *669*, 935-943.

[80] T. Wu, E. Song, S. Zhang, M. Luo, C. Zhao, W. Zhao, J. Liu, F. Huang, *Adv. Mater.* **2021**, *34*, 2108505.

[81] D. Senthil Raja, H.-W. Lin, S.-Y. Lu, *Nano Energy* **2019**, *57*, 1-13.

[82] X. Cheng, C. Lei, J. Yang, B. Yang, Z. Li, J. Lu, X. Zhang, L. Lei, Y. Hou, K. Ostrikov, *ChemElectroChem* **2018**, *5*, 3866-3872.

[83] Y. Liu, B. Zhou, Y. Zhang, W. Xiao, B. Li, Z. Wu, L. Wang, *J. Colloid Interface Sci.* **2023**, *637*, 104-111.

[84] X. Zhou, Y. Mo, F. Yu, L. Liao, X. Yong, F. Zhang, D. Li, Q. Zhou, T. Sheng, H. Zhou, *Adv. Funct. Mater.* **2022**, *33*, 2209465.

[85] L. Wu, L. Yu, B. McElhenny, X. Xing, D. Luo, F. Zhang, J. Bao, S. Chen, Z. Ren, *Appl. Catal. B: Environ.* **2021**, *294*, 120256-120264.

[86] C. Yang, K. Dong, L. Zhang, X. He, J. Chen, S. Sun, M. Yue, H. Zhang, M. Zhang, D. Zheng, Y. Luo, B. Ying, Q. Liu, A. M. Asiri, M. S. Hamdy, X. Sun, *Inorg. Chem.* **2023**, *62*, 7976-7981.

[87] Y. Du, Q. Li, M. Liu, D. Liu, W. Xiao, Z. Xiao, Z. Li, Y. Yamauchi, S. M. Osman, Z. Wu, L. Wang, *Chem. Eng. J.* **2023**, 146057.

[88] W. Ma, D. Li, L. Liao, H. Zhou, F. Zhang, X. Zhou, Y. Mo, F. Yu, *Small* **2023**, *19*, 2207082.

[89] L. Liao, D. Li, Y. Zhang, Y. Zhang, F. Yu, L. Yang, X. Wang, D. Tang, H. Zhou, *Adv. Mater.* **2024**, 2405852.

[90] Y. He, Y. Hu, Z. Zhu, J. Li, Y. Huang, S. Zhang, M. S. Balogun, Y. Tong, *Chem. Eng. J.* **2024**, *489*, 151348.

[91] T. Van Tam, K. Chandra Bhamu, M. Jae Kim, S. Gu Kang, J. Suk Chung, S. Hyun Hur, W. Mook Choi, *Chem. Eng. J.* **2024**, *480*, 148190.
